# Resident memory T cells in tumor-distant tissues fortify against metastasis formation

**DOI:** 10.1016/j.celrep.2021.109118

**Published:** 2021-05-11

**Authors:** Laura S. Christian, Liuyang Wang, Bryan Lim, Dachuan Deng, Haiyang Wu, Xiao-Fan Wang, Qi-Jing Li

**Affiliations:** 1 Department of Immunology, Duke University Medical Center, Durham, NC 27710, USA; 2 Department of Molecular Genetics and Microbiology, Duke University Medical Center, Durham, NC 27710, USA; 3 TCRCure (TianKeYa) Biopharma, Ltd., Durham, NC 27701, USA; 4 Departments of Pharmacology and Cancer Biology, Duke University Medical Center, Durham, NC 27710, USA; 5 These authors contributed equally; 6 Lead contact

## Abstract

As a critical machinery for rapid pathogen removal, resident memory T cells (T_RM_s) are locally generated after the initial encounter. However, their development accompanying tumorigenesis remains elusive. Using a murine breast cancer model, we show that T_RM_s develop in the tumor, the contralateral mammary mucosa, and the pre-metastatic lung. Single-cell RNA sequencing of T_RM_s reveals two phenotypically distinct populations representing their active versus quiescent phases. These T_RM_s in different tissue compartments share the same TCR clonotypes and transcriptomes with a subset of intratumoral effector/effector memory T cells (T_Eff/EM_s), indicating their developmental ontogeny. Furthermore, CXCL16 is highly produced by tumor cells and CXCR6^−^ T_Eff/EM_s are the major subset preferentially egressing the tumor to form distant T_RM_s. Functionally, releasing CXCR6 retention in the primary tumor amplifies tumor-derived T_RM_s in the lung and leads to superior protection against metastases. This immunologic fortification suggests a potential strategy to prevent metastasis in clinical oncology.

## In brief

Christian et al. show that tumor-derived resident memory T cells (T_RM_s) are developed in tumors, as well as in distant and tumor-free mucosal tissues. Distant T_RM_s are developed from T_Eff/EM_ precursors that are trapped within the tumor. The release of such retention can be achieved by CXCR6/CXCL16 blockade, which leads to prevention against metastasis.

## Graphical Abstract

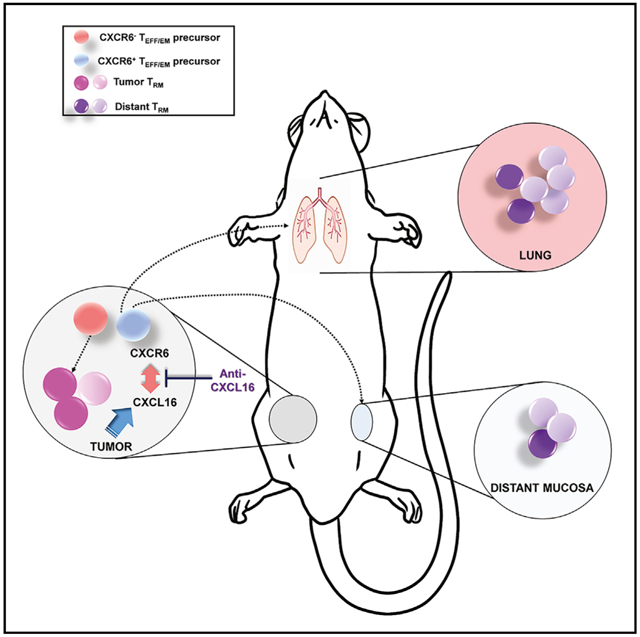

## INTRODUCTION

The formation of memory T cells is the hallmark of adaptive immunity, which provides rapid and robust protection against bacteria, virus, or tumor during antigen re-encounter. Classically, memory T cells are categorized into two major phenotypes: central memory T cells (T_CM_s) and effector memory T cells (T_EM_s) (Sallusto et al., 1999). T_CM_s are long-lived, quiescent, and stem cell-like. They bear the chemokine receptor CCR7 and the cell adhesion molecule CD62L, allowing them to enter and patrol secondary lymphoid organs (Unsoeld et al., 2002; von Andrian and Mackay, 2000). Upon antigen recognition, they differentiate with multipotent capacity (Williams and Bevan, 2007). In contrast, T_EM_s have a shorter lifespan but are more poised for activation. T_EM_s circulate and enter peripheral non-lymphoid tissues, where they exert effector functions for swift and robust pathogen control (Fuhlbrigge et al., 1997; Mackay et al., 1992). A decade ago, the guardian functions of T_EM_s in non-lymphoid tissues were reattributed to a novel non-circulating subset of memory T cells, resident memory T cells (T_RM_s) (Masopust et al., 2001). Using CD103 (*Itgae*), a specified integrin molecule that binds E-cadherin on the epithelial barrier (Cepek et al., 1994), T_RM_s reside in mucosal tissues and are an integral component of the adaptive immune machinery against viral re-challenge, including vaccinia virus, influenza, and herpes simplex virus (HSV) (Ariotti et al., 2014; Iijima and Iwasaki, 2015; Jiang et al., 2012; Teijaro et al., 2011; Wu et al., 2014). Upon encountering infected cells, such as T_EM_s, T_RM_s activate quickly. However, unlike T_EM_s, T_RM_s release chemokines to recruit T cells, including circulating T_EM_s, to the infected tissue for intensified immune protection (Schenkel and Masopust, 2014).

While their pivotal roles in the antiviral response are well appreciated, the ontogeny of T_RM_s remains elusive. In a study using a skin immunization model and high-throughput T cell receptor-β (TCR-β) sequencing, T_CM_s and T_RM_s were found to develop from the same naive T cell clone (Gaide et al., 2015). A single-cell RNA sequencing (scRNA-seq) study with the acute lymphocytic choriomeningitis virus (LCMV) infection model found that early effector cells with high interleukin-2 receptor α (IL-2rα) and Ezh2 expression were predisposed to become T_RM_s (Kurd et al., 2020). Specifically, Gerlach et al. (2016) showed that during LCMV infection, CX3CR1^−^ effector cells were a common precursor of T_CM_s and T_RM_s. While circulating CX3CR1^int^ peripheral memory cells (T_PM_s) can also survey the tissues, T_EM_s arise from CX3CR1^hi^ cells and are prohibited from tissue entry. In both vaccinia virus (VACV) infection and B16 melanoma transplantation models, transferred T_CM_ cells were found to differentiate directly into T_RM_s (Enamorado et al., 2017). By contrast, in an influenza airway infection model, only transferred T_EM_s were able to become lung T_RM_s (Slütter et al., 2017). More recently, with a *Listeria monocytogenes* (LM) infection model, circulating KLRG1^−^ cells are suggested as a common precursor subset for all memory T cell populations, including T_CM_, T_EM_, CX3CR1^int^ T_PM_, and T_RM_ (Herndler-Brand-stetter et al., 2018). These data suggest that the model of T_RM_ induction and their residing tissue environment play a significant role in T_RM_ development.

Tumorigenesis is a chronic process and established tumors are composed of a complex microenvironment. Consequently, some identified characteristics of tumor T_RM_s are surprising given our current knowledge of T cell memory. Using scRNA-seq, T_RM_s in human breast tumors were shown to be similar to terminally differentiated intratumoral effector/effector memory T cells (T_Eff/EM_s); they were Klrc1^hi^PD-1^hi^Tim-3^hi^, but enriched with cytolytic molecules such as granzyme B (Savas et al., 2018). T_RM_s from murine B16 tumors could separate into two populations: one being Blimp1^hi^ and short-lived, similar to the population found in human breast tumors, and the other, Id3^hi^, is a marker of long-lived T_RM_ cells (Milner et al., 2020). Nevertheless, a consensus has been reached that T_RM_s play an important role in overall antitumor immunity. In mice, the presence of tumor antigen-specific T_RM_s in the skin, either induced by engineered HSV (Park et al., 2019) or established through previous tumor challenge (Malik et al., 2017), protected mice from transplanted B16 melanoma. For patients with breast, lung, or ovarian cancer, the abundance of CD8^+^CD103^+^ T_RM_s strongly correlated with longer disease-free survival (Djenidi et al., 2015; Wang et al., 2016; Webb et al., 2014). Since tumor metastases present a much more prominent threat to patients’ lives than their primary tumor, it is critical to establish whether tumor-specific T_RM_s can be induced outside the primary tumor site and whether they play protective roles against metastasis.

Using high-throughput TCR-β repertoire sequencing on a cohort of gastric cancer patients, our group previously identified CD8^+^CD103^+^ T_RM_s in the tumor-adjacent mucosa of gastric tumors. Compared with their peripheral counterparts, tumor-infiltrating T cells had a restricted TCR-β repertoire and oligoclonal expansion, resulting in reduced clonotype diversity. Through repertoire analyses of T cells within the adjacent, tumor-free mucosal area, we detected a significant proportion of CD103^+^ T_RM_s that share the same TCR with highly expanded T cell clones found in the tumor. Furthermore, the diversity of these T cells correlated with patients’ long-term prognosis, suggesting their protective role against gastric cancer recurrence and metastasis (Jia et al., 2015).

In this study, using a murine breast cancer model, we used scRNA-seq to analyze the heterogeneity of memory T cell subsets in the tumor. Specifically, we focused on the lineage progression of tumor T_RM_s. We characterized fully differentiated T_RM_s residing in the tumor and distant (non-tumor-adjacent) mammary mucosal tissues. Through single-cell transcriptome mapping and TCR clonotype tracking, we determined that distant mucosal T_RM_s derived from a particular T cell effector/effector memory intratumoral population that lacked CXCR6 expression. Releasing T cells from primary breast tumors by breaking CXCR6-mediated retention led to enhanced protection against tumor metastasis in the distant lung.

## RESULTS

### Resident memory T cells develop in tumor and distant tumor-free mucosal tissues

**Figure 1.**
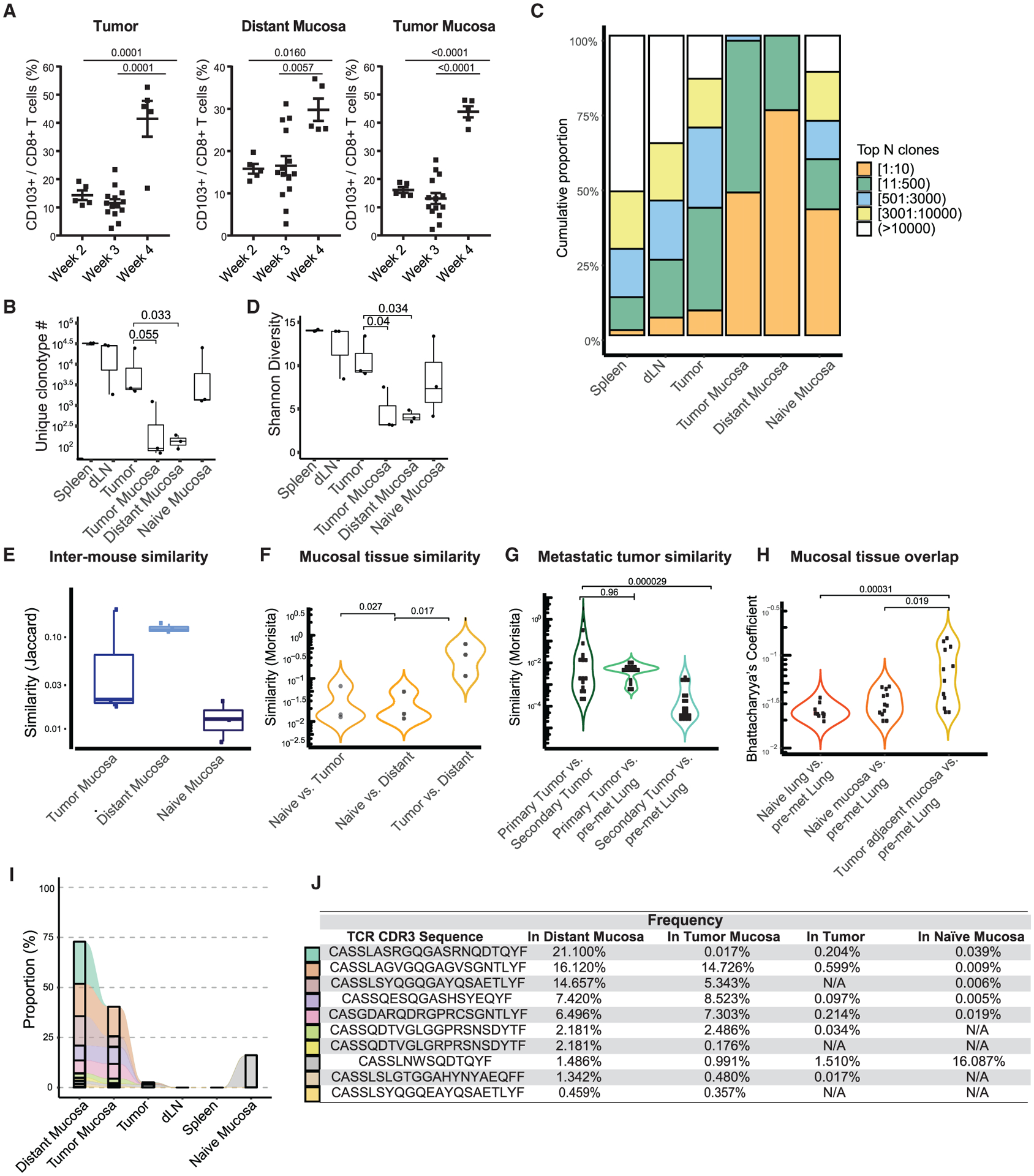
TCR-b repertoire sequencing identifies extensive sharing between tumor and distant mucosa T_RM_s (A) Flow cytometry analysis of CD8^+^CD103^+^T_RM_s in the tumor, tumor mucosa, and distant mucosa. One-way ANOVA with Tukey’s multiple comparison test, p < 0.05. Data are pooled over 3 independent experiments. (B) Analysis of the unique clonotype number between samples. Wilcoxon signed rank test, p < 0.05. (C) Clonal T cell expansion in the tumor and tumor-associated tissues. (D) Shannon diversity index shows a reduction of CD8^+^ T cell diversity in the tumor, tumor mucosa, and distant mucosa. Wilcoxon signed rank test, p < 0.05. (E) Jaccard similarity analysis compares distinct T cell clones in the tissues of individual mice. (F) Global T cell similarity analysis uses the Morisita index to measure the presence and abundance of clonotypes between mucosal tissues. Wilcoxon signed rank test, p < 0.05. (G) Morisita index evaluates clonotype similarity between primary and secondary (metastatic) lung tumor and pre-metastatic lung tissues. Wilcoxon signed rank text, p < 0.05. (H) Bhattacharyya’s coefficient analysis evaluates the clonal overlap between mammary and lung mucosal tissues. Wilcoxon signed rank test, p < 0.05. (I) Analysis of the 10 most expanded TCRs in the distant mucosa and their overlap between other tissues. (J) TCR CDR3 sequences of the top 10 most expanded distant mucosa clones are listed on the left and their frequency in other tissues is displayed as a percentage. Bars represent means ± SEMs and symbols represent individual mice.

To study tumor-resident memory CD8^+^ T cells, we used the orthotopic 4T1 triple negative breast tumor model. We titrated tumor-inducing doses of 4T1 cells and determined that implanting 100 4T1 cells into the mammary fat pad had 100% penetrance to form primary tumors in 3 weeks, without visibly detectable lung metastases until 5 weeks (Figure S1A). At different time points post-tumor inoculation, we harvested the tumor and tumor-adjacent mucosa to assess the development of tumor T_RM_s (Figure S1B). While we initially included the distant (tumor-free) mammary gland mucosa as a T_RM_-free control, we found that CD8^+^CD103^+^ T_RM_s developed in all tissues and their frequency increased with tumor growth, even in the distant mucosa (Figure 1A).

To assess the overall structure of the T cell repertoire within different compartments, we performed TCR-β deep sequencing without T cell purification, on multiple tissues collected from tumor-bearing mice. The mammary gland from tumor-naive mice was included as a control (Table S1). Within the tissues from tumor-bearing mice, using the spleen as the standard, we found that the number of unique TCR clonotypes was lower in the tumor and further contracted in the tumor-adjacent and distant mammary gland mucosa (Figure 1B). This clonotype contraction was accompanied by oligoclonal expansion. Approximately 10,000 different TCR clones occupied ~50% of the repertoire space in the spleen; a similar amount of space was occupied by slightly more than 500 clonotypes in the tumor and 10 clonotypes in the tumor-adjacent mucosa; most striking, 10 different clones of T cells accounted for 75% of the total T cell repertoire within the distant mucosa (Figure 1C). Accordingly, when the TCR diversity was measured by Shannon entropy, which is determined by both the clonotype number and expansion level of individual clonotypes, we found that T cell diversity was reduced in the tumor, tumor-adjacent mucosa, and tumor-distant mucosa as a cascade (Figure 1D). Evidently, as also found in the mammary gland mucosa of female monkeys, the mammary mucosa of tumor-naive mice harbored T cells (Sircar et al., 2010). However, T cells residing in this environment have a much more diverse repertoire compared to tumor-bearing mice (Figures 1B–1D). In summary, these data suggest that in tumor-bearing mice, T cell residency within the mammary gland mucosa selects for a limited population of T cells.

To track the clonal origins of these T cells, we compared the similarity of TCR clonotypes within the mucosal tissue compartments among different mice. Using the Jaccard index, we measured the ratio of clonotypes overlapping between two specific compartments. When the mammary gland mucosa was compared among different animals, more T cell clonotypes in the tumor-adjacent mucosa were shared among different tumor-bearing mice than among individual tumor-naive mice. This clonotype sharing was more evident for T cells in the distant mammary gland tissues of tumor-bearing mice (Figure 1E). Considering that only select clonotypes reside within the mammary gland mucosa, this heightened TCR similarity among tumor-bearing mice suggests that some common antigens produced by 4T1 tumors may play a role in this selection.

We also performed a global T cell similarity analysis between different types of mucosal tissues. Using the Morisita index, we took both clonotype amounts and abundance into account (Venturi et al., 2008). We found little repertoire similarity between T cells within the tumor-naive and tumor-adjacent mucosa or between T cells from the tumor-naive and tumor-distant mucosa. However, the overall similarity between T cells in the tumor-adjacent and distant mucosa from tumor-bearing mice was quite high (Figure 1F). Since the lung is the primary site of 4T1 spontaneous metastasis, we harvested the pre-metastatic lung mucosa tissue at 3 weeks post-tumor inoculation (Figure S2), as well as the later-developed lung metastatic (secondary tumors) for TCR repertoire analysis. We found that clonotypes overlapped between the primary tumor in the breast and metastatic tumors in the lung. The clonotype sharing, especially for the dominant clones favored by the Morisita index, between the primary tumor and pre-metastatic lung was as high as the sharing between the primary and secondary tumors (Figure 1G). This coordinates with our similarity findings between the primary tumor and the distant mammary gland. In addition, the similarity between the pre-metastatic lung and the primary tumor was higher than that between the pre-metastatic lung and the lung metastases. This suggested that either there are new infiltrates after the establishment of metastasis or there are T cell clonotypes that expand or contract, resulting in the change in dominant T cell clones. Furthermore, the overall sharing of clonotypes (measured by Bhattacharyya’s coefficient after normalization) between the pre-metastatic lung tissues and distant mammary gland mucosa was significantly elevated compared to the sharing between the pre-metastatic lung and either the mammary gland mucosa or lung tissues from tumor-naive mice (Figure 1H). These data strongly suggested that before the establishment of metastases, the same expanded T cell clones egressed the primary tumor and infiltrated the distant mammary gland and pre-metastatic lung tissue.

Zooming in on the most abundant (top 10) clonotypes in the distant mucosa, we found that each individual clone could be identified in the tumor-adjacent mucosa and 6 of 10 were highly expanded (>0.5%) clones (Figures 1I and 1J) (Jia et al., 2015). In addition, 7 of 10 of these clones could also be identified within the 4T1 tumor (Figures 1I and 1J). Notably, these TCRs, except one, could not be found in the mucosa of tumor-naive mice. This deep sequencing-aided clonal lineage tracing indicated that resident T cells found in the tumor adjacent and distant mucosa share common precursors, which most likely originate from T cells in the tumor.

### scRNA-seq dissects intratumoral T_Eff/EM_ heterogeneity

**Figure 2.**
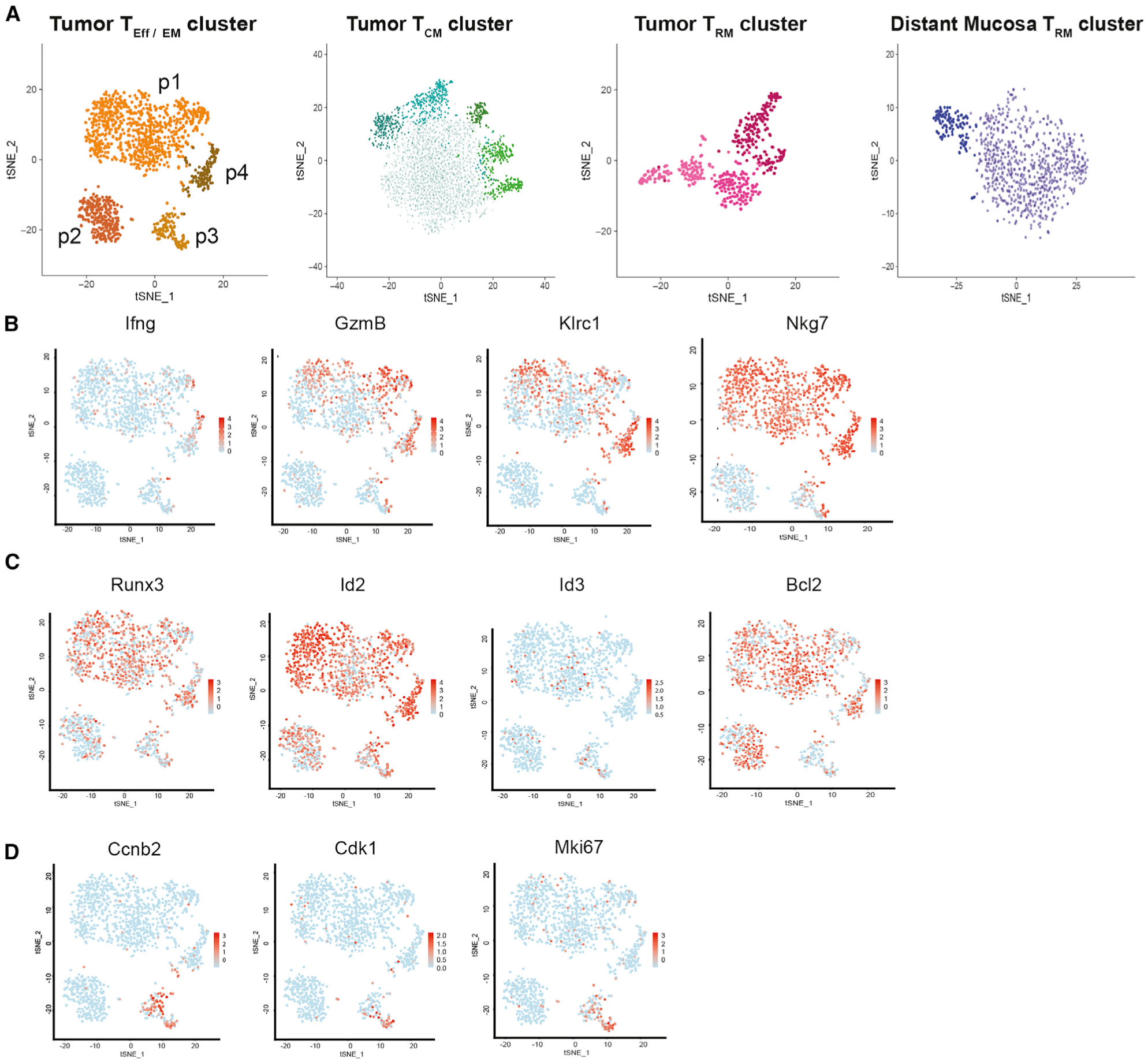
Single-cell RNA sequencing reveals intratumor T_Eff/EM_ heterogeneity (A) Independent tSNE plots of sorted live TCR-β^+^CD44^+^CD62L^−^CD69^−^CD103^−^ tumor T_Eff/EM_, TCR-β^+^CD44^+^CD62L^+^CD69^−^CD103^−^ tumor T_CM_, TCR-β^+^CD44^+^CD62L^−^CD69^+^CD103^+^ tumor T_RM_, and TCR-β^+^CD44^+^CD69^+^CD103^+^ distant mucosa T_RM_ single-cell transcriptomes obtained from 10 tumors and matched distant mucosa. Each dot represents a cell; each color indicates a distinct CD8^+^ T cell cluster. (B–D) T_Eff/EM_ CD8^+^ clusters are labeled p1–p4, and the expression of (B) effector molecules and activation markers, (C) transcription factors, and (D) proliferation markers in the tumor T_EFF/EM_ clusters are shown.

While the heterogeneity of CD103^+^ T_RM_s is widely discussed in the literature, the characteristics of T_RM_ subpopulations within the tumor or distant tissues of tumor-bearing mice are less well defined. To characterize tumoral T_RM_s, scRNA-seq technology provides unprecedented analytical power and allows us to dissect cells with thousands of dimensions. With the hypothesis that particular intratumoral T cells are precursors for T_RM_ development, we extended our scRNA-seq approach to include various effector and memory T cell populations to comprehensively depict their transcriptomic program. From the tumor and distant mucosa of mice bearing established 4T1 tumors, we flow sorted distinct memory T cell populations using a strict gating strategy (Figure S3) (Buenrostro et al., 2018). We constructed single-cell cDNA libraries from paired intratumoral sorted TCR-β^+^CD44^+^CD62L^−^CD69^+^CD103^+^ cells (T_RM_s), mucosal sorted TCR-β^+^CD44^+^ CD69^+^CD103^+^ cells (distant mucosa T_RM_s), intratumoral TCR-β^+^CD44^+^CD62L^+^CD69^−^CD103^−^ cells (T_CM_s), and TCR-β^+^CD44^+^CD62L^−^CD69^−^CD103^−^ cells (T_EM_s) (Table S2). After data processing and normalization, we performed unbiased clustering and visualized the CD8^+^ clusters using t-distributed stochastic neighbor embedding (t-SNE; Figure 2A) (Kobak and Berens, 2019). Through this unsupervised analysis, we found that all four samples could distinctly separate into multiple clusters, confirming continued heterogeneity among the single-cell subsets.

In comparison to T_CM_s, intratumoral T_Eff/EM_s were a more heterogeneous population, which could largely be divided into four subsets, p1–p4. The effector molecules *Ifng* and *GzmB* were highly expressed in p4, had heterogeneous expression in p1 and p3, but were largely absent from p2. Similar expression patterns were applied to well-known effector surface markers for cytolytic T cells such as *Klrc1* and *Nkg7* (Figure 2B). From this, we reasoned that p4 is highly enriched by effector cells. All four subsets highly expressed the transcription factor *Runx3*, which is essential for the cytotoxic program (Cruz-Guilloty et al., 2009), as well as *Id2*, which is upregulated to support the effector phase of cytotoxic T lymphocytes (CTLs) (Cannarile et al., 2006) responses. Comparing p2 to p4, the density of Runx3- and Id2-expressing cells was slightly lower in p2. This was accompanied by an opposite pattern of Id3, a transcription factor whose expression is crucial for effector memory development (Yang et al., 2011), which is absent in p4 (Figure 2C). We concluded that T_Eff/EM_ p4 is enriched by differentiated effector CTLs and T cells in the p2 subset are at a more advanced T_EM_ stage. *Bcl2*, a transcription factor for T cell survival in the effector and memory phases, was abundant in most sorted T_Eff/EM_ populations except p3. After assessing the cell-cycle programs, we determined that p3 was a highly proliferative subset, as made evident by the expression of genes restrictively expressed in S and M phases, such as *Ccnb2*, *Cdk1*, and *Mki67* (Figure 2D). We reasoned that the p2 subset is constituted by proliferative and less differentiated T_Eff_ cells.

### Tumor and distant mucosa T_RM_s comprise two distinct populations that resemble either T_EM_s or T_CM_s

**Figure 3.**
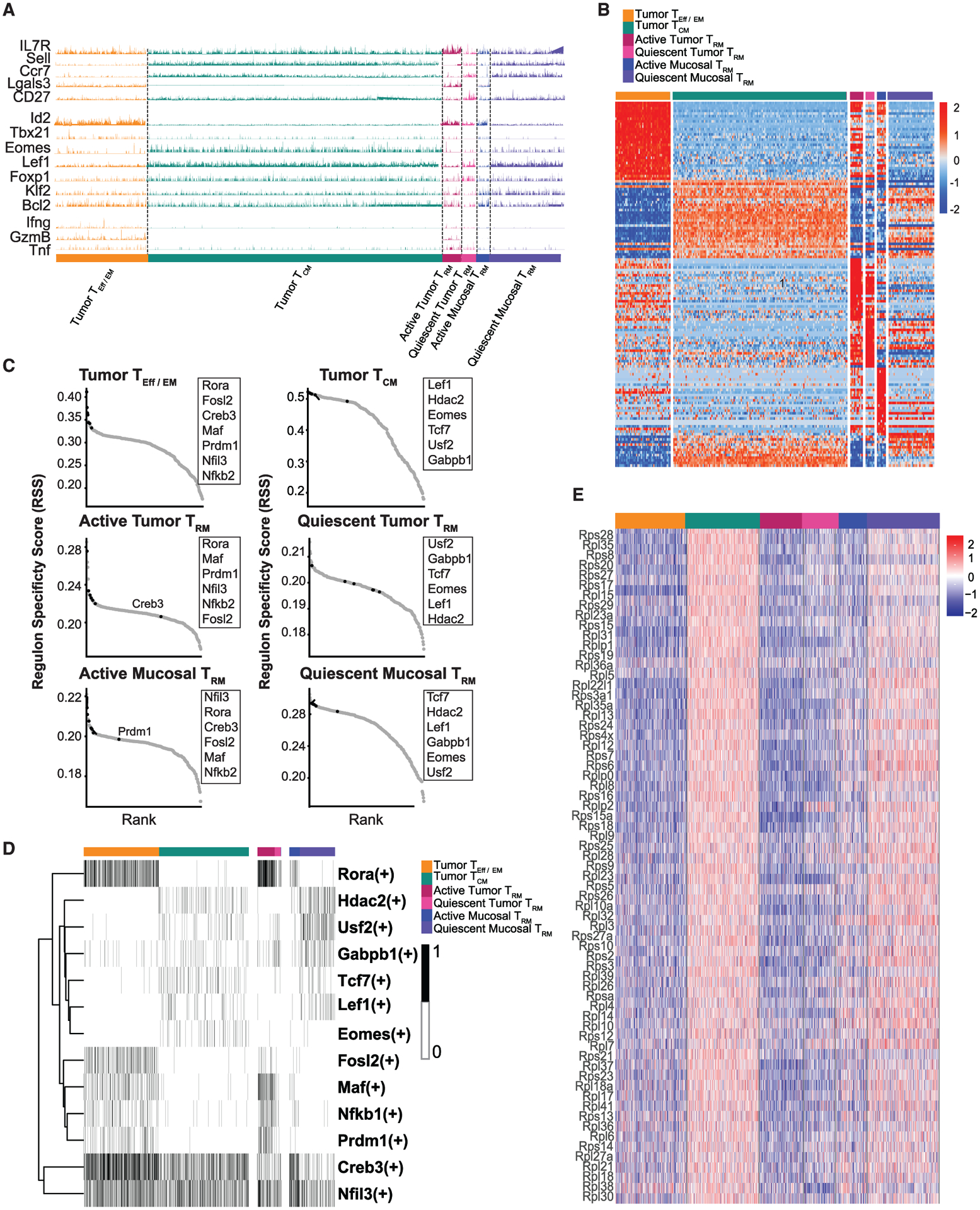
Two distinct subsets that resemble either T_EM_s or T_CM_scomprise tumor and distant mucosa T_RM_s (A) GeneTrac analysis of surface markers, transcription factors, and effector molecules among the major tumor T_Eff/EM_, T_CM_, T_RM_, and distant T_RM_ populations. (B) Heatmap of top 100 DEGs between the tumor T_Eff/EM_ and tumor T_CM_s shows the global gene expression pattern between these circulating memory T cells and active and quiescent T_RM_s. (C) SCENIC regulon analysis reveals an enrichment of the top regulons between tumor T_Eff/EM_ and active T_RM_s and between tumor T_CM_s and quiescent T_RM_s. (D) Binary heatmap for top regulons is plotted for each cluster. (E) A global enrichment of ribosome genes associates with tumor T_CM_ and quiescent T_RM_ subsets.

Of most interest to the present study is that both the tumor T_RM_ and distant mucosa T_RM_ populations independently clustered into three and two major groups, respectively. As expected, the tumor and distant mucosa T_RM_ populations showed high expression of *Itgae* (CD103) and low expression of *S1PR1*, confirming their tendency to reside within the tissue (Figure S4). Choosing the major subsets of T_CM_s and T_EM_s as references (p1 of T_Eff/EM_), we interpreted these T_RM_ subpopulations using a candidate approach by evaluating their surface markers, transcription factors, and effector molecules. We found a subset of T_RM_s that shared lineage characteristics with T_EM_s, indicating that this subset of T_RM_s was maintained at a functionally active stage, hereafter called “active T_RM_s.” Specifically, we noted that (1) in active T_RM_s, similar to their T_Eff/EM_ counterparts, *Sell* expression was completely suppressed (Sallusto et al., 1999) and *Ccr7* expression was severely reduced. Notably, the binary expression of *Lgals3* (galectin-3) could distinguish T_EM_s from T_CM_s, as well as active T_RM_s from quiescent T_RM_s. This is consistent with previous findings that optimal galectin-3 induction requires TCR signaling (Joo et al., 2001), and in the tumor microenvironment, galectin-3 is differentially expressed on the surface of tumor-antigen activated CD8^+^ T cells, but is absent in resting T cells (Joo et al., 2001; Kouo et al., 2015). (2) Similar to that in T_Eff/EM_s, *Id2* (Cannarile et al., 2006) was highly elevated in the active T_RM_ subsets. Of note, since the CD8^+^ T cells in our analysis are memory phenotype enriched, transcription factors that preferentially support early effector function, such as *Tbx21* (T-bet) (Intlekofer et al., 2005), could be detected occasionally. Nevertheless, more frequent *Tbx21* expression was observed in active T_RM_s than quiescent T_RM_s. (3) Similar to T_Eff/EM_s, *Ifng* and *GzmB* were preferentially expressed in active tumor T_RM_s (Figure 3A).

**Figure 4.**
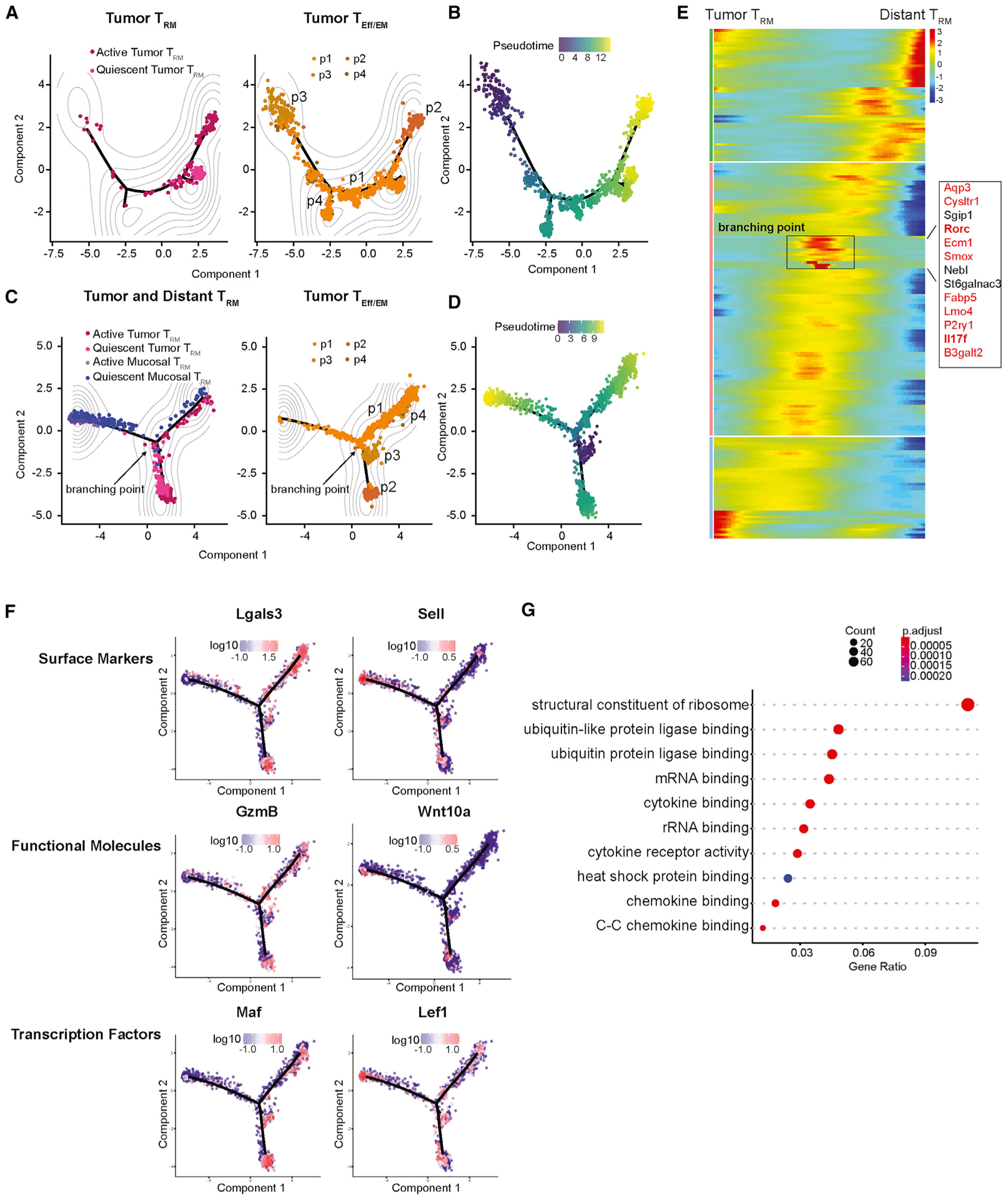
Tissue environment plays a significant role in shaping the formation of T_RM_s (A and B) Monocle2 analysis shows the lineage relationship and progression between the 4 tumor T_Eff/EM_ CD8^+^ subsets and tumor T_RM_s in (A) contour and (B) pseudotime plot. The main proliferating population (tumor T_Eff/EM_ p3) was used as the starting point of pseudotime progression in all of the analyses. (C and D) Contour (C) and pseudotime (D) analysis of the tumor T_Eff/EM_ populations, tumor T_RM_s, and distant T_RM_s shows that these populations distinctly separate by their tissue of origin. The divergence of this lineage path is identified at the branching point. (E) Heatmap of gene expression of the individual T cell subsets approaching and leaving this branch point. A total of 13 critical genes define this branch point, the majority of which (in red) are signature genes identified in the Th17 lineage. (F) Surface markers *Lgals3* and *Sell*, functional molecules *GzmB* and *Wnt10a*, and transcription factors *Maf* and *Lef1* were projected back on the pseudotime space to exemplify the differences between tumor T_RM_s (left column) and distant T_RM_s (right column) (G) Gene Ontology analysis reveals that ribosome pathways were enriched in the distant T_RM_ population, while cytokine and chemokine signaling pathways were enriched in tumor T_RM_s.

Reciprocally, within both the tumor and distant mammary gland mucosal compartments, we found another subset of T_RM_s that obtained gene expression features resembling T_CM_s, representing a quiescent, long-lived resident memory T cell population, hereafter called “quiescent T_RM_s.” Quiescent T_RM_s could be found in both the tumor and distant mucosa compartments. Specifically, in quiescent T_RM_s, we noted the following: (1) the T_CM_ surface markers *Sell* (CD62L) and *CCR7* were highly expressed. While CD27 was widely expressed in all of the selected subsets, its expression was elevated in the quiescent subsets, suggesting its naive-like feature (van Lier et al., 1987). (2) T_CM_-associated transcription factors such as *Eomes* (Pearce et al., 2003), *Lef1* (Zhou and Xue, 2012), and *Foxp1*(Feng et al., 2010) were highly expressed in the quiescent T_RM_ population. (3) As in T_CM_s, the expression of functional molecules associated with cytolytic killing, such as *Ifng*, *GzmB*, and *Tnf* were silenced (Figure 4A). T_RM_s, both in the tumor and mammary gland mucosal compartments, could be largely divided into two functional phenotypes based on their signature gene expression.

The T_Eff/EM_ versus T_CM_ phenotypic division was also validated by global differences in their transcriptomes. We took the expression levels of the top 100 differentially expressed genes (DEGs) in the intratumoral T_EM_ and T_CM_ populations as a benchmark. When this unbiased control was applied to different T_RM_ subsets, it depicted the major differences between active and quiescent T_RM_s (Figure 3B).

To examine whether these transcriptomic differences represented a globally orchestrated differentiation event, we performed transcriptomic regulon analysis. The SCENIC (single-cell regulatory network inference and clustering) algorithm was used to identify co-expressed gene modules that share common *cis-*regulatory elements for a specific transcription factor (Aibar et al., 2017). Based on p values that reflect the co-expression co-efficients and enrichment of *cis-*regulatory elements, we ranked the transcription factor regulons in classical T_Eff/EM_s and T_CM_s. Although the ranking order may vary, the majority of the top-ranked T_Eff/EM_ regulons such as *Rora* (Best et al., 2013), *Fosl2* (Ciofani et al., 2012), *Creb3* (Chan et al., 2011), *Maf* (Ciofani et al., 2012), *Prdm1* (Kallies et al., 2009), *Nfil3* (Kashiwada et al., 2011), and *Nfkb1* (Best et al., 2013) were also identified as top-ranked regulons for active T_RM_s in the tumor and distant mammary mucosa. Reciprocally, all top-ranked T_CM_ regulons, such as *Lef1* (Zhou and Xue, 2012), *Hdac2* (Shin et al., 2013), *Eomes* (Pearce et al., 2003), *Tcf7* (Zhou et al., 2010), *Usf2* (Suo et al., 2018), and *Gabpb1* (Luo et al., 2017), were significantly enriched in the transcriptome of quiescent T_RM_s (Figures 3C and 3D). This analysis revealed that the transcriptomic distinctions between active and quiescent T_RM_s are controlled by the same underlying gene regulatory networks that specify T_EM_ versus T_CM_ development.

While investigating the DEGs between these memory T cell populations, we found an unexpected enrichment of a large cluster of ribosome-related genes that were distinct between intratumor T_Eff/EM_s and T_CM_s. While this differential expression was less pronounced between intratumoral T_RM_s, expression of these ribosome genes was dramatically upregulated in the quiescent distant T_RM_ population compared to active distant T_RM_s (Figure 3E). The association between an upregulated ribosome gene profile and a relatively quiescent T cell phenotype was a common feature identified through our analyses.

### Tissue environment plays a significant role in shaping the transcriptome of T_RM_s

To trace the developmental path of T_RM_s, we subjected all tumor T_Eff/EM_ and T_RM_ cells to the Monocle2 algorithm for pseudotime analysis (Qiu et al., 2017; Trapnell et al., 2014). Our assumption was that within the tumor, T cells differentiate or develop asynchronously. The moment of sample collection represents a snapshot, in which each individual T cell may be fixed at a specific stage of differentiation or development. Consequently, differentiation and developmental processes of the T cells can be revealed by arranging individual cells on a time trajectory based on their gradual and continuous transcriptomic transition. Hence, pseudotime analysis allowed us to determine the relative position of each individual cell on this time trajectory. Since the relative positioning of cells cannot automatically determine the beginning or end of the pseudotime, we designated the least differentiated T_Eff/EM_ population with the highest proliferative capacity (T_Eff/EM_ p3; Figure 2D) as the starting point. This is a reasonable assumption because T cell proliferation in the effector phase proceeds terminal effector and memory T cell differentiation, including the development of T_RM_s._._ In our pseudotime plot, cells found in the heterogeneous p1 subset directly connected to the p3 population, which spread widely on the development tree. This “synthetic” developmental process had two branched ends: one was a tight cluster encompassing a majority of quiescent T_RM_s; the other was enriched by highly differentiated p2 T_EM_s and active T_RM_s (Figures 4A and 4B). This suggested that by stemming from T_Eff_s (p3), T_EM_s and T_RM_s may share a similar development process until they reach their final branching point.

In both the transcriptome and regulon analyses, we noticed that the distinction between quiescent and active T_RM_s in the tumor was less profound than that in the distant mammary gland mucosa (Figure 3B–3D). This blurred distinction was also reflected by the differential expression of ribosome-related genes (Figure 3E), suggesting that quiescent T_RM_s in the tumor are relatively “activated” in comparison to their counterparts in the distant mammary gland. To test the impact of different tissue microenvironments and to explore the lineage progression paths leading to distant T_RM_ development, we added T_RM_s collected from the distant mammary gland to the Monocle2 algorithm. Choosing the same proliferating p3 subset as the starting point, we made three major observations (Figures 4C and 4D): (1) except for some active distant T_RM_s that co-localized with active tumor T_RM_s, the majority of T_Eff/EM_ and T_RM_ cells were separated by their tissue of origin, with the mucosal T_RM_ subsets occupying a different space on the contour plot from the tumor memory T cell subsets. It was unexpected to observe that the impact of the tumor or distant mucosa location could overshadow the intrinsic transcriptomic differences between T_Eff/EM_s and T_RM_s. (2) A few tumor T_Eff/EM_ p1 cells crossed the tissue “boundary” to overlap with distant mucosa T_RM_s. This revealed that there are T_Eff/EM_ cells with a transcriptome that mimics distant T_RM_s, although their surfaces are absent of CD103 expression and they are still located in the tumor. (3) A transitioning population of tumor T_Eff/EM_ cells was located at a critical branching point. These precursor T_Eff/EM_ cells largely belong to p1 and p4 subsets and leave this branching point with lineage decisions to become tumor T_RM_s or distant T_RM_s.

We projected the transcriptomic transition of individual T cell subsets approaching and leaving this branching onto a heatmap, which allowed us to discover signature gene features that make up this branching point (Figure 4E). A cluster of 13 genes were identified; 11 of 13 of these genes were signature genes identified in the T helper 17 cell (Th17) lineage, including the effector molecule *Il17f* and the master transcription factor *Rorc* (Chang and Dong, 2009; Ciofani et al., 2012; Gobert et al., 2018; Hu et al., 2013; Kim and Lee, 2015; Li et al., 2009; Skepner et al., 2014; Su et al., 2016; Tu et al., 2018; Zhou et al., 2016). These Tc17-like cells may serve as intermediate progenitors to separate T_RM_s located in the tumor or distant mammary gland mucosa.

To exemplify the differences between tumor or distant T_RM_s, we selected phenotypic genes that specify distinct T cell stages and portrayed their expression on the pseudotime plot. Genes associated with T_Eff/EM_s such as *Lgals3*, *GzmB*, and *Maf* were preferentially expressed in both T_Eff/EM_s and T_RM_s within the tumor environment. Reciprocally, genes associated with T_CM_s such as *Sell* and *Lef1* or *Wnt10a*, which induces anabolic T cell metabolism (Terauchi et al., 2009) and is differentially expressed in long-lived periphery T_CM_s (Miron et al., 2018), were robustly expressed in distant T_RM_s (Figure 4F). Notably, when the top 200 genes differentially expressed in the tumor and distant mucosa T_RM_s were subjected to Gene Ontology analysis, we found that, as we saw in T_CM_s, a large set of genes comprising the structural content of the ribosome, was highly elevated in the distant T_RM_ population (Figure 4G). In contrast, genes involved in cytokine and chemokine signaling were upregulated in the tumor T_RM_ population, highlighting the inflammatory nature of this population. We concluded that the transcriptional program of tumor T_RM_s was adapted to the inflammatory nature of the tumor microenvironment. Meanwhile, in the reservoir of the tumor-free mammary mucosa, distant T_RM_s developed into a quiescent T_CM_-like phenotype to favor their long-term survival.

### CXCR6 expression defines a unique subpopulation of T_Eff/EM_s

**Figure 5.**
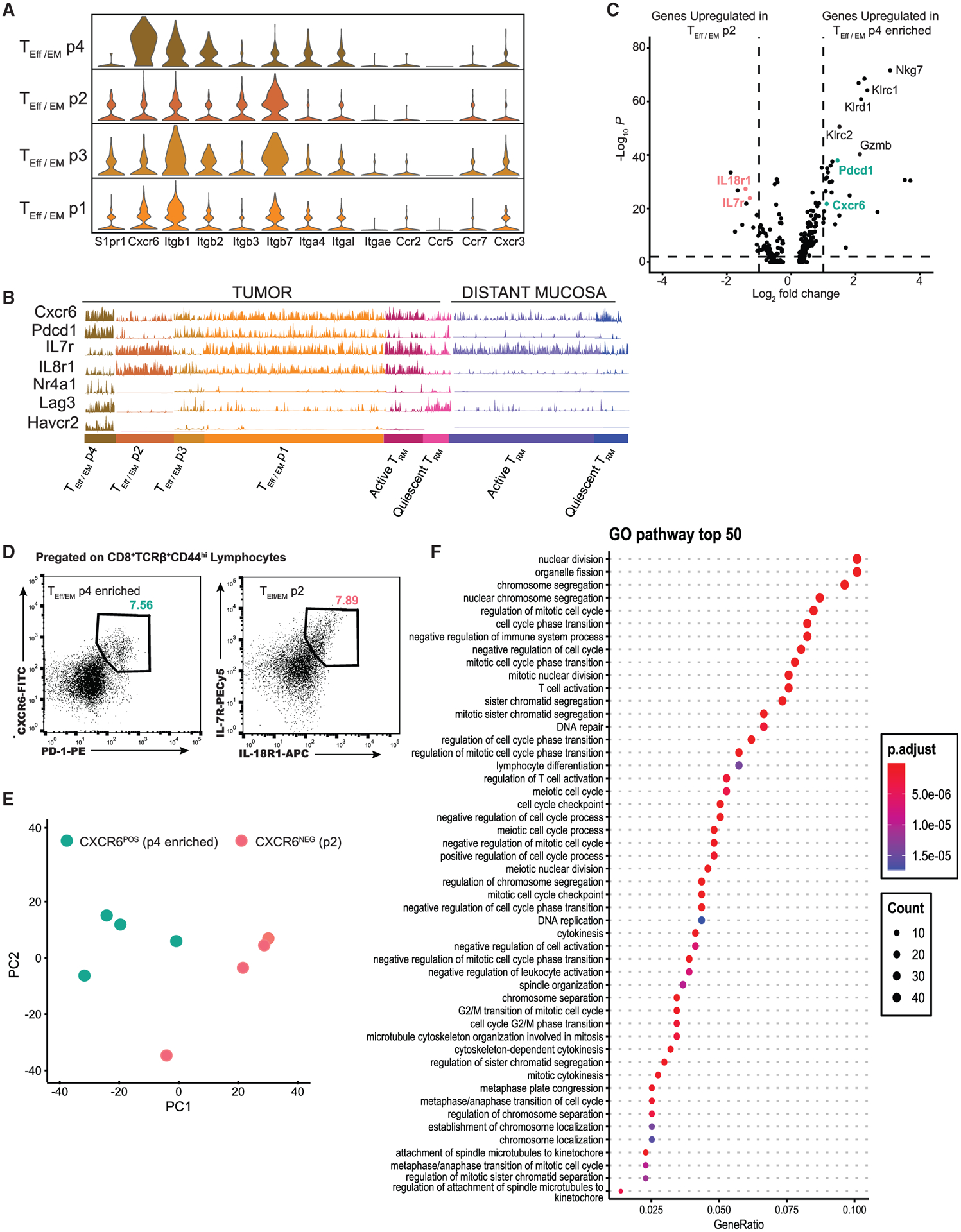
CXCR6 defines a unique subpopulation of T_Eff/EM_s (A) GeneTrac analysis shows expression levels of chemokine receptors and integrins among all tumor T_EFF__/__TEM_ subsets. *Cxcr6* shows the highest expression among the T_Eff/EM_ p4 population and correlates with the lowest *S1PR1* expression. (B) Elevated levels of *Cxcr6* in T_Eff/EM_ p4 are associated with enhanced *Pdcd1* expression, in opposition to *Il7r* and *Il18r1* expression, which are expressed in T_Eff/EM_ p2. Classical exhaustion markers *Nr4a1*, *Lag3*, and *Havcr2* were upregulated in the tumor T_Eff/EM_ p4 population. (C) Volcano plot of gene expression between tumor T_Eff/EM_ p2 (left) and T_Eff/EM_ p4 (right) shows that T cell activation markers *Gzmb*, *Klrc1/2*, and *Klrd1* are upregulated in T_EFF/TEM_ p4, indicating it is effector like. (D) Flow cytometry staining on tumor T cells shows protein expression of the 2 T_Eff/EM_ precursor populations, which were sorted for RNA-seq analysis. (E) PCA analysis confirms transcriptional separation of these 2 populations. (F) Gene Ontology analysis shows that the CXCR6^+^ population enriches many pathways involving the cell cycle, indicating that this pathway is phenotypically exhausted but highly active.

We moved to identify the intratumor precursor of distant T_RM_s within the heterogeneous T_Eff/EM_ pool. We reasoned that these precursor cells should have a distinct chemokine sensing and extracellular matrix rolling profile to facilitate tumor egress. We compiled a list of chemokine receptors and integrins and evaluated their expression. We chose *S1pr1*, a well-known hallmark for T cell tissue egress (Cyster and Schwab, 2012), as a benchmark. Among all of the T_Eff/EM_ populations, *S1pr1* expression was silenced in T_Eff/EM_ p4, suggesting that this may be a population that lacks the potential to egress. In p4, compared to other chemokine receptors, *Cxcr6* was highly expressed; compared to the other 3 populations, p4 was the only population that preferentially upregulated *Cxcr6* (Figure 5A).

The elevated *Cxcr6* expression in p4 was associated with enhanced *Pdcd1* and reduced *IL7r* expression, in direct opposition to p2 (Figure 5B). We virtually sorted out these two populations of cells and compared their gene expression at the transcriptomic level (Figure 5C). The differential expression of *Nkg7* and *Klrc/d* family members suggested that these are highly active effector T cells, as seen in the high expression of effector molecules such as *Gzmb*. However, for T cells in T_Eff/EM_ p2, we found that the upregulation of *IL7r* was associated with *IL18r1* (Figure 5C). For tumor-infiltrating T cells isolated from non-small cell lung cancer samples, IL-18R marks a functional Tbe-t^+^Eomes^+^ T_EM_ population (Timperi et al., 2017).

Taking the co-expression of CXCR6 and PD-1 or IL-7R and IL-18R1 as new stage-specific markers, using mice with established 4T1 tumors, we sorted CD44^hi^CD103^−^CXCR6^+^PD-1^+^T_Eff/EM_ cells, which were highly enriched with the p4 subset (described above) and CD103^−^IL7R^+^IL18R1^+^T_Eff/EM_ cells, which made up the p1 and p2 subsets (Figure 5D) to validate our findings through RNA-seq. Bulk RNA-seq analysis validated that these two populations have distinct transcriptomic profiles (Figure 5E). We directly compared the levels of CXCR6^+^ on tumor T_RM_s versus T_Eff/EM_s and found that overall, more T_Eff/EM_ cells expressed CXCR6^+^ (Figures S5A and S5B). Flow cytometry analysis validated that CXCR6-expressing T_Eff/EM_ cells were enriched in p4. Within this subpopulation, at the individual cell level, the surface expression of CXCR6 was indistinguishable from that of T_RM_s (Figures S5C and S5D) (Takamura et al., 2019; Wein et al., 2018). Gene Ontology pathway analysis overwhelmingly showed that the CXCR6^+^ subset of T_Eff/EM_s was actively in the cell cycle, indicating that this population was proliferative (Figure 5F). This analysis illustrated that these CXCR6^+^ effector cells were quite unique: on the one hand, they could be labeled as terminally exhausted cells (Wherry et al., 2007) based on their elevated expression of *Pdcd1*, *Nr4a1* (Liu et al., 2019), *Lag3*, and *Havcr2* (Tim-3); on the other hand, the transcriptional program in these cells overrode all of these possible inhibitions and maintained these cells in the cell cycle with a robust cytolytic program.

### CXCR6^−^ T_Eff/EM_s are precursors for distant T_RM_ formation

**Figure 6.**
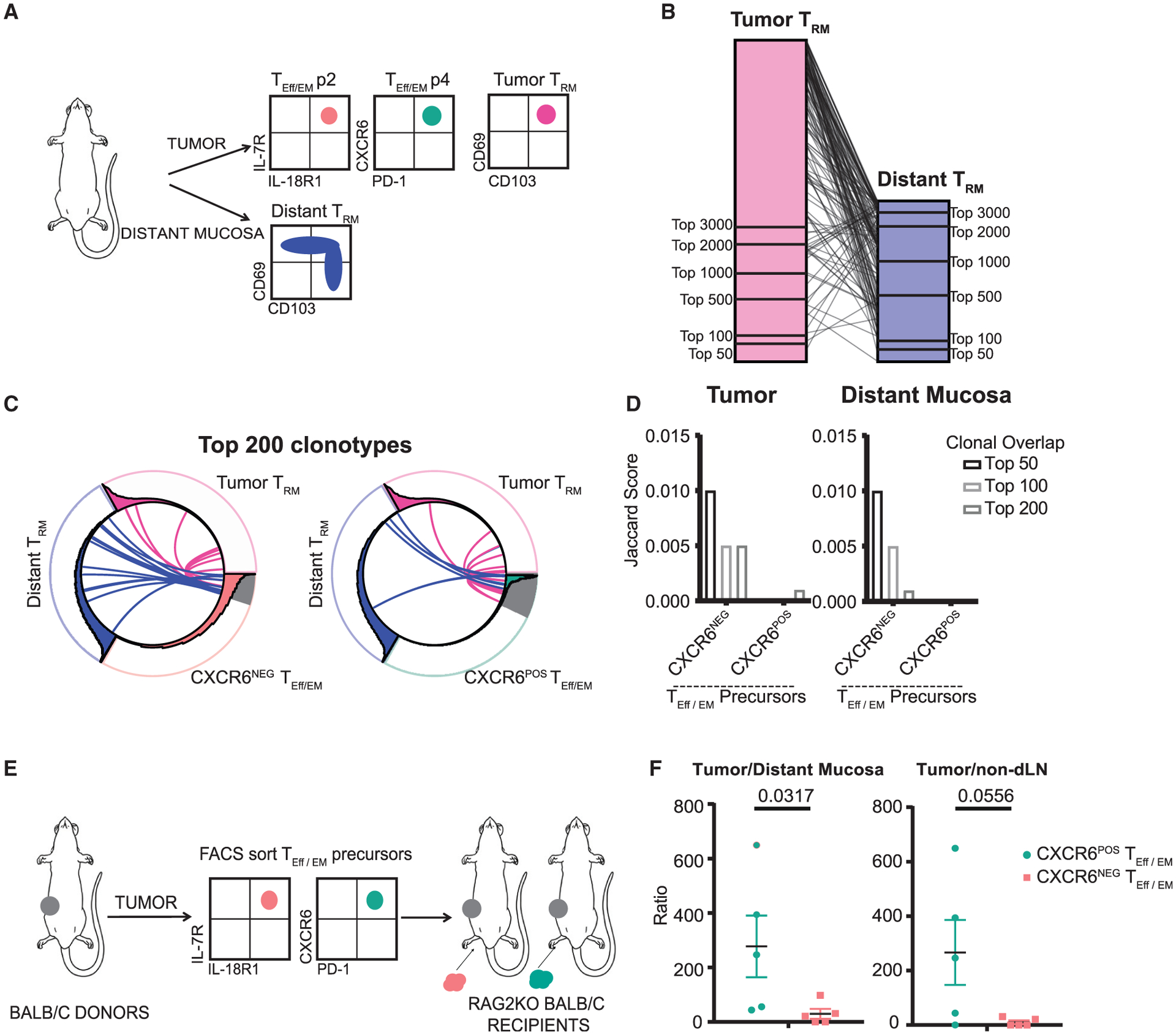
CXCR6^−^ T_Eff/EM_s are the precursor of distant T_RM_s (A) Experimental design of sample collection for TCR-β repertoire sequencing. (B) Repertoire analysis shows substantial overlap between high and low frequency TCRs between tumor and distant T_RM_s. (C and D) Repertoire analysis shows that both CXCR6^NEG^ and CXCR6^POS^ subsets share TCRs with tumor T_RM_s; only the highly expanded clones in the CXCR6^NEG^ population contributed to distant T_RM_ formation. (E) Experimental design for T_Eff/EM_ precursor transfer. (F) Preference of CXCR6^+^ T_Eff/EM_ precursor cells to stay in the tumor while CXCR6^−^ T_Eff/EM_ precursors egress to the distant mucosa. Data are represented as a ratio of recovered cells in the tumor divided by the recovered cells in the distant mucosa or LN. Mann-Whitney test, p < 0.05. Bars represent means ± SEMs and symbols represent individual mice. Data are pooled over 3 independent experiments.

Since CXCR6 and S1PR1 were reciprocally expressed in T_Eff/EM_ cells, we performed TCR-β repertoire sequencing with the purified T_Eff/EM_ subpopulations to trace the lineage relationship between distinct intratumor T_Eff/EM_ populations and distant T_RM_s. We sorted tumor CD44^hi^CD103^−^CXCR6^+^PD-1^+^ T_Eff/EM_ cells and CD103^−^IL7R^+^IL18R1^+^ T_Eff/EM_ cells, as well as tumor and distant T_RM_s (CD103^+^CD69^+^) for repertoire analysis and clonotype lineage tracing (Figure 6A; Table S3). We compared the repertoire overlap between the purified tumor T_RM_s and distant T_RM_s. As previously found in the bulk tissue repertoire analysis (Figure 1I), both high- and low-frequency T_RM_ clonotypes in these two compartments were shared (Figure 6B), supporting that tumor and distant T_RM_s arose from a common precursor population. In addition, we found that both CXCR6^−^IL7R^+^IL18R1^+^ and CXCR6^+^PD-1^+^ subsets shared TCR clonotypes with tumor T_RM_s (Figures 6C and 6D). However, for those highly expanded T_Eff/EM_ clones, only the CXCR6^−^ subset contributed to the formation of distant T_RM_s, while CXCR6^+^ clonotypes barely overlapped with distant T_RM_s. This suggests that CXCR6 may serve as a retention signal to keep T_RM_ precursors in the tumor.

To validate this tumor-retention mechanism, we sorted CXCR6^+^PD-1^+^ and IL7R^+^IL18R1^+^ precursor populations from the tumors of CD45.1^+^ congenically marked BALB/c mice and intratumorally transferred equal numbers (15,000–25,000) into a 4T1 tumor growing in the mammary tissue of Rag2KO BALB/c mice (Figure 6E). Two weeks after transfer, we recovered transferred T_Eff/EM_ precursor cells from the tumor, distant mucosa, and non-draining inguinal lymph node for fluorescence-activated cell sorting (FACS) analysis. We calculated the ratio of cells recovered in the tumor versus the distant mucosa as the readout to minimize experimental variations. The recovered ratios of cells in the tumor versus non-draining lymph node were included as reference. We found that in comparison to CXCR6^−^ cells, the CXCR6^+^ T_Eff/EM_ cells remained in the tumor, validating the preference of the T_Eff/EM_ p4 population to stay in the tumor to become tumor T_RM_s (Figure 6F).

### Breaking CXCR6-mediated retention enhances protection against distant tumor metastasis

**Figure 7.**
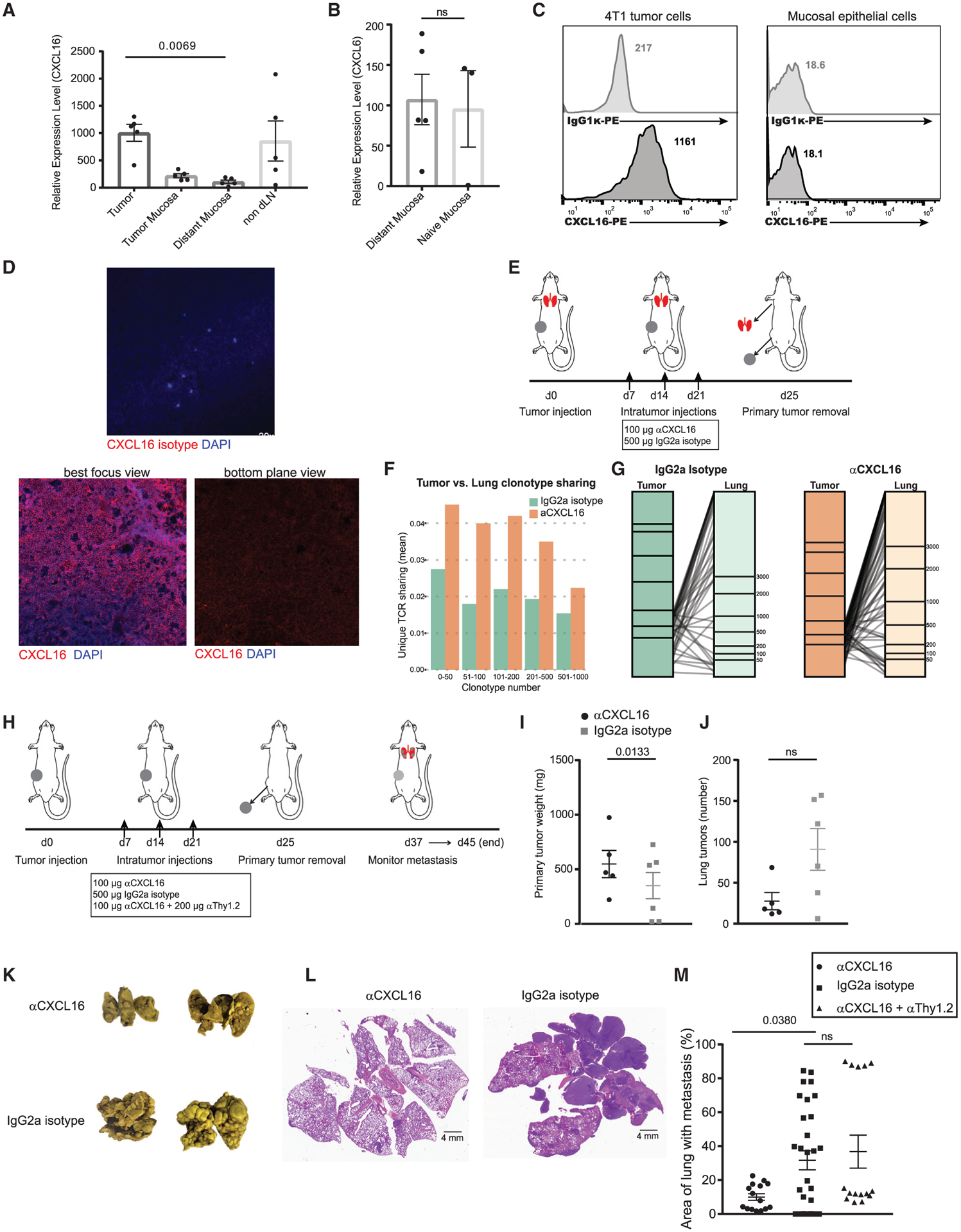
Breaking CXCL16/CXCR6 retention enhances protection against distant lung tumor metastasis (A) qPCR for the CXCR6 ligand *Cxcl16* shows highest expression in the tumor. (B) *Cxcl16* mRNA is similar in the distant mucosa from tumor-bearing mice compared to the mucosa from tumor-naive mice. Two-way ANOVA with Kruskal-Wallis test, p < 0.005. Bars represent means ± SEMs and symbols represent individual mice. (C) Flow cytometry validates CXCL16 expression on the surface of 4T1 tumor cells but not mucosal epithelial cells. (D) Confocal microscopy characterizes CXCL16 expression within the 4T1 primary tumor. (E) Experimental design for sample collection in the presence or absence of anti-CXCL16 blocking. (F and G) Global TCR-β repertoire sequencing analysis compares clonotype sharing between the tumor and the pre-metastatic lung in the presence or absence of anti-CXCL16 blocking. These data are represented in all frequency categories (F) and by zooming into the top 200 high frequency clones (G). (H) Experimental procedure to monitor spontaneous lung metastasis after surgical removal of the primary tumor in the presence or absence of anti-CXCL16 treatment. (I) Analysis of the primary tumors. Bars represent means ± SEMs and symbols represent individual mice. Paired t test, p < 0.05. (J) Analysis of the number of metastatic tumor nodules. (K) Representative images of metastatic tumor lungs. (L) Representative H&E staining of anti-CXCL16 and isotype-treated lungs showing the area of the lung harboring tumor metastases (dark purple). (M) Quantification of metastatic tumor occupancy in the lung, performed by a third party in a 1-sided blinded manner. Data were measured by diving the area of tumor metastases by total lung area and represented as a percentage. Paired t test, p < 0.05. Bars represent means ± SEMs and symbols represent individual lung tissue sections. Data represent 2 individual experiments combined.

Since CXCR6 is the receptor that chemoattracts T_Eff/EM_ cells in the tumor, we predicted that its sole ligand, CXCL16, would be expressed in the tumor tissue. We performed qPCR analysis for *Cxcl16* mRNA with tumor, tumor-adjacent mucosa, distant mucosa, and non-draining lymph node tissues isolated from 4T1 tumor-bearing mice, while the mammary gland mucosa of tumor-naive mice served as a control. Compared to that in mucosal tissues, *Cxcl16* expression in the tumor was significantly stronger (Figure 7A). Furthermore, the relative *Cxcl16* expression level between the distant mucosa from tumor-bearing mice was comparable to that in the tumor-naive mucosa (Figure 7B). Flow cytometry staining confirmed that 4T1 tumor cells can be a direct source of CXCL16 production (Figure 7C). This was further validated by confocal microscopy—within the 4T1 tumor tissues, CXCL16 was almost universally expressed on the surface of 4T1 tumor cells (Figure 7D, best focus view). In addition, produced CXCL16 proteins were deposited on the surrounding extracellular matrix (Figure 7D, bottom plane view). These data indicated that the 4T1 tumor microenvironment can strongly attract CXCR6^+^ T cells to stay.

We speculated that tumor-produced CXCL16 is a key retention molecule used to curb the residency of tumor-specific T cells in distant tissues. Considering the sentinel functions of T_RM_s, this retention could be a mechanism exploited by tumors to dampen immunity in distant tissues and facilitate the engraftment of metastases. To test this, at days 7, 14, and 21 after primary tumor inoculation, we intratumorally injected a CXCL16 antibody to neutralize CXCR6 binding (Figure 7E). At day 25, we surgically removed the primary tumor and performed TCR-β repertoire sequencing to characterize the difference in T cell infiltration in the pre-metastatic lung tissue following CXCL16 antibody blocking. The clonotype sharing was increased across T cells in all frequency categories (Figure 7F), and, zooming in on the high-frequency clones, which were likely to be enriched by expanding tumor-specific T_Eff/EM_ cells, the increase was also obvious (Figure 7G). This suggested that breaking CXCR6-mediated retention in the tumor resulted in more T cells egressing to the distant lung tissue.

We next evaluated whether promoting T cell infiltration to the lung could result in enhanced protection against tumor metastasis. Using the same experimental scheme detailed above, we monitored the animals for 2–3 weeks following the removal of the primary tumor (Figure 7H). Anti-CXCL16 treatment caused a moderate difference in the weight of the primary tumors (Figure 7I); at the humane endpoint of individual mice, we assessed the spontaneous 4T1 metastases in the lung. We counted the metastases on the surface of the lung and found that the number of tumor nodules was not statistically different between control IgG and anti-CXCL16 treated mice (Figure 7J). However, we observed striking differences in metastatic tumor burden (Figures 7K and 7L). To prove that this protective mechanism was T cell intrinsic, we depleted T cells with an anti-Thy1.2 antibody, while co-administering mice with anti-CXCL16 (Figure 7H). The anti-CXCL16- enhanced protection was lost when T cells were depleted (Figure 7M). This suggested that breaking CXCL16-CXCR6-mediated T cell retention in primary breast tumors fortifies antimetastatic immunity in the lung.

## DISCUSSION

In an orthotopic breast cancer model, we found that T_RM_s developed in remote mammary gland tissues at early stages of tumorigenesis. TCR repertoire sequencing data revealed that clonotypically, these T_RM_s were generated in the pre-metastatic stage from the same precursor cells that developed T_EM_s and T_RM_s within the tumor (Figure 6B), as well as T_RM_s in the tumor-adjacent mucosal tissue (Figure 1I). Accordingly, dominant TCR clones in the mammary mucosa were shared among individual tumor-bearing mice. These TCRs were also shared between the tumor and tumor-distant mucosa but were distinct from mammary gland T_RM_s in tumor-naive mice. scRNA-seq showed that a few tumor T_EM_s shared a similar transcriptome with active T_RM_s, although they were sorted based on their classical surface markers. The transcriptomic similarity between tumor T_EM_s and T_RM_s echoed the epigenetic similarity recently identified between these two populations in an LCMV infection model, especially for genomic loci that have the most dynamic changes through naive to memory differentiation (Fonseca et al., 2020). This transcriptomic similarity was reinforced by our pseudotime analysis. During the synthetic developmental process, the transcriptomic transition that generated terminally differentiated T_EM_s was highly similar to the one begetting active T_RM_s. Pseudotime analysis further showed that although they were sorted from tumors, a few CD44^hi^CD62L^−^CD103^−^ cells had already developed a transcriptome resembling that of CD103^+^ T_RM_s purified from the distant mucosa. These results together suggested that distant mucosa T_RM_s were generated from T_Eff/EM_ precursors that developed within the tumor. Before gaining their tumor resident credentials, these precursors were able to egress. After they circulated to and infiltrated the distant mammary gland, they found their necessary niche in the local tissue environment, which harbored and supported them to finish their final differentiation to become T_RM_s in the remote tissue.

The generation of T_RM_s in remote tissues is a protective mechanism against metastasis. Before metastatic tumor cell invasion, T_RM_s can establish their defensive perimeters in distant tissues. Distant T_RM_s function as perfect sentinels. On the one hand, they are derived from T_Eff/EM_ precursors and have tumor antigen specificity, allowing them to detect tumor cells upon their arrival; on the other hand, as memory cells residing in the tissue, they can be reactivated quickly to expedite immune responses locally. These features are especially important for malignancies such as breast cancer, for which the 5-year survival rate in the US is 99% if only localized tumors are found; this rate drops to 27% if there are distant metastases (National Cancer Institute Surveillance, Epidemiology, and End Results [NCI SEER] program). While the importance of T_RM_s in various types of epithelial-associated cancers has recently emerged (Amsen et al., 2018), their exact antitumor roles are not characterized. Our clinical study on early-diagnosed patients with local gastric cancer showed that their 4-year prognosis was associated with the clonal diversity of T_RM_s in the tumor-adjacent mucosa rather than the diversity of T cells in the tumor or peripheral blood. The majority of the mortality in this cohort was the result of stomach cancer relapse or liver or peritoneum metastasis (Jia et al., 2015). Although the distant mucosal tissues of those patients could not be surveyed, based on the repertoire similarity between the tumor-adjacent and tumor-distant mucosa, we speculated that the correlation between prognosis and T_RM_ diversity may reflect the unique immunosurveillance function of extratumoral T_RM_s. Their protective functions go beyond the primary tumor and are specialized against tumor recurrence.

To approach the retention mechanism separating T cells that remain in the tumor versus those that leave to travel to remote tissues, we identified CXCR6 as a key player. CXCR6 was proposed to be the chemokine receptor driving flu-specific T_RM_s to reside in the airway epithelium, while CXCR6^−^ T_RM_s stayed in the interstitium (Wein et al., 2018). In the same influenza infection model, fully differentiated CXCR6^+^ T_RM_s in the interstitium replenished the airway compartment, not circulating T_EM_s (Ely et al., 2006; Takamura et al., 2019; Zammit et al., 2006). Both studies clearly demonstrated that airway CXCL16 production and T_RM_ CXCR6 expression were necessary for airway T_RM_s to form. The present study revealed a similar chemoattraction mechanism trapping effector T cells in the tumor. The heightened expression of CXCL16 by tumor cells is a common characteristic found in tissue from gastric (Xing et al., 2012) and colon carcinoma patients (Hojo et al., 2007), as well as in various mammary carcinoma cell lines (Meijer et al., 2011). This differential expression was also observed in the 4T1 tumor versus the distant mammary gland mucosa or the mucosa from tumor-naive mice. We showed that transferred CXCR6^+^ tumor T_Eff/EM_s were more likely to be trapped in the recipient tumor than their CXCR6^−^ counterparts. Furthermore, in a snapshot of TCR repertoire analysis, more tumor CXCR6^−^ T_Eff/EM_ TCRs overlapped with T_RM_s isolated from the distant mucosa than CXCR6^+^ T_Eff/EM_s in the tumor. We reasoned that in this 4T1 model, tumor cells served as the source of CXCL16 to retain tumor-infiltrating T_Eff/EM_ cells. Our repertoire analysis also showed that TCRs from CXCR6^−^ and CXCR6^+^ subsets had significant overlap. Whether CXCR6 expression is stochastic or how it is regulated in tumor T_Eff/EM_ cells is still not clear. However, our scRNA-seq and FACS data demonstrated a strong correlation between CXCR6 and PD-1 expression, which suggests that it could be related to tumor antigen stimulation.

Our study also showed that T_RM_s, especially T_RM_s in the distant mammary gland, divided into two major populations based on their ribosome-associated gene expression. The elevated expression of these genes was closely related to quiescent features of their transcriptome. This association has been revealed in CD8^+^ memory T cell development during LCMV infection. In this model, both ribosomal protein mRNAs and overall translation activities are drastically decreased when T cells enter the phase of terminal effector differentiation. Ribosomal protein mRNA expression in T_Eff_s was lower than that in memory precursor (T_MP_) cells, and their expression in T_MP_s was lower than in quiescent naive T cells (Araki et al., 2017). Furthermore, suppressed ribosomal protein mRNA expression depended on antigen stimulation and mammalian target of rapamycin complex 1 (mTORC1) activity. The mTORC1 inhibitor rapamycin was shown to promote long-lived T_CM_ formation (Araki et al., 2010). We speculated that this mechanism could be directly applied to T_RM_s. Like LCMV clone 13-induced exhaustion, chronic tumor antigen stimulation may suppress ribosomal protein mRNA expression. Consequently, T_RM_s found in the distant mucosa are isolated from tumor antigens and thus returned to a quiescent stage to cope with their longevity. However, this model cannot fully explain how T_CM_s found in the tumor microenvironment maintain their robust ribosomal protein mRNA expression or the evolutionary factors that keep these mRNA levels abundant in quiescent cells, both of which are subjects for future study.

## STAR★METHODS

### RESOURCE AVAILABILITY

#### Lead contact

Further information and requests for resources and reagents should be directed to and will be fulfilled by the Lead Contact, Qi-Jing Li (Qi-Jing.Li@Duke.edu).

#### Materials availability

This study did not generate any unique reagents.

#### Data and code availability

The TCR repertoire and single-cell RNA sequencing datasets generated during this study is available at: https://doi.org/10.17632/3f4rsk96kf.3, https://data.mendeley.com/datasets/3f4rsk96kf/3.

### EXPERIMENTAL MODEL AND SUBJECT DETAILS

#### Animals

BALB/c mice (BALB/cJ) and congenically marked CD45.1^+^ BALB/c mice (CByJ.SJL(B6)-*Ptprc*^*a*^/J0) were purchased from The Jackson Laboratory. Rag2-KO BALB/c mice (C.129S6(B6)-*Rag2*^*tm1Fwa*^ N12) were purchased from Taconic. All mice were housed under pathogen-free conditions and only female mice were used between 6–10 weeks for experimental procedures. Littermates of the same sex were randomly assigned to experimental groups. All mice were used in accordance with Institutional Animal Care and Use Committee guidelines at Duke University.

#### Cell lines

The 4T1 mammary carcinoma cell line was a gift from Xiao-Fan Wang (Duke University); the cell line was authenticated prior to their use in experiments. 4T1 tumor cells were grown in coordination with ATCC guidelines: ATCC-formulated-RPMI-1640 Medium (ATCC 30–2001) was used and supplemented with 10% fetal bovine serum. Cells were grown at 37°C with 5% CO_2_ in 100mm cell culture dishes (VWR). Cells were subcultured at 80% confluence at a ratio of 1:6.

### METHOD DETAILS

#### Tumor model and tissue isolation

Only female mice were used in all experiments. The 4T1 mammary carcinoma cell line was a gift from Xiao-Fan Wang (Duke University). Tumor cells were harvested by trypsinization, and cell viability was evaluated by trypan blue exclusion. 100 4T1 cells in 10 μL of serum-free media were orthotopically injected directly into the mammary gland of anesthetized Female BALB/c mice using a micro-syringe with a 26-gauge needle (Hamilton Company, Reno, NV). Tumor progression was monitored closely and tumor growth kinetics were measured. Mice were sacrificed at three weeks post tumor injection for all tumor-cell sorting experiments and their tumors, tumor mammary mucosa, and contralateral (distant) mammary mucosa tissue were harvested. The primary tumor was first removed, followed by dissection of the remaining mucosa tissue surrounding the tumor (tumor mucosa). Care was taken during the dissection process to ensure that the inguinal lymph nodes were removed prior to mammary mucosa tissue harvest. All tissues were mechanically homogenized and filtered over 70 μm nylon mesh filters (VWR) to obtain a single cell suspension for downstream assays. Enzymatic digestion was avoided as to eliminate the possibility that antibody binding sites could be degraded.

#### Tumor T_RM_ characterization

To characterize the developmental time-course of tumor T_RM_s, mice were sacrificed either 2 weeks, 3 weeks or 4 weeks post 4T1 tumor injection. Tumors were harvested and homogenized into a single suspension. Isolated cells were blocked with anti-mouse CD16/CD32 Fc Block (2.4G2) for 10 minutes prior to antibody staining. Cells were stained with antibodies to CD4 (RM4–5), CD8α (53–6.7), TCRβ (H57–597), CD44 (IM7), CD103 (2E7), CD62L (MEL-14) and CD69 (H1.2F3). All data was acquired on a BD FacsCanto flow cytometer (BD Biosciences) and analyzed using FlowJo software (Treestar).

#### TCRβ repertoire sequencing

Tumor, tumor-adjacent mammary mucosa, contralateral mammary mucosa, draining lymph nodes and spleen were isolated from 4T1 tumor bearing mice at 3-weeks post tumor injection and lysed in TRIzol Reagent (Sigma Aldrich). RNA was extracted using the Direct-zol RNA kit (Zymo Research) according to the manufacturer’s instructions. cDNA was synthesized using the qScript Flex cDNA synthesis kit (Quanta Biosciences) with a constant region specific primer (5′-ATCTCTGCTTCT- GATGGCTCA-3′). Multiplex PCR was performed to amplify the CDR3 region of rearranged *TCRB* loci and a set of primers, each specific to a specific TCR Vβ segments, and a reverse primer to the constant region of *TCRB* were used to generate a library of amplicons that cover the entire CDR3 region. PCR products were loaded on agarose gels and bands between 220–240 bp were extracted and purified using the QIAquick Gel Extraction kit (QIAGEN). These purified products were sequenced using the Illumina HiSeq X Ten machine.

#### TCRβ repertoire analysis

Sequence data were analyzed with MiXCR (v3.06) (Bolotin et al., 2015). This software first aligned sequence short reads to reference T cell receptors, then extracted CDR3 sequences and exported TCR clonotypes. The *tcR* package under the R computing environment was used for clonotype summary, repertoire diversity, and similarity analysis. In these analyses, out-of-frame TCR clonotypes were excluded (Nazarov et al., 2015). Figures were plotted using R *ggpubr*.

#### 10x Genomics library preparation and sequencing

Libraries were prepared following the 10x Genomics Single Cell 3′ protocol. Single cells were dissociated, washed and resuspended in a 1x PBS/0.04% BSA solution at a concentration of 1000 cells/μl to remove dead cells and contaminants. A Cellometer (Nexcelom) was used to determine cell viability and cells were normalized to 1 × 10^6^ cells/mL. Cells were then combined with a master mix including reverse transcription reagents. With this, gel beads carrying the Illumina TruSeq Read 1 sequencing primer, a 16bp 10x barcode, a 12bp unique molecular identifier (UMI) and a poly-dT primer were loaded onto the chip, together with oil for the emulsion reaction. Reverse transcription occurs in nanoliter-scale gel beads in emulsion (GEMs) so that all cDNAs within a GEM share a common barcode. After this reverse transcription reaction, the GEMs were broken and full length cDNA purified with Silane Dynabeads and SPRI beads then assayed on an Agilent 4200 TapeStation High Sensitivity D5000 ScreenTape (Santa Clara, CA) for qualitative and quantitative analysis. Illumina P5 and P7 sequences (San Diego, CA), a sample index and TruSeq read 2 primer sequences were added via End Repair, A-tailing, Adaptor Ligation and PCR. Sequences were generated using paired end sequencing on an Illumina sequencing platform at a minimum of 50,000 reads/cell.

#### Single-cell RNA-seq analysis

Raw short reads were demultiplexed, filtering and mapped to mouse genome GRCm38/mm10 using cellranger v2.02. The gene count matrices from cellranger were subjected to quality control, pre-processing and clustering using the R Seurat 2.3.4 package (Butler et al., 2018). Low-quality cells that had less than 200 expressed genes and more than 5% mitochondrial genes were filtered out. Gene counts were scaled to total gene expression and percentage of mitochondrial genes with a scaling factor of 10,000, and then log-transformed. The high dimensional data for each sample were reduced by PCA and t-Distributed Stochastics Neighbor Embedding (*tSNE*). We used the FindCluster function to group clusters in each sample with a resolution of 0.6. Differential expressed genes (DEGs) were identified using the Wilcoxon rank-sum test. The python package *scanpy* v 1.4.1 (Wolf et al., 2018) was used to integrate analysis for all samples following the common procedure. The expression matrix was normalized and log transformed through scanply.pp.log1p function, and high variable genes were chosen by scanpy.pp.highly_variable_genes. SCENIC (Aibar et al., 2017) was used to perform the gene regulatory network (regulon) analysis. This algorithm contained three major steps: 1) find the gene co-expression modules between transcription factors and target genes using GENIE3 (R package); 2) identify co-expression modules between cis-regulatory motif and their target genes using RcisTarget; 3) score each regulon through AUCell to select the top regulons in each sample.

For our Genetrac analysis, which plots the gene expression values in each measured cell across pre-defined groups, we used the *scanpy* toolkit but did not use the option of hierarchical clustering, so the cells within each group were ordered randomly. To infer the lineage development of T_EFF_ / _EM_ to T_CM_, we used Monocle package v2.5.4 (Qiu et al., 2017). The top 2000 significant DEGs were chosen to order genes for trajectory reconstruction using the DDRTree method followed by dimension reduction, cell trajectory inference and pseudo-time measurements, which were computed via reversed graph embedding.

#### Bulk RNA-sequencing

Tumors were harvested from 4T1 tumor bearing mice and homogenized into a single cell suspension. Isolated cells were stained with a LIVE/DEAD Fixable Aqua stain (Thermo Fisher) and blocked with anti-mouse CD16/CD32 Fc Block (2.4G2) for 10 minutes prior to antibody staining. Cells were stained with antibodies to CD8α (53–6.7), TCRβ (H57–597), CD44 (IM7), CD127 (A7R34), IL18R (BG/IL18RA), CD279 (RMPI-30), and CXCR6 (SA051D1) and sorted using the MoFlo Astrios cell sorter. Depending on the experimental yield for 4 replicates, 2.5×10^4^ – 5×10^4^ of each T_EM_ precursor population were sorted and directly lysed in Lysis Buffer. RNA was extracted using the RNAqueous Micro RNA Isolation Kit (Thermo Fisher) according to the manufacturer’s instructions. RNA samples were evaluated for concentration by Qubit (Thermo Fisher) and integrity using an Agilent 2100 Bioanalyzer. Clontech Ultra low libraries were prepared and sequenced using the Illumina HiSeq platform.

#### Bulk RNA-sequencing analysis

High-throughput short reads were trimmed, filtered, mapped, and counted. The sequence quality was assessed using *fastqc* software (Andrews, 2010) and filtered using *fastp* (PMID: 30423086 or Chen et al., 2018). Reads were mapped to mouse reference M23 (Genecode) using *STAR* software (PMID: 23104886 or Dobin et al., 2013). For RNA-seq, read counts per gene were generated using *featureCounts* (PMID: 24227677 or Liao et al., 2014). *DESeq2* was used to perform counts normalization and test for differential expressed genes (Love et al., 2014). Gene ontology (GO) enrichment analysis was computed using ClusterProfiler (Yu et al., 2012).

#### Adoptive transfer of T_EM_ precursor cells

Tumors were harvested from female CD45.1^+^ BALB/c mice bearing 3 week established 4T1 tumors and homogenized into a single cell suspension. Isolated cells were stained with a LIVE/DEAD Fixable Aqua stain (Thermo Fisher) and blocked with anti-mouse CD16/CD32 Fc Block (2.4G2) for 10 minutes prior to antibody staining. Cells were stained with antibodies to CD8α (53–6.7), TCRβ (H57–597), CD44 (IM7), CD127 (A7R34), IL18R (BG/IL18RA), CD279 (RMPI-30), and CXCR6 (SA051D1) and sorted using the MoFlo Astrios cell sorter. 1.5×10^4^ – 2×10^4^ cells of each T_EM_ precursor population were sorted depending on the yield of the individual experiment. These cells were directly transferred orthotopically into the mammary gland of 2-week tumor-bearing Rag2KO BALB/c mice. Two weeks later, animals were sacrificed and stained with antibodies to CD8α (53–6.7), TCRβ (H57–597), CD44 (IM7), CD103 (2E7), CD45.1 (A20) and CD45.2 (104) for flow cytometry analysis on BD FACSCanto flow cytometers (BD Biosciences) and analyzed with FlowJo software (Treestar).

#### CXCL16 qPCR

Tumor, tumor-adjacent mammary mucosa, contralateral mammary mucosa, draining lymph nodes and spleen were isolated from 4T1 tumor bearing mice and lysed in TRIzol Reagent (Sigma Aldrich). RNA was extracted using the Direct-zol RNA kit (Zymo Research) according to the manufacturer’s instructions. After annealing oligo-DT primers, cDNA was synthesized using the qScript Flex cDNA synthesis kit (Quanta Biosciences), according to the manufacturer’s instructions. SYBR-Green based real-time PCR method was used to quantify the relative expression of *Cxcl16* mRNA and three housekeeping genes: *Hprt, Sdha, Ywhaz*.

Cxcl16-F 5′-TGTGGAACTGGTCATGGGAAG-3′
Cxcl16-R 5′-AGCTTTTCCTTGGCTGGAGAG-3′
Hprt-F 5′-AGTGTTGGATACAGGCCAGAC-3′
Hprt-R 5′-TGCGCTCATCTTAGGCTTTGT-3′
Sdha-F 5′-TTATTGCTACTGGGGGCTACGGG-3′
Sdha-R 5′-AGGCAGCCAGCACCGTATATACC-3′
Ywhaz-F 5′- ACGCTCCCTAACCTTGCTTC-3′
Ywhaz-R 5′-ACACACCGAACTGTTGTCGT-3′

#### CXCL16 flow cytometry

4T1 tumor cells were cultured for 24 hours in media containing 20 ng/mL TNFα and 20 ng/ml IFNγ cytokines (Peprotech) in the presence of 1 μM ADAM10 inhibitor to prevent membrane shedding (GI254023X, Sigma). Cells were dissociated with an enzyme-free dissociation buffer and homogenized to a single cell suspension. Mucosal epithelial cells were harvested directly from a tumor naive BALB/c mouse and were mechanically homogenized and filtered over 70 μm nylon mesh filters (VWR) to obtain a single cell suspension. Isolated cells were stained with a LIVE/DEAD Fixable Aqua stain (Thermo Fisher) and blocked with anti-mouse CD16/CD32 Fc Block (2.4G2) for 10 minutes prior to antibody staining. Cells were stained with either CXCL16 antibody (12–81, BD Biosciences) or isotype control (Rat IgG1κ, Biolegend). All data was acquired on a BD FacsCanto flow cytometer (BD Biosciences) and analyzed using FlowJo software (Treestar).

#### Confocal microscopy

Fresh tissue samples from 4T1 tumors were cryosectioned into 20 μm sections followed by immediate fixation in 4% PFA for 20 minutes. Tissue slides were immunostained with a primary antibody against CXCL16 (12–81, BD Biosciences) followed by a Cy 3 Goat Anti-Rat IgG secondary antibody (Jackson ImmunoResearch), along with control slides which did not receive secondary antibody stain. Microscopic images were acquired with a confocal microscope (Carl Zeiss 710 inverted) using a 20x objective. Images were construction with z stacks of images using Zen imaging software (Zeiss).

#### Antibody blocking experiments, lung H&E analysis

BALB/c mice bearing 4T1 tumors were intratumorally injected with either 100 μg anti-CXCL16 (142417, Leinco Technologies), with or without 200 μg anti-Thy1.2 (30H12, BioXCell) or 500 μg IgG2a isotype antibody (2A3, BioXCell) at days 7, 14 and 21 post tumor injection. Primary tumors were surgically removed at day 25 and mice were monitored for their humane endpoint. At this time, lungs were harvested and placed into Bouin’s solution fixative for 48 hours for visualization and quantification of lung tumor nodules. A representative subset of lungs (n = 3) were placed in a 70% ethanol aqueous solution and routine hematoxylin and eosin (H&E) staining was performed. Each sample was sectioned into 5 slices and independently analyzed by three oncologists to evaluate the total area of the lung harboring tumor metastases. The metastatic tumor area and total lung area were first calculated separately, then the tumor area was divided by total lung area; resultant data were represented as a percentage of the area of the lung harboring metastases. All image processing analysis was performed in ImageJ software (NIH).

### QUANTIFICATION AND STATISTICAL ANALYSIS

All statistical analysis was carried out using GraphPad v7.03 and R programming language. Specific statistical methods used for analysis are detailed in the text. A p value of less than 0.05 was set as statistically significant.

## Supplementary Material

1

2

## Figures and Tables

**Table:** KEY RESOURCES TABLE

REAGENT or RESOURCE	SOURCE	IDENTIFIER
Antibodies		
Purified Mouse monoclonal anti-CD16/32	Bio X Cell	Cat# BE0307; RRID:AB_2736987
Mouse monoclonal anti-CD4	Biolegend	Cat# 100510; RRID:AB_312713
Mouse monoclonal anti-CD8α	Biolegend	Cat# 100712; RRID:AB_312751
Mouse monoclonal anti-TCRβ	Biolegend	Cat# 109228; RRID:AB_1575173
Mouse monoclonal anti-TCRβ	Biolegend	Cat# 109206; RRID:AB_313429
Mouse monoclonal anti-CD44	Biolegend	Cat# 103012; RRID:AB_312963
Mouse monoclonal anti-CD103	Biolegend	Cat# 121418; RRID:AB_2128619
Mouse monoclonal anti-CD62L	Biolegend	Cat# 104428; RRID:AB_830799
Mouse monoclonal anti-CD69	Biolegend	Cat# 104512; RRID:AB_493564
Mouse monoclonal anti-CXCR6	Biolegend	Cat# 151108; RRID:AB_2572145
Mouse monoclonal anti-CD279	Biolegend	Cat# 109104; RRID:AB_313421
Mouse monoclonal anti-IL18R1	Biolegend	Cat# 132903; RRID:AB_2123952
Mouse monoclonal anti-CD127	ThermoFisher	Cat# 15–1271-82; RRID:AB_468793
Mouse monoclonal anti-CD45.1	Biolegend	Cat# 110708, RRID:AB_313497
Mouse monoclonal anti-CD45.2	Biolegend	Cat# 109830, RRID:AB_1186098
Mouse monoclonal anti-CXCL16	BD Biosciences	Cat# 566740; RRID:AB_2869842
Rat monoclonal IgG1 κ	Biolegend	Cat# 400408; RRID:AB_326514
Cy3 AffiniPure Goat Anti-Rat IgG	Jackson ImmunoResearch	Cat# 112–165-175; RRID:AB_2338252
Mouse monoclonal CXCL16	Leinco Technologies	Cat# C1430; RRID:AB_2828491
Rat IgG2a isotype control	Bio X Cell	Cat# BE0089
Chemicals, peptides, and recombinant proteins		
TRI Reagent	Sigma	Cat# 93289
Human TNFα (Mouse Monoclonal)	Peprotech	Cat# 500-M26
Human IFNγ (Mouse Monoclonal)	Peprotech	Cat# 500-M90
GI254023X	Sigma	Cat# SML0789
Bouin’s solution	Sigma	Cat# HT10132
Critical commercial assays		
Direct-zol RNA kit	Zymo Research	Cat# R2070
qScript Flex cDNA kit	Quanta bio	Cat# 95049–100
QIAquick Gel Extraction kit	QIAGEN	Cat# 28706
Chromium Single Cell 3’ GEM, Library & Gel Bead Kit v3	Illumina	Cat# PN-1000092
LIVE/DEAD Fixable Aqua Dead Cell Stain Kit	ThermoFisher	Cat# L34957
RNAqueous Micro RNA Isolation Kit	ThermoFisher	Cat# AM1931
Deposited data		
TCR Repertoire Data	This paper	https://data.mendeley.com/datasets/3f4rsk96kf/3
Single-cell RNA-Sequencing Data	This paper	https://data.mendeley.com/datasets/3f4rsk96kf/3
Experimental models: Cell lines		
Mouse: 4T1 cells	ATCC	CRL-2539
Experimental models: Organisms/strains		
Mouse: BALB/cJ	The Jackson Laboratory	JAX: 000651
Mouse: CByJ.SJL(B6)-*Ptprc*^*a/*^J0	The Jackson Laboratory	JAX: 006584
Mouse: C.129S6(B6)-*Rag2*^*tm1Fwa*^ N12	Taconic	Taconic: 601-F
Oligonucleotides		
Primer: TCRbβ constant region: 5’- ATCTCTGCTTCTGATGGCTCA-3’	This paper	N/A
Primer: Cxcl16 Forward: 5’- TGTGGAACTGGTCATGGGAAG-3’	This paper	N/A
Primer: Cxcl16 Reverse: 5’- AGCTTTTCCTTGGCTGGAGAG-3’	This paper	N/A
Software and algorithms		
FlowJo	BD Life Sciences	https://www.flowjo.com/
GraphPad Prism v7.03	GraphPad	https://www.graphpad.com/scientific-software/prism/
Zen Imaging	Zeiss	https://www.zeiss.com/microscopy/us/products/microscope-software/zen.html
ImageJ	Schneider et al., 2012	https://imagej.nih.gov/ij/
MiXCR v3.06	Bolotin et al., 2015	https://mixcr.readthedocs.io/en/master/
tcR	Nazarov et al., 2015	https://github.com/imminfo/tcr
R ggpubr	Wickham, 2016	https://rpkgs.datanovia.com/ggpubr/
R Seurat v2.3.4	Butler et al., 2018	https://github.com/satijalab/seurat/releases
Cellranger v2.02	10x Genomics	https://support.10xgenomics.com/single-cell-gene-expression/software/pipelines/latest/installation
Scanpy v1.4.1	Wolf etal., 2018	https://scanpy.readthedocs.io/en/stable/
SCENIC	Aibar et al., 2017	https://github.com/aertslab/SCENIC
Monocle v2.5.4	Trapnell et al., 2014	http://cole-trapnell-lab.github.io/monocle-release/
fastqc	Andrews, 2010	https://www.bioinformatics.babraham.ac.uk/projects/fastqc/
fastp	Chen et al., 2018	https://github.com/OpenGene/fastp
STAR v2.75	Dobin et al., 2013	https://github.com/alexdobin/STAR
featurecounts	Liao et al., 2014	http://subread.sourceforge.net/
DESeq2	Love et al., 2014	https://bioconductor.org/packages/release/bioc/html/DESeq2.html
ClusterProfiler	Yu etal., 2012	https://bioconductor.org/packages/release/bioc/html/clusterProfiler.html

## Highlights

- T_RM_s in tumors and distant tissues are developed from T_Eff/EM_ precursor cells
- T_RM_s comprise active and quiescent subsets that resemble T_EM_s and T_CM_s
- Tumor-specific T_RM_s reside in distant tissues before tumor metastasis
- CXCL16 in tumor microenvironment traps T_RM_ precursors to facilitate metastasis
