# Supplementary material for: Resident memory T cells in tumor-distant tissues fortify against metastasis formation
Source: Cell Rep. Author manuscript; Available in PMC 2021 Jun 15. (PMC8204287; doi:10.1016/j.celrep.2021.109118)
Supplement: 1 [file NIHMS1703505-supplement-1.pdf]

Cell Reports, Volume *35*

Supplemental information

Resident memory T cells in tumor-distant tissues fortify against metastasis formation

Laura S. Christian, Liuyang Wang, Bryan Lim, Dachuan Deng, Haiyang Wu, Xiao-Fan Wang, and Qi-Jing Li

Resident memory T cells in tumor-distant tissues fortify in metastasis formation

Laura S Christian[1†], Liuyang Wang[2†], Bryan Lim[1], Dachuan Deng[3], Haiyang Wu[3], Xiao-Fan Wang[4], Qi-Jing Li[1*]

Supplemental Information

Supplemtentary Figures 1-5
Supplementary Tables 1-3

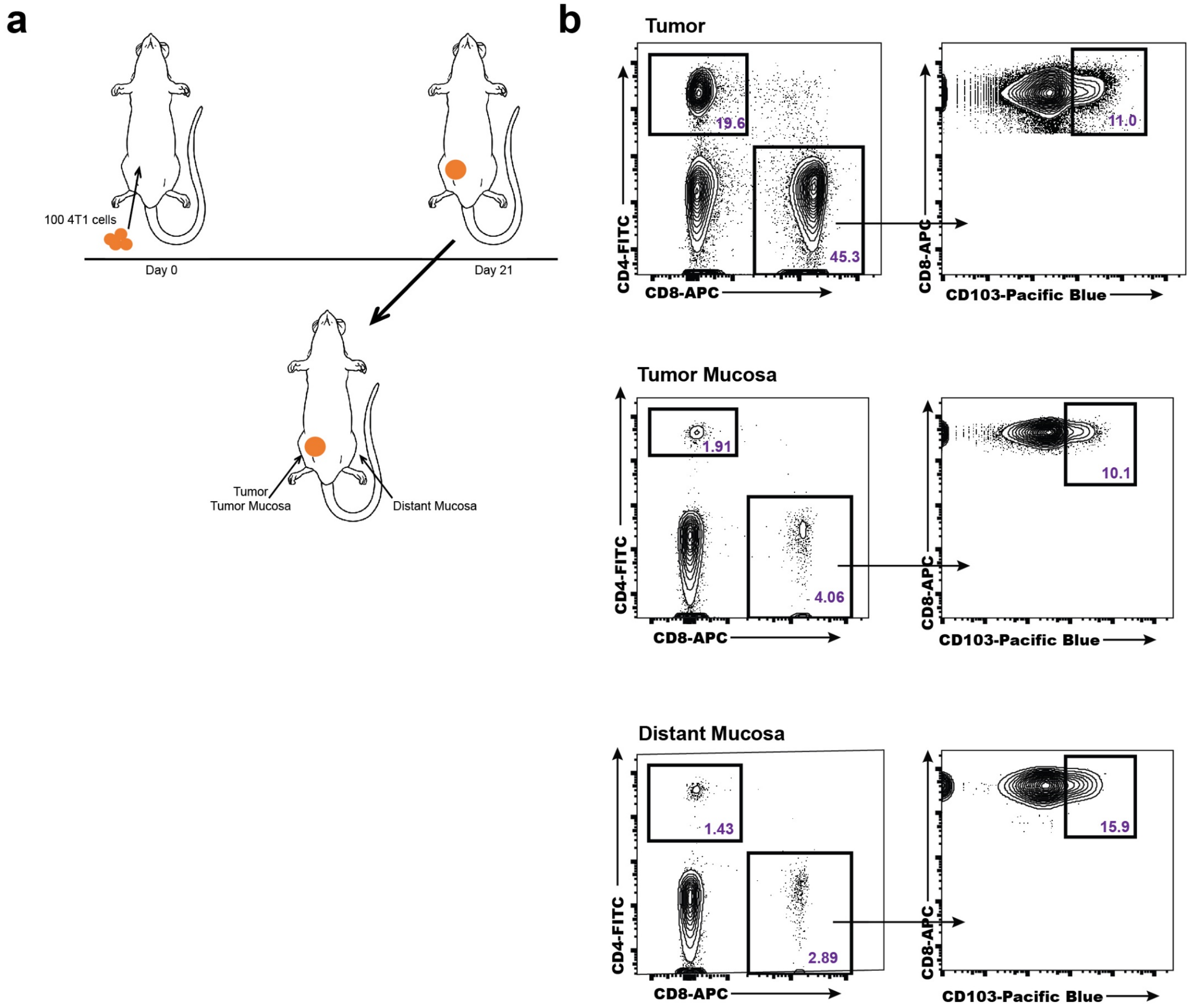

Figure S1. Related to Figure 1: Experimental design for tumor T$_{RM}$ generation and sample collection. a, Experiments using titrating amounts of 4T1 tumor cells revealed that orthotopic injection of 100 4T1 cells into the mammary fat pad of 4T1 mice caused robust tumor formation at 21 days post injection. The diagram shows the location of the tumor, tumor mucosa and distant mucosa, the main sites of sample collection. **b,** Representative flow cytometry plots and gating of T$_{RM}$s in the tumor, tumor mucosa and distant mucosa at d21 post tumor cell injection.

Distant mucosa

Lung

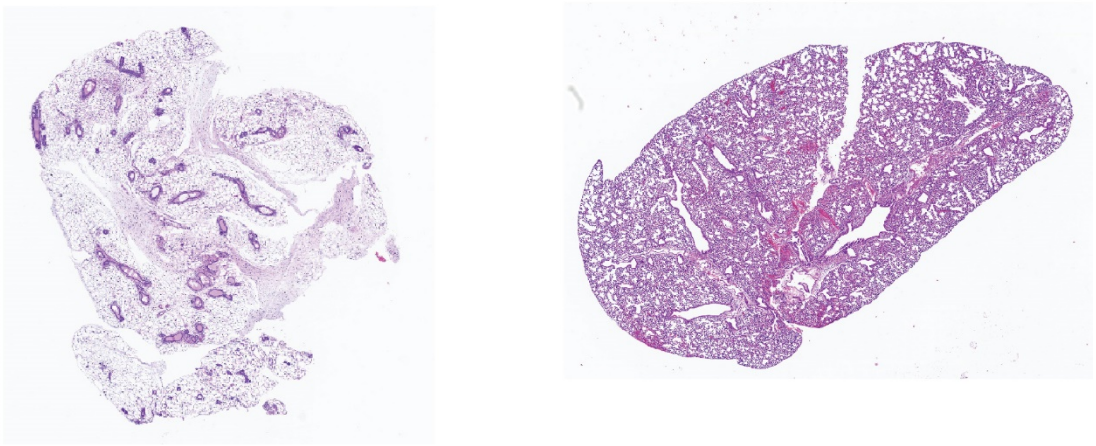

Figure S2. Related to Figure 1: Metastatic tumors are not found in the distant mucosa or lung 3 weeks post tumor implantation. Representative H&E images of distant mucosa or lung harvested from 4T1 tumor-bearing mice 3 weeks post tumor implantation reveals there are no tumor metastases present at this early time-point.

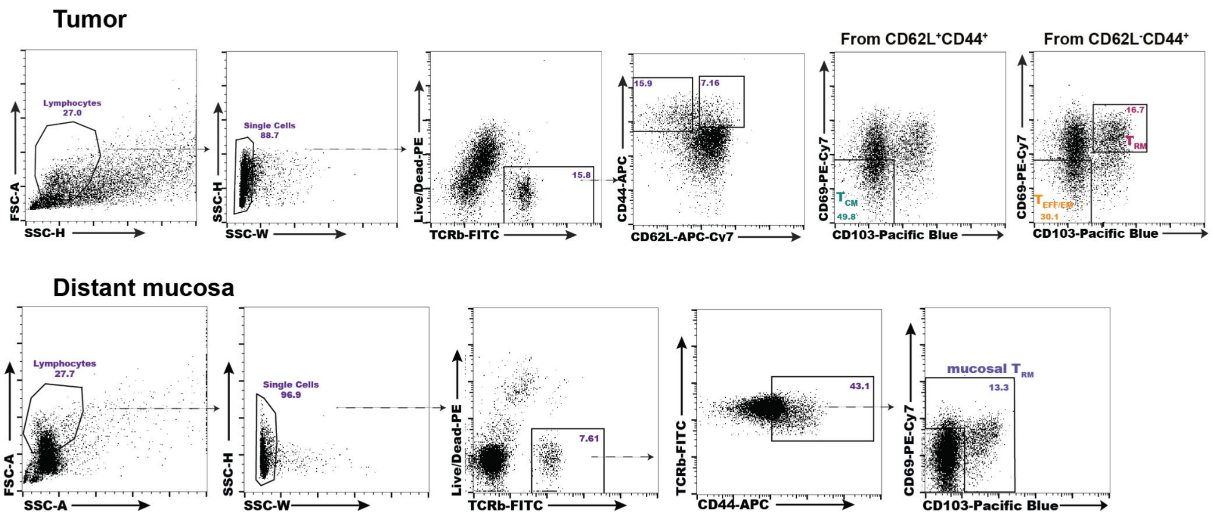

Figure S3. Related to Figure 2: Sorting strategy for single cell RNA-sequencing sample collection. Representative plots showing the flow cytometry sorting strategy for tumor T_{EM}s, T_{CM}s, T_{RM}s and distant mucosa T_{RM}s for single-cell RNA-sequencing sample collection. Tumors were harvested 21 days after tumor injection and cells were stained for various cell surface markers as detailed above.

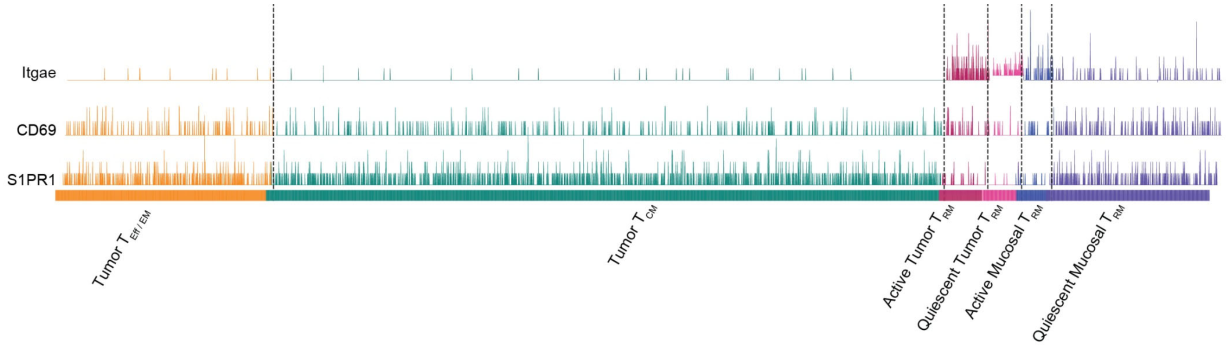

Figure S4. Related to Figure 3: Gene expression of T_{RM} markers confirms heterogeneity of tumor T_{RM}s. GeneTrac analysis of T_{RM} defining marker *Itgae* (CD103) is upregulated only in the T_{RM} populations, concomitant with reduced *S1pr1* expression.

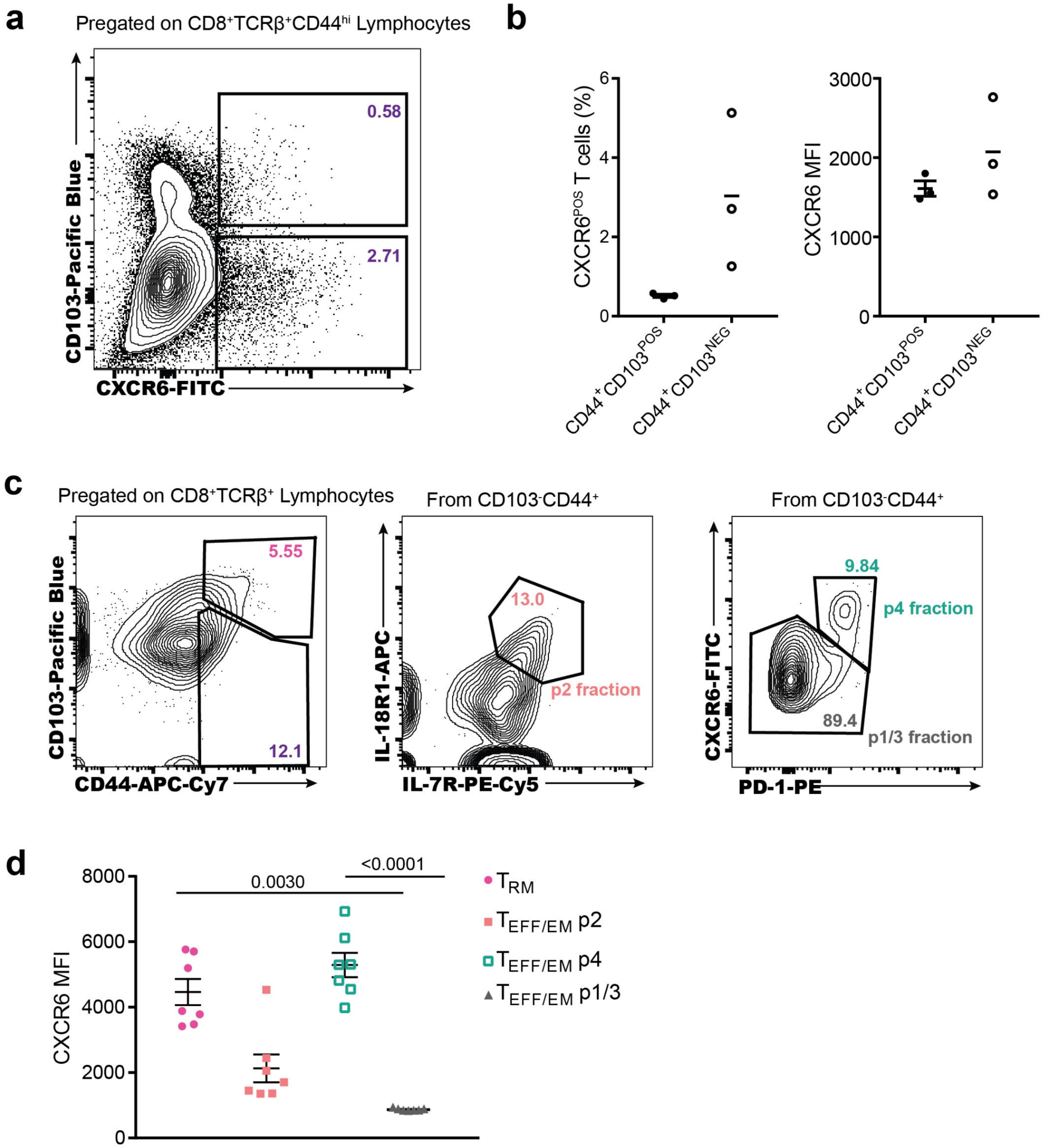

Figure S5. Related to Figure 5: Intra-tumoral T$_{\text{Eff/EM}}$ p4 cells preferentially express CXCR6. a, Representative flow plot on 4T1 tumor shows that intra-tumor T$_{\text{Eff/EM}}$s express more CXCR6 than T$_{\text{RM}}$s. These data are graphed by **b,** the percentage of CD44^{+}CD103$^{\text{POS/NEG}}$ cells expressing CXCR6^{+} and by CXCR6 MFI. **c,** Representative flow plots of 4T1 tumor shows that the intra-tumor T$_{\text{Eff/EM}}$ p4 population expresses more CXCR6 than T$_{\text{Eff/EM}}$ p2. These data are graphed by **d,** CXCR6 MFI. Symbols represent individual mice and error bars represent mean ± s.e.m. $p<0.05$, Kruskal-Wallis test with multiple comparisons.

Supplementary Table 1: QC analysis of TCRβ repertoire sequencing samples related to Figure 1.

Sample ID	Total RNA	Total Mapped Reads	Unique TCR Clonotypes	Mean Read Per TCR
Tumor 1	500ng	84329	2626	32.11
Tumor 2	500ng	37368	2236	16.71
Tumor 3	500ng	82313	24636	3.34
Tumor Mucosa 1	500ng	16869	91	185.37
Tumor Mucosa 2	500ng	36775	1244	29.56
Tumor Mucosa 3	500ng	15249	70	217.84
Distant Mucosa 1	500ng	27106	88	308.02
Distant Mucosa 2	500ng	20882	135	154.68
Distant Mucosa 3	500ng	12359	191	64.71
WT Mucosa 1	300ng	138109	1299	106.32
WT Mucosa 2	300ng	121488	25145	4.83
WT Mucosa 3	300ng	4404	1392	3.16
dLN 1	500ng	74528	26892	2.77
dLN 2	500ng	62460	28414	2.2
dLN 3	500ng	39266	1781	22.05
Spleen 1	500ng	61992	27290	2.27
Spleen 2	500ng	64709	28596	2.26
Spleen 3	500ng	75714	28323	2.67

Supplementary Table 2: Sequencing information for samples submitted for scRNA-seq related to Figure 2.

Sample ID	No. Cells	Mean Reads/Cell	Median Genes/Cell	No. Reads	Valid Barcodes
Tumor T_{RM}	2273	473741	1228	1076814147	0.969
Tumor T_{Eff} / T_{EM}	6859	122127	1060	837674652	0.976
Tumor T_{CM}	9424	77281	1127	728297585	0.977
Distant Mucosa T_{RM}	3679	50324	1155	185144103	0.971

Sample ID	Sequencing Saturation	Q30 Bases in UMI	Reads Mapped to Genome	Total Genes Detected	Median UMI Counts/Cell
Tumor T_{RM}	0.971	0.959	0.864	14361	2915
Tumor T_{Eff} / T_{EM}	0.935	0.96	0.899	15152	2877
Tumor T_{CM}	0.883	0.96	0.9	15529	3659
Distant Mucosa T_{RM}	0.845	0.979	0.944	14616	3271

Supplementary Table 3: QC analysis of TCRβ repertoire sequencing samples related to Figure 6.

Sample ID	Cells Sorted	Total Mapped Reads	Unique TCR Clonotypes	Mean Read Per TCR
CXCR6+	8769	21410	6414	3.34
CXCR6-	9674	15129	5529	2.74
Tumor Trm	13000	113246	11730	9.65
Distant Trm	11600	15554	4074	3.82
