# Supplementary material for: Resident memory T cells in tumor-distant tissues fortify against metastasis formation
Source: Cell Rep. Author manuscript; Available in PMC 2021 Jun 15. (PMC8204287; doi:10.1016/j.celrep.2021.109118)
Supplement: 2 [file NIHMS1703505-supplement-2.pdf]

Cell Reports

Article

Resident memory T cells in tumor-distant tissues fortify against metastasis formation

Graphical abstract

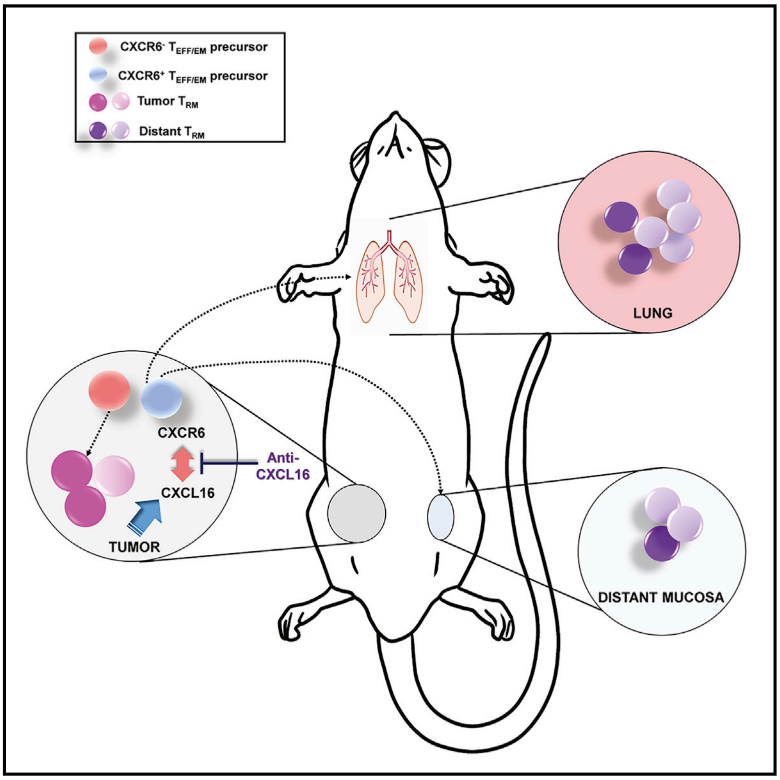

Highlights

- T_{RM}s in tumors and distant tissues are developed from $T_{Eff/EM}$ precursor cells
- T_{RM}s comprise active and quiescent subsets that resemble T_{EM}s and T_{CM}s
- Tumor-specific T_{RM}s reside in distant tissues before tumor metastasis
- CXCL16 in tumor microenvironment traps T_{RM} precursors to facilitate metastasis

Authors

Laura S. Christian, Liuyang Wang, Bryan Lim, Dachuan Deng, Haiyang Wu, Xiao-Fan Wang, Qi-Jing Li

Correspondence

qi-jing.li@duke.edu

In brief

Christian et al. show that tumor-derived resident memory T cells (T_{RM}s) are developed in tumors, as well as in distant and tumor-free mucosal tissues. Distant T_{RM}s are developed from $T_{Eff/EM}$ precursors that are trapped within the tumor. The release of such retention can be achieved by CXCR6/CXCL16 blockade, which leads to prevention against metastasis.

Christian et al., 2021, Cell Reports *35*, 109118
May 11, 2021 © 2021 The Author(s).
https://doi.org/10.1016/j.celrep.2021.109118

CellPress

Cell Reports

CellPress
OPEN ACCESS

Article

Resident memory T cells in tumor-distant tissues fortify against metastasis formation

Laura S. Christian,[1,5] Liuyang Wang,[2,5] Bryan Lim,[1] Dachuan Deng,[3] Haiyang Wu,[3] Xiao-Fan Wang,[4] and Qi-Jing Li[1,6,*]
[1]Department of Immunology, Duke University Medical Center, Durham, NC 27710, USA
[2]Department of Molecular Genetics and Microbiology, Duke University Medical Center, Durham, NC 27710, USA
[3]TCRCure (TianKeYa) Biopharma, Ltd., Durham, NC 27701, USA
[4]Departments of Pharmacology and Cancer Biology, Duke University Medical Center, Durham, NC 27710, USA
[5]These authors contributed equally
[6]Lead contact
*Correspondence: qi-jing.li@duke.edu
https://doi.org/10.1016/j.celrep.2021.109118

SUMMARY

As a critical machinery for rapid pathogen removal, resident memory T cells (T_{RM}s) are locally generated after the initial encounter. However, their development accompanying tumorigenesis remains elusive. Using a murine breast cancer model, we show that T_{RM}s develop in the tumor, the contralateral mammary mucosa, and the pre-metastatic lung. Single-cell RNA sequencing of T_{RM}s reveals two phenotypically distinct populations representing their active versus quiescent phases. These T_{RM}s in different tissue compartments share the same TCR clonotypes and transcriptomes with a subset of intratumoral effector/effector memory T cells ($T_{Eff/EM}$s), indicating their developmental ontogeny. Furthermore, CXCL16 is highly produced by tumor cells and CXCR6^{-} $T_{Eff/EM}$s are the major subset preferentially egressing the tumor to form distant T_{RM}s. Functionally, releasing CXCR6 retention in the primary tumor amplifies tumor-derived T_{RM}s in the lung and leads to superior protection against metastases. This immunologic fortification suggests a potential strategy to prevent metastasis in clinical oncology.

INTRODUCTION

The formation of memory T cells is the hallmark of adaptive immunity, which provides rapid and robust protection against bacteria, virus, or tumor during antigen re-encounter. Classically, memory T cells are categorized into two major phenotypes: central memory T cells (T_{CM}s) and effector memory T cells (T_{EM}s) (Sallusto et al., 1999). T_{CM}s are long-lived, quiescent, and stem cell-like. They bear the chemokine receptor CCR7 and the cell adhesion molecule CD62L, allowing them to enter and patrol secondary lymphoid organs (Unsoeld et al., 2002; von Andrian and Mackay, 2000). Upon antigen recognition, they differentiate with multipotent capacity (Williams and Bevan, 2007). In contrast, T_{EM}s have a shorter lifespan but are more poised for activation. T_{EM}s circulate and enter peripheral non-lymphoid tissues, where they exert effector functions for swift and robust pathogen control (Fuhlbrigge et al., 1997; Mackay et al., 1992). A decade ago, the guardian functions of T_{EM}s in non-lymphoid tissues were reattributed to a novel non-circulating subset of memory T cells, resident memory T cells (T_{RM}s) (Masopust et al., 2001). Using CD103 (*Itgae*), a specified integrin molecule that binds E-cadherin on the epithelial barrier (Cepek et al., 1994), T_{RM}s reside in mucosal tissues and are an integral component of the adaptive immune machinery against viral re-challenge, including vaccinia virus, influenza, and herpes simplex virus (HSV) (Ariotti et al., 2014; Iijima and Iwasaki, 2015; Jiang et al., 2012; Teijaro et al., 2011; Wu et al., 2014). Upon encountering infected cells, such as T_{EM}s, T_{RM}s activate quickly. However, unlike T_{EM}s, T_{RM}s release chemokines to recruit T cells, including circulating T_{EM}s, to the infected tissue for intensified immune protection (Schenkel and Masopust, 2014).

While their pivotal roles in the antiviral response are well appreciated, the ontogeny of T_{RM}s remains elusive. In a study using a skin immunization model and high-throughput T cell receptor-β (TCR-β) sequencing, T_{CM}s and T_{RM}s were found to develop from the same naive T cell clone (Gaide et al., 2015). A single-cell RNA sequencing (scRNA-seq) study with the acute lymphocytic choriomeningitis virus (LCMV) infection model found that early effector cells with high interleukin-2 receptor α (IL-2rα) and Ezh2 expression were predisposed to become T_{RM}s (Kurd et al., 2020). Specifically, Gerlach et al. (2016) showed that during LCMV infection, CX3CR1^{-} effector cells were a common precursor of T_{CM}s and T_{RM}s. While circulating CX3CR1^{int} peripheral memory cells (T_{PM}s) can also survey the tissues, T_{EM}s arise from CX3CR1^{hi} cells and are prohibited from tissue entry. In both vaccinia virus (VACV) infection and B16 melanoma transplantation models, transferred T_{CM} cells were found to differentiate directly into T_{RM}s (Enamorado et al., 2017). By contrast, in an influenza airway infection model, only transferred T_{EM}s were able to become lung T_{RM}s (Slütter et al.,

This is an open access article under the CC BY license (http://creativecommons.org/licenses/by/4.0/).

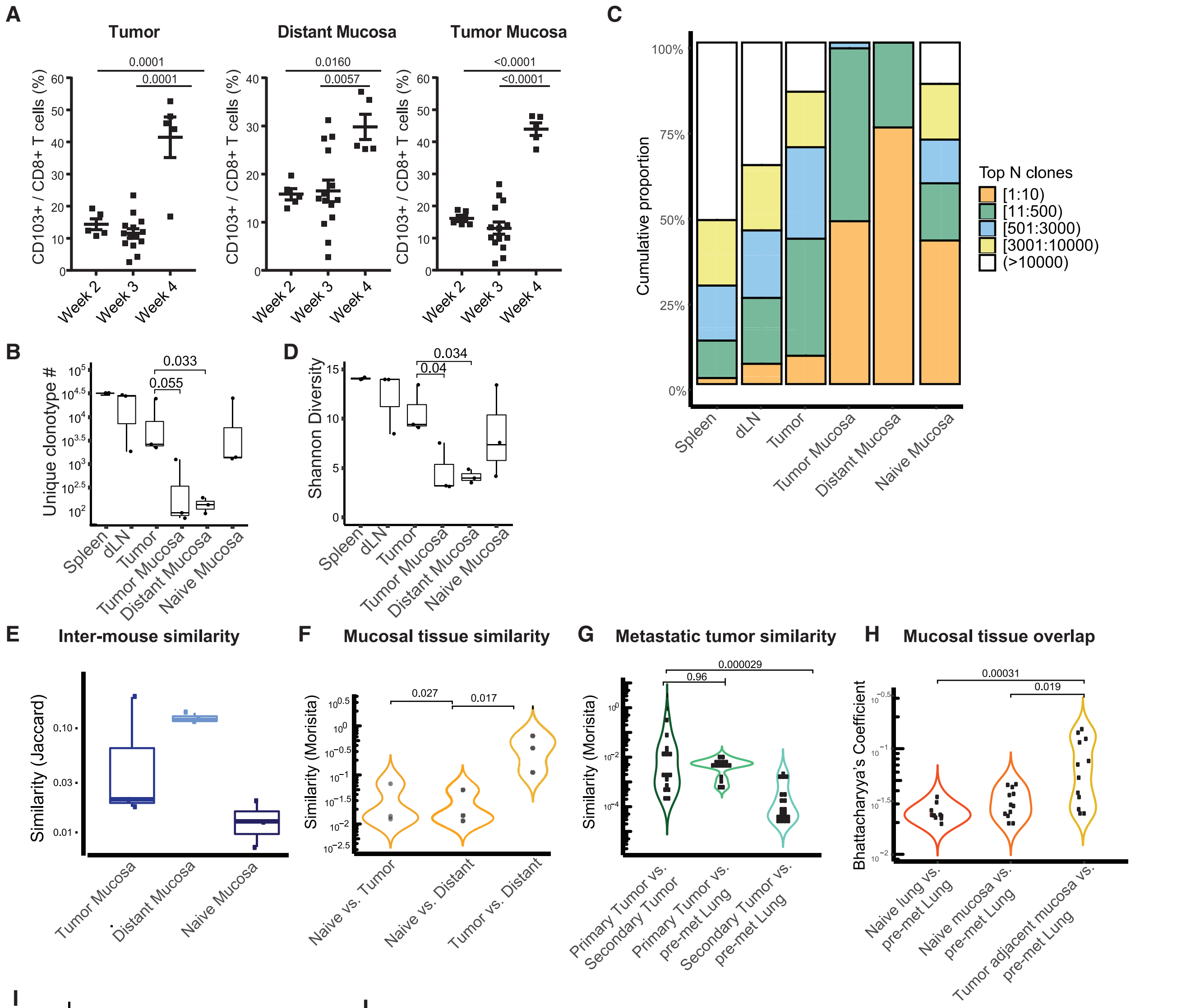

I

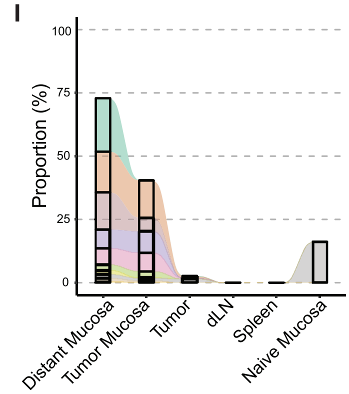

J

TCR CDR3 Sequence	Frequency In Distant Mucosa	In Tumor Mucosa	In Tumor	In Naïve Mucosa
CASSLASRGQGASRNQDTQYF	21.100%	0.017%	0.204%	0.039%
CASSLAGVGQGAGVSGNTLYF	16.120%	14.726%	0.599%	0.009%
CASSLSYQGQGAYQSAETLYF	14.657%	5.343%	N/A	0.006%
CASSQESQGASHSYEQYF	7.420%	8.523%	0.097%	0.005%
CASGDARQDRGPRCSGNTLYF	6.496%	7.303%	0.214%	0.019%
CASSQDTVGLGGPRSNSDYTF	2.181%	2.486%	0.034%	N/A
CASSQDTVGLGRPRSNSDYTF	2.181%	0.176%	N/A	N/A
CASSLNWSQDTQYF	1.486%	0.991%	1.510%	16.087%
CASSLSLGTGGAHYNYAEQFF	1.342%	0.480%	0.017%	N/A
CASSLSYQGQEAYQSAETLYF	0.459%	0.357%	N/A	N/A

Figure 1. TCR-β repertoire sequencing identifies extensive sharing between tumor and distant mucosa T_{RM}s

(A) Flow cytometry analysis of $CD8^{+}CD103^{+}T_{RM}$s in the tumor, tumor mucosa, and distant mucosa. One-way ANOVA with Tukey's multiple comparison test, $p < 0.05$. Data are pooled over 3 independent experiments.

(B) Analysis of the unique clonotype number between samples. Wilcoxon signed rank test, $p < 0.05$.

(C) Clonal T cell expansion in the tumor and tumor-associated tissues.

(D) Shannon diversity index shows a reduction of $CD8^{+}$ T cell diversity in the tumor, tumor mucosa, and distant mucosa. Wilcoxon signed rank test, $p < 0.05$.

(legend continued on next page)

2017). More recently, with a *Listeria monocytogenes* (LM) infection model, circulating KLRG1^{-} cells are suggested as a common precursor subset for all memory T cell populations, including T_{CM}, T_{EM}, CX3CR1^{int} T_{PM}, and T_{RM} (Herndler-Brandstetter et al., 2018). These data suggest that the model of T_{RM} induction and their residing tissue environment play a significant role in T_{RM} development.

Tumorigenesis is a chronic process and established tumors are composed of a complex microenvironment. Consequently, some identified characteristics of tumor T_{RM}s are surprising given our current knowledge of T cell memory. Using scRNA-seq, T_{RM}s in human breast tumors were shown to be similar to terminally differentiated intratumoral effector/effector memory T cells ($T_{Eff/EM}$s); they were Klrc1^{hi}PD-1^{hi}Tim-3^{hi}, but enriched with cytolytic molecules such as granzyme B (Savas et al., 2018). T_{RM}s from murine B16 tumors could separate into two populations: one being Blimp1^{hi} and short-lived, similar to the population found in human breast tumors, and the other, Id3^{hi}, is a marker of long-lived T_{RM} cells (Milner et al., 2020). Nevertheless, a consensus has been reached that T_{RM}s play an important role in overall antitumor immunity. In mice, the presence of tumor antigen-specific T_{RM}s in the skin, either induced by engineered HSV (Park et al., 2019) or established through previous tumor challenge (Malik et al., 2017), protected mice from transplanted B16 melanoma. For patients with breast, lung, or ovarian cancer, the abundance of CD8^{+}CD103^{+} T_{RM}s strongly correlated with longer disease-free survival (Djenidi et al., 2015; Wang et al., 2016; Webb et al., 2014). Since tumor metastases present a much more prominent threat to patients' lives than their primary tumor, it is critical to establish whether tumor-specific T_{RM}s can be induced outside the primary tumor site and whether they play protective roles against metastasis.

Using high-throughput TCR-β repertoire sequencing on a cohort of gastric cancer patients, our group previously identified CD8^{+}CD103^{+} T_{RM}s in the tumor-adjacent mucosa of gastric tumors. Compared with their peripheral counterparts, tumor-infiltrating T cells had a restricted TCR-β repertoire and oligoclonal expansion, resulting in reduced clonotype diversity. Through repertoire analyses of T cells within the adjacent, tumor-free mucosal area, we detected a significant proportion of CD103^{+} T_{RM}s that share the same TCR with highly expanded T cell clones found in the tumor. Furthermore, the diversity of these T cells correlated with patients' long-term prognosis, suggesting their protective role against gastric cancer recurrence and metastasis (Jia et al., 2015).

In this study, using a murine breast cancer model, we used scRNA-seq to analyze the heterogeneity of memory T cell subsets in the tumor. Specifically, we focused on the lineage progression of tumor T_{RM}s. We characterized fully differentiated T_{RM}s residing in the tumor and distant (non-tumor-adjacent) mammary mucosal tissues. Through single-cell transcriptome mapping and TCR clonotype tracking, we determined that distant mucosal T_{RM}s derived from a particular T cell effector/effector memory intratumoral population that lacked CXCR6 expression. Releasing T cells from primary breast tumors by breaking CXCR6-mediated retention led to enhanced protection against tumor metastasis in the distant lung.

RESULTS

Resident memory T cells develop in tumor and distant tumor-free mucosal tissues

To study tumor-resident memory CD8^{+} T cells, we used the orthotopic 4T1 triple negative breast tumor model. We titrated tumor-inducing doses of 4T1 cells and determined that implanting 100 4T1 cells into the mammary fat pad had 100% penetrance to form primary tumors in 3 weeks, without visibly detectable lung metastases until 5 weeks (Figure S1A). At different time points post-tumor inoculation, we harvested the tumor and tumor-adjacent mucosa to assess the development of tumor T_{RM}s (Figure S1B). While we initially included the distant (tumor-free) mammary gland mucosa as a T_{RM}-free control, we found that CD8^{+}CD103^{+} T_{RM}s developed in all tissues and their frequency increased with tumor growth, even in the distant mucosa (Figure 1A).

To assess the overall structure of the T cell repertoire within different compartments, we performed TCR-β deep sequencing without T cell purification, on multiple tissues collected from tumor-bearing mice. The mammary gland from tumor-naive mice was included as a control (Table S1). Within the tissues from tumor-bearing mice, using the spleen as the standard, we found that the number of unique TCR clonotypes was lower in the tumor and further contracted in the tumor-adjacent and distant mammary gland mucosa (Figure 1B). This clonotype contraction was accompanied by oligoclonal expansion. Approximately 10,000 different TCR clones occupied ~50% of the repertoire space in the spleen; a similar amount of space was occupied by slightly more than 500 clonotypes in the tumor and 10 clonotypes in the tumor-adjacent mucosa; most striking, 10 different clones of T cells accounted for 75% of the total T cell repertoire within the distant mucosa (Figure 1C). Accordingly, when the TCR diversity was measured by Shannon entropy, which is determined by both the clonotype number and expansion level of individual clonotypes, we found that T cell diversity was reduced in the tumor, tumor-adjacent mucosa, and

(E) Jaccard similarity analysis compares distinct T cell clones in the tissues of individual mice.
(F) Global T cell similarity analysis uses the Morisita index to measure the presence and abundance of clonotypes between mucosal tissues. Wilcoxon signed rank test, $p < 0.05$.
(G) Morisita index evaluates clonotype similarity between primary and secondary (metastatic) lung tumor and pre-metastatic lung tissues. Wilcoxon signed rank text, $p < 0.05$.
(H) Bhattacharyya's coefficient analysis evaluates the clonal overlap between mammary and lung mucosal tissues. Wilcoxon signed rank test, $p < 0.05$.
(I) Analysis of the 10 most expanded TCRs in the distant mucosa and their overlap between other tissues.
(J) TCR CDR3 sequences of the top 10 most expanded distant mucosa clones are listed on the left and their frequency in other tissues is displayed as a percentage. Bars represent means ± SEMs and symbols represent individual mice.

tumor-distant mucosa as a cascade (Figure 1D). Evidently, as also found in the mammary gland mucosa of female monkeys, the mammary mucosa of tumor-naive mice harbored T cells (Sircar et al., 2010). However, T cells residing in this environment have a much more diverse repertoire compared to tumor-bearing mice (Figures 1B–1D). In summary, these data suggest that in tumor-bearing mice, T cell residency within the mammary gland mucosa selects for a limited population of T cells.

To track the clonal origins of these T cells, we compared the similarity of TCR clonotypes within the mucosal tissue compartments among different mice. Using the Jaccard index, we measured the ratio of clonotypes overlapping between two specific compartments. When the mammary gland mucosa was compared among different animals, more T cell clonotypes in the tumor-adjacent mucosa were shared among different tumor-bearing mice than among individual tumor-naive mice. This clonotype sharing was more evident for T cells in the distant mammary gland tissues of tumor-bearing mice (Figure 1E). Considering that only select clonotypes reside within the mammary gland mucosa, this heightened TCR similarity among tumor-bearing mice suggests that some common antigens produced by 4T1 tumors may play a role in this selection.

We also performed a global T cell similarity analysis between different types of mucosal tissues. Using the Morisita index, we took both clonotype amounts and abundance into account (Venturi et al., 2008). We found little repertoire similarity between T cells within the tumor-naive and tumor-adjacent mucosa or between T cells from the tumor-naive and tumor-distant mucosa. However, the overall similarity between T cells in the tumor-adjacent and distant mucosa from tumor-bearing mice was quite high (Figure 1F). Since the lung is the primary site of 4T1 spontaneous metastasis, we harvested the pre-metastatic lung mucosa tissue at 3 weeks post-tumor inoculation (Figure S2), as well as the later-developed lung metastatic (secondary tumors) for TCR repertoire analysis. We found that clonotypes overlapped between the primary tumor in the breast and metastatic tumors in the lung. The clonotype sharing, especially for the dominant clones favored by the Morisita index, between the primary tumor and pre-metastatic lung was as high as the sharing between the primary and secondary tumors (Figure 1G). This coordinates with our similarity findings between the primary tumor and the distant mammary gland. In addition, the similarity between the pre-metastatic lung and the primary tumor was higher than that between the pre-metastatic lung and the lung metastases. This suggested that either there are new infiltrates after the establishment of metastasis or there are T cell clonotypes that expand or contract, resulting in the change in dominant T cell clones. Furthermore, the overall sharing of clonotypes (measured by Bhattacharyya's coefficient after normalization) between the pre-metastatic lung tissues and distant mammary gland mucosa was significantly elevated compared to the sharing between the pre-metastatic lung and either the mammary gland mucosa or lung tissues from tumor-naive mice (Figure 1H). These data strongly suggested that before the establishment of metastases, the same expanded T cell clones egressed the primary tumor and infiltrated the distant mammary gland and pre-metastatic lung tissue.

Zooming in on the most abundant (top 10) clonotypes in the distant mucosa, we found that each individual clone could be identified in the tumor-adjacent mucosa and 6 of 10 were highly expanded (>0.5%) clones (Figures 1I and 1J) (Jia et al., 2015). In addition, 7 of 10 of these clones could also be identified within the 4T1 tumor (Figures 1I and 1J). Notably, these TCRs, except one, could not be found in the mucosa of tumor-naive mice. This deep sequencing-aided clonal lineage tracing indicated that resident T cells found in the tumor adjacent and distant mucosa share common precursors, which most likely originate from T cells in the tumor.

scRNA-seq dissects intratumoral $T_{Eff/EM}$ heterogeneity

While the heterogeneity of CD103^{+} T_{RM}s is widely discussed in the literature, the characteristics of T_{RM} subpopulations within the tumor or distant tissues of tumor-bearing mice are less well defined. To characterize tumoral T_{RM}s, scRNA-seq technology provides unprecedented analytical power and allows us to dissect cells with thousands of dimensions. With the hypothesis that particular intratumoral T cells are precursors for T_{RM} development, we extended our scRNA-seq approach to include various effector and memory T cell populations to comprehensively depict their transcriptomic program. From the tumor and distant mucosa of mice bearing established 4T1 tumors, we flow sorted distinct memory T cell populations using a strict gating strategy (Figure S3) (Buenrostro et al., 2018). We constructed single-cell cDNA libraries from paired intratumoral sorted TCR-β^{+}CD44^{+}CD62L^{-}CD69^{+}CD103^{+} cells (T_{RM}s), mucosal sorted TCR-β^{+}CD44^{+} CD69^{+}CD103^{+} cells (distant mucosa T_{RM}s), intratumoral TCR-β^{+}CD44^{+}CD62L^{+}CD69^{-}CD103^{-} cells (T_{CM}s), and TCR-β^{+}CD44^{+}CD62L^{-}CD69^{-}CD103^{-} cells (T_{EM}s) (Table S2). After data processing and normalization, we performed unbiased clustering and visualized the CD8^{+} clusters using t-distributed stochastic neighbor embedding (t-SNE; Figure 2A) (Kobak and Berens, 2019). Through this unsupervised analysis, we found that all four samples could distinctly separate into multiple clusters, confirming continued heterogeneity among the single-cell subsets.

In comparison to T_{CM}s, intratumoral $T_{Eff/EM}$s were a more heterogeneous population, which could largely be divided into four subsets, p1–p4. The effector molecules *Ifng* and *GzmB* were highly expressed in p4, had heterogeneous expression in p1 and p3, but were largely absent from p2. Similar expression patterns were applied to well-known effector surface markers for cytolytic T cells such as *Klrc1* and *Nkg7* (Figure 2B). From this, we reasoned that p4 is highly enriched by effector cells. All four subsets highly expressed the transcription factor *Runx3*, which is essential for the cytotoxic program (Cruz-Guilloty et al., 2009), as well as *Id2*, which is upregulated to support the effector phase of cytotoxic T lymphocytes (CTLs) (Cannarile et al., 2006) responses. Comparing p2 to p4, the density of Runx3- and Id2-expressing cells was slightly lower in p2. This was accompanied by an opposite pattern of Id3, a transcription factor whose expression is crucial for effector memory development (Yang et al., 2011), which is absent in p4 (Figure 2C). We concluded that $T_{Eff/EM}$ p4 is enriched by

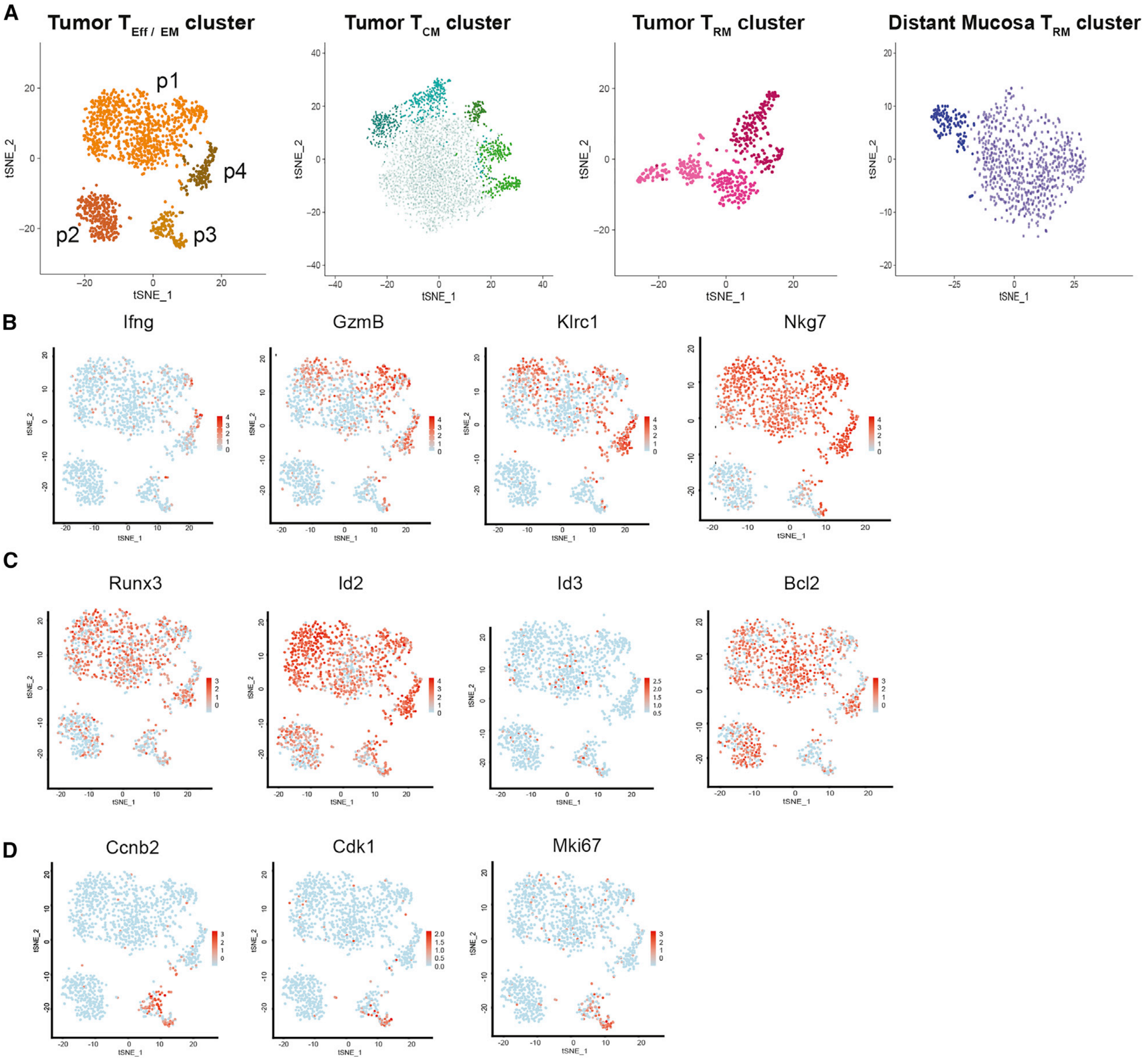

Figure 2. Single-cell RNA sequencing reveals intratumor $T_{Eff/EM}$ heterogeneity

(A) Independent tSNE plots of sorted live TCR-β^+CD44$^+$CD62L$^-$CD69$^-$CD103$^-$ tumor $T_{Eff/EM}$, TCR-β^+CD44$^+$CD62L$^+$CD69$^-$CD103$^-$ tumor T_{CM}, TCR-β^+CD44$^+$CD62L$^-$CD69$^+$CD103$^+$ tumor T_{RM}, and TCR-β^+CD44$^+$CD69$^+$CD103$^+$ distant mucosa T_{RM} single-cell transcriptomes obtained from 10 tumors and matched distant mucosa. Each dot represents a cell; each color indicates a distinct CD8$^+$ T cell cluster.

(B–D) $T_{Eff/EM}$ CD8$^+$ clusters are labeled p1–p4, and the expression of (B) effector molecules and activation markers, (C) transcription factors, and (D) proliferation markers in the tumor $T_{EFF/EM}$ clusters are shown.

differentiated effector CTLs and T cells in the p2 subset are at a more advanced T_{EM} stage. *Bcl2*, a transcription factor for T cell survival in the effector and memory phases, was abundant in most sorted $T_{Eff/EM}$ populations except p3. After assessing the cell-cycle programs, we determined that p3 was a highly proliferative subset, as made evident by the expression of genes restrictively expressed in S and M phases, such as *Ccnb2*, *Cdk1*, and *Mki67* (Figure 2D). We reasoned that the p2 subset is constituted by proliferative and less differentiated T_{Eff} cells.

Tumor and distant mucosa T_{RM}s comprise two distinct populations that resemble either T_{EM}s or T_{CM}s

Of most interest to the present study is that both the tumor T_{RM} and distant mucosa T_{RM} populations independently clustered into three and two major groups, respectively. As expected, the tumor and distant mucosa T_{RM} populations showed high expression of *Itgae* (CD103) and low expression of *S1PR1*, confirming their tendency to reside within the tissue (Figure S4). Choosing the major subsets of T_{CM}s and T_{EM}s as references (p1 of $T_{Eff/EM}$), we interpreted these T_{RM} subpopulations using

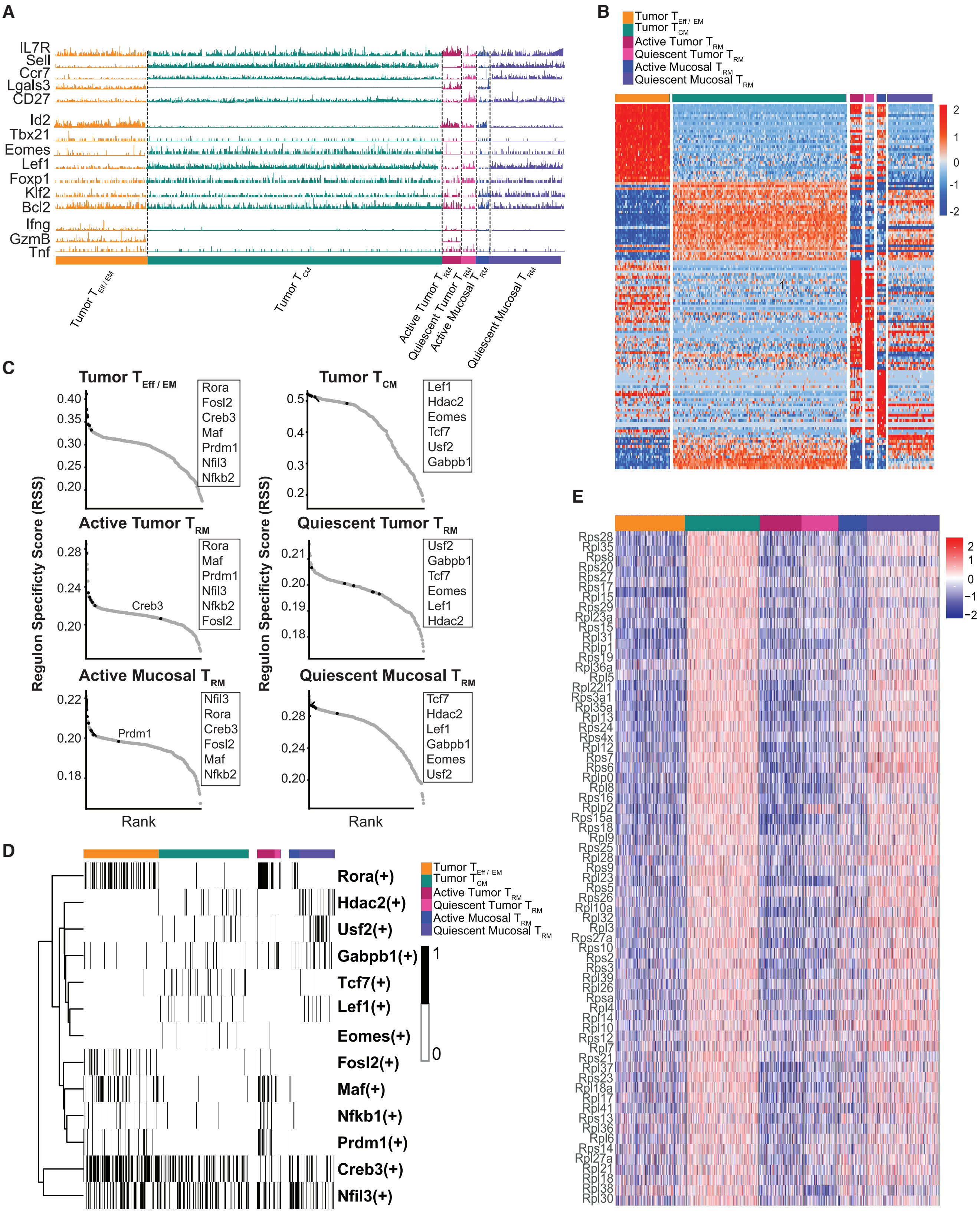

(legend on next page)

a candidate approach by evaluating their surface markers, transcription factors, and effector molecules. We found a subset of T_{RM}s that shared lineage characteristics with T_{EM}s, indicating that this subset of T_{RM}s was maintained at a functionally active stage, hereafter called "active T_{RM}s." Specifically, we noted that (1) in active T_{RM}s, similar to their $T_{Eff/EM}$ counterparts, *Sell* expression was completely suppressed (Sallusto et al., 1999) and *Ccr7* expression was severely reduced. Notably, the binary expression of *Lgals3* (galectin-3) could distinguish T_{EM}s from T_{CM}s, as well as active T_{RM}s from quiescent T_{RM}s. This is consistent with previous findings that optimal galectin-3 induction requires TCR signaling (Joo et al., 2001), and in the tumor microenvironment, galectin-3 is differentially expressed on the surface of tumor-antigen activated $CD8^+$ T cells, but is absent in resting T cells (Joo et al., 2001; Kouo et al., 2015). (2) Similar to that in $T_{Eff/EM}$s, *Id2* (Cannarile et al., 2006) was highly elevated in the active T_{RM} subsets. Of note, since the $CD8^+$ T cells in our analysis are memory phenotype enriched, transcription factors that preferentially support early effector function, such as *Tbx21* (T-bet) (Intlekofer et al., 2005), could be detected occasionally. Nevertheless, more frequent *Tbx21* expression was observed in active T_{RM}s than quiescent T_{RM}s. (3) Similar to $T_{Eff/EM}$s, *Ifng* and *GzmB* were preferentially expressed in active tumor T_{RM}s (Figure 3A).

Reciprocally, within both the tumor and distant mammary gland mucosal compartments, we found another subset of T_{RM}s that obtained gene expression features resembling T_{CM}s, representing a quiescent, long-lived resident memory T cell population, hereafter called "quiescent T_{RM}s." Quiescent T_{RM}s could be found in both the tumor and distant mucosa compartments. Specifically, in quiescent T_{RM}s, we noted the following: (1) the T_{CM} surface markers *Sell* (CD62L) and *CCR7* were highly expressed. While CD27 was widely expressed in all of the selected subsets, its expression was elevated in the quiescent subsets, suggesting its naive-like feature (van Lier et al., 1987). (2) T_{CM}-associated transcription factors such as *Eomes* (Pearce et al., 2003), *Lef1* (Zhou and Xue, 2012), and *Foxp1*(Feng et al., 2010) were highly expressed in the quiescent T_{RM} population. (3) As in T_{CM}s, the expression of functional molecules associated with cytolytic killing, such as *Ifng*, *GzmB*, and *Tnf* were silenced (Figure 4A). T_{RM}s, both in the tumor and mammary gland mucosal compartments, could be largely divided into two functional phenotypes based on their signature gene expression.

The $T_{Eff/EM}$ versus T_{CM} phenotypic division was also validated by global differences in their transcriptomes. We took the expression levels of the top 100 differentially expressed genes (DEGs) in the intratumoral T_{EM} and T_{CM} populations as a benchmark. When this unbiased control was applied to different T_{RM} subsets, it depicted the major differences between active and quiescent T_{RM}s (Figure 3B).

To examine whether these transcriptomic differences represented a globally orchestrated differentiation event, we performed transcriptomic regulon analysis. The SCENIC (single-cell regulatory network inference and clustering) algorithm was used to identify co-expressed gene modules that share common *cis*-regulatory elements for a specific transcription factor (Aibar et al., 2017). Based on p values that reflect the co-expression coefficients and enrichment of *cis*-regulatory elements, we ranked the transcription factor regulons in classical $T_{Eff/EM}$s and T_{CM}s. Although the ranking order may vary, the majority of the top-ranked $T_{Eff/EM}$ regulons such as *Rora* (Best et al., 2013), *Fosl2* (Ciofani et al., 2012), *Creb3* (Chan et al., 2011), *Maf* (Ciofani et al., 2012), *Prdm1* (Kallies et al., 2009), *Nfil3* (Kashiwada et al., 2011), and *Nfkb1* (Best et al., 2013) were also identified as top-ranked regulons for active T_{RM}s in the tumor and distant mammary mucosa. Reciprocally, all top-ranked T_{CM} regulons, such as *Lef1* (Zhou and Xue, 2012), *Hdac2* (Shin et al., 2013), *Eomes* (Pearce et al., 2003), *Tcf7* (Zhou et al., 2010), *Usf2* (Suo et al., 2018), and *Gabpb1* (Luo et al., 2017), were significantly enriched in the transcriptome of quiescent T_{RM}s (Figures 3C and 3D). This analysis revealed that the transcriptomic distinctions between active and quiescent T_{RM}s are controlled by the same underlying gene regulatory networks that specify T_{EM} versus T_{CM} development.

While investigating the DEGs between these memory T cell populations, we found an unexpected enrichment of a large cluster of ribosome-related genes that were distinct between intratumor $T_{Eff/EM}$s and T_{CM}s. While this differential expression was less pronounced between intratumoral T_{RM}s, expression of these ribosome genes was dramatically upregulated in the quiescent distant T_{RM} population compared to active distant T_{RM}s (Figure 3E). The association between an upregulated ribosome gene profile and a relatively quiescent T cell phenotype was a common feature identified through our analyses.

Tissue environment plays a significant role in shaping the transcriptome of T_{RM}s

To trace the developmental path of T_{RM}s, we subjected all tumor $T_{Eff/EM}$ and T_{RM} cells to the Monocle2 algorithm for pseudotime analysis (Qiu et al., 2017; Trapnell et al., 2014). Our assumption was that within the tumor, T cells differentiate or develop asynchronously. The moment of sample collection represents a snapshot, in which each individual T cell may be fixed at a specific stage of differentiation or development. Consequently, differentiation and developmental processes of the T cells can be revealed by arranging individual cells on a time trajectory based on their gradual and continuous transcriptomic transition. Hence, pseudotime analysis allowed us to determine the relative position of each individual cell on this time trajectory. Since the relative positioning of cells cannot automatically determine the

Figure 3. Two distinct subsets that resemble either T_{EM}s or T_{CM}scomprise tumor and distant mucosa T_{RM}s

(A) GeneTrac analysis of surface markers, transcription factors, and effector molecules among the major tumor $T_{Eff/EM}$, T_{CM}, T_{RM}, and distant T_{RM} populations.

(B) Heatmap of top 100 DEGs between the tumor $T_{Eff/EM}$ and tumor T_{CM}s shows the global gene expression pattern between these circulating memory T cells and active and quiescent T_{RM}s.

(C) SCENIC regulon analysis reveals an enrichment of the top regulons between tumor $T_{Eff/EM}$ and active T_{RM}s and between tumor T_{CM}s and quiescent T_{RM}s.

(D) Binary heatmap for top regulons is plotted for each cluster.

(E) A global enrichment of ribosome genes associates with tumor T_{CM} and quiescent T_{RM} subsets.

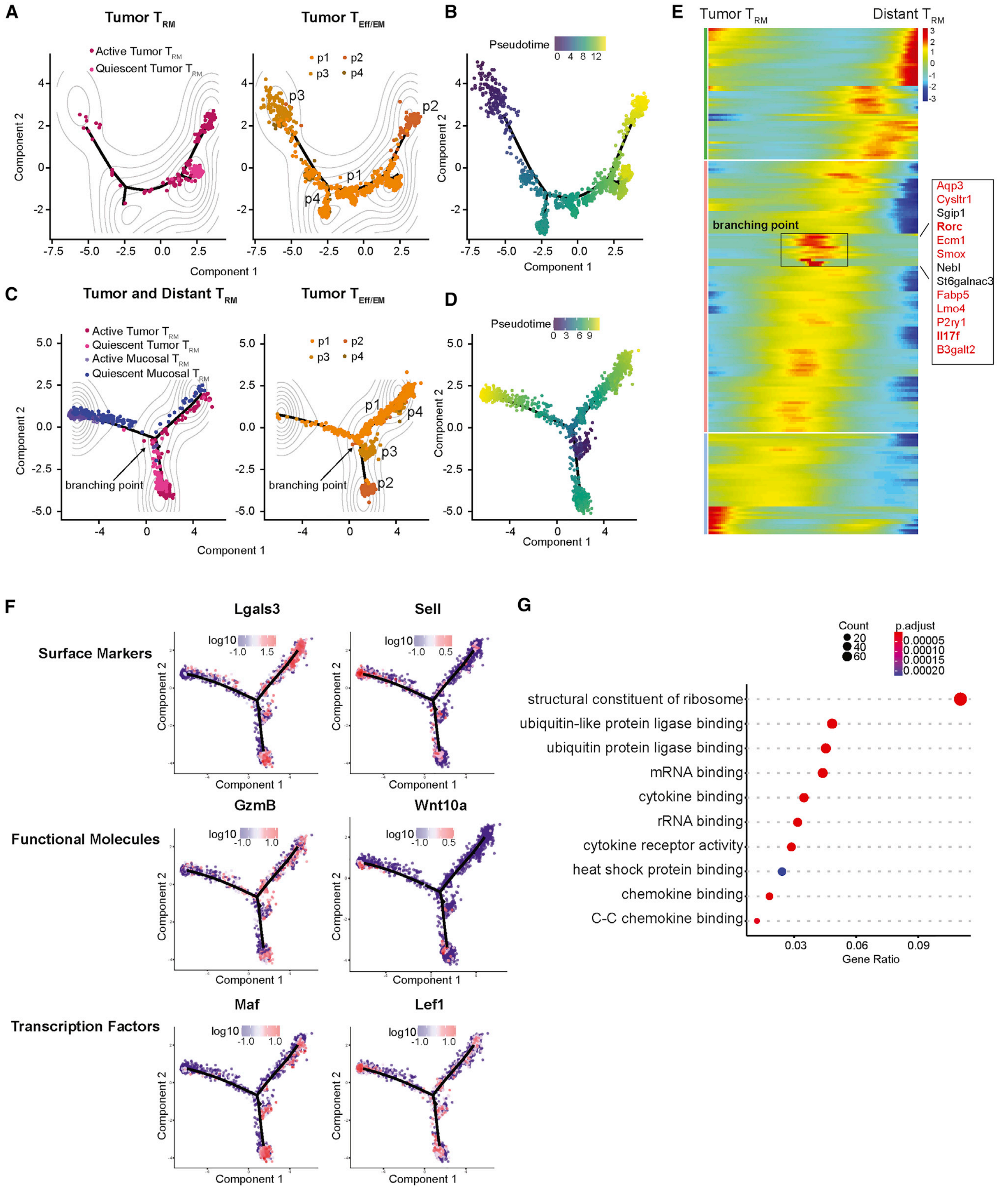

Figure 4. Tissue environment plays a significant role in shaping the formation of T_{RM}s

(A and B) Monocle2 analysis shows the lineage relationship and progression between the 4 tumor $T_{Eff/EM}$ $CD8^+$ subsets and tumor T_{RM}s in (A) contour and (B) pseudotime plot. The main proliferating population (tumor $T_{Eff/EM}$ p3) was used as the starting point of pseudotime progression in all of the analyses.

(legend continued on next page)

beginning or end of the pseudotime, we designated the least differentiated $T_{Eff/EM}$ population with the highest proliferative capacity ($T_{Eff/EM}$ p3; Figure 2D) as the starting point. This is a reasonable assumption because T cell proliferation in the effector phase proceeds terminal effector and memory T cell differentiation, including the development of T_{RM}s.. In our pseudotime plot, cells found in the heterogeneous p1 subset directly connected to the p3 population, which spread widely on the development tree. This "synthetic" developmental process had two branched ends: one was a tight cluster encompassing a majority of quiescent T_{RM}s; the other was enriched by highly differentiated p2 T_{EM}s and active T_{RM}s (Figures 4A and 4B). This suggested that by stemming from T_{Eff}s (p3), T_{EM}s and T_{RM}s may share a similar development process until they reach their final branching point.

In both the transcriptome and regulon analyses, we noticed that the distinction between quiescent and active T_{RM}s in the tumor was less profound than that in the distant mammary gland mucosa (Figure 3B–3D). This blurred distinction was also reflected by the differential expression of ribosome-related genes (Figure 3E), suggesting that quiescent T_{RM}s in the tumor are relatively "activated" in comparison to their counterparts in the distant mammary gland. To test the impact of different tissue microenvironments and to explore the lineage progression paths leading to distant T_{RM} development, we added T_{RM}s collected from the distant mammary gland to the Monocle2 algorithm. Choosing the same proliferating p3 subset as the starting point, we made three major observations (Figures 4C and 4D): (1) except for some active distant T_{RM}s that co-localized with active tumor T_{RM}s, the majority of $T_{Eff/EM}$ and T_{RM} cells were separated by their tissue of origin, with the mucosal T_{RM} subsets occupying a different space on the contour plot from the tumor memory T cell subsets. It was unexpected to observe that the impact of the tumor or distant mucosa location could overshadow the intrinsic transcriptomic differences between $T_{Eff/EM}$s and T_{RM}s. (2) A few tumor $T_{Eff/EM}$ p1 cells crossed the tissue "boundary" to overlap with distant mucosa T_{RM}s. This revealed that there are $T_{Eff/EM}$ cells with a transcriptome that mimics distant T_{RM}s, although their surfaces are absent of CD103 expression and they are still located in the tumor. (3) A transitioning population of tumor $T_{Eff/EM}$ cells was located at a critical branching point. These precursor $T_{Eff/EM}$ cells largely belong to p1 and p4 subsets and leave this branching point with lineage decisions to become tumor T_{RM}s or distant T_{RM}s.

We projected the transcriptomic transition of individual T cell subsets approaching and leaving this branching onto a heatmap, which allowed us to discover signature gene features that make up this branching point (Figure 4E). A cluster of 13 genes were identified; 11 of 13 of these genes were signature genes identified in the T helper 17 cell (Th17) lineage, including the effector molecule *Il17f* and the master transcription factor *Rorc* (Chang and Dong, 2009; Ciofani et al., 2012; Gobert et al., 2018; Hu et al., 2013; Kim and Lee, 2015; Li et al., 2009; Skepner et al., 2014; Su et al., 2016; Tu et al., 2018; Zhou et al., 2016). These Tc17-like cells may serve as intermediate progenitors to separate T_{RM}s located in the tumor or distant mammary gland mucosa.

To exemplify the differences between tumor or distant T_{RM}s, we selected phenotypic genes that specify distinct T cell stages and portrayed their expression on the pseudotime plot. Genes associated with $T_{Eff/EM}$s such as *Lgals3*, *GzmB*, and *Maf* were preferentially expressed in both $T_{Eff/EM}$s and T_{RM}s within the tumor environment. Reciprocally, genes associated with T_{CM}s such as *Sell* and *Lef1* or *Wnt10a*, which induces anabolic T cell metabolism (Terauchi et al., 2009) and is differentially expressed in long-lived periphery T_{CM}s (Miron et al., 2018), were robustly expressed in distant T_{RM}s (Figure 4F). Notably, when the top 200 genes differentially expressed in the tumor and distant mucosa T_{RM}s were subjected to Gene Ontology analysis, we found that, as we saw in T_{CM}s, a large set of genes comprising the structural content of the ribosome, was highly elevated in the distant T_{RM} population (Figure 4G). In contrast, genes involved in cytokine and chemokine signaling were upregulated in the tumor T_{RM} population, highlighting the inflammatory nature of this population. We concluded that the transcriptional program of tumor T_{RM}s was adapted to the inflammatory nature of the tumor microenvironment. Meanwhile, in the reservoir of the tumor-free mammary mucosa, distant T_{RM}s developed into a quiescent T_{CM}-like phenotype to favor their long-term survival.

CXCR6 expression defines a unique subpopulation of $T_{Eff/EM}$s

We moved to identify the intratumor precursor of distant T_{RM}s within the heterogeneous $T_{Eff/EM}$ pool. We reasoned that these precursor cells should have a distinct chemokine sensing and extracellular matrix rolling profile to facilitate tumor egress. We compiled a list of chemokine receptors and integrins and evaluated their expression. We chose *S1pr1*, a well-known hallmark for T cell tissue egress (Cyster and Schwab, 2012), as a benchmark. Among all of the $T_{Eff/EM}$ populations, *S1pr1* expression was silenced in $T_{Eff/EM}$ p4, suggesting that this may be a population that lacks the potential to egress. In p4, compared to other chemokine receptors, *Cxcr6* was highly expressed; compared to the other 3 populations, p4 was the only population that preferentially upregulated *Cxcr6* (Figure 5A).

The elevated *Cxcr6* expression in p4 was associated with enhanced *Pdcd1* and reduced *IL7r* expression, in direct opposition to p2 (Figure 5B). We virtually sorted out these two

(C and D) Contour (C) and pseudotime (D) analysis of the tumor $T_{Eff/EM}$ populations, tumor T_{RM}s, and distant T_{RM}s shows that these populations distinctly separate by their tissue of origin. The divergence of this lineage path is identified at the branching point.

(E) Heatmap of gene expression of the individual T cell subsets approaching and leaving this branch point. A total of 13 critical genes define this branch point, the majority of which (in red) are signature genes identified in the Th17 lineage.

(F) Surface markers *Lgals3* and *Sell*, functional molecules *GzmB* and *Wnt10a*, and transcription factors *Maf* and *Lef1* were projected back on the pseudotime space to exemplify the differences between tumor T_{RM}s (left column) and distant T_{RM}s (right column)

(G) Gene Ontology analysis reveals that ribosome pathways were enriched in the distant T_{RM} population, while cytokine and chemokine signaling pathways were enriched in tumor T_{RM}s.

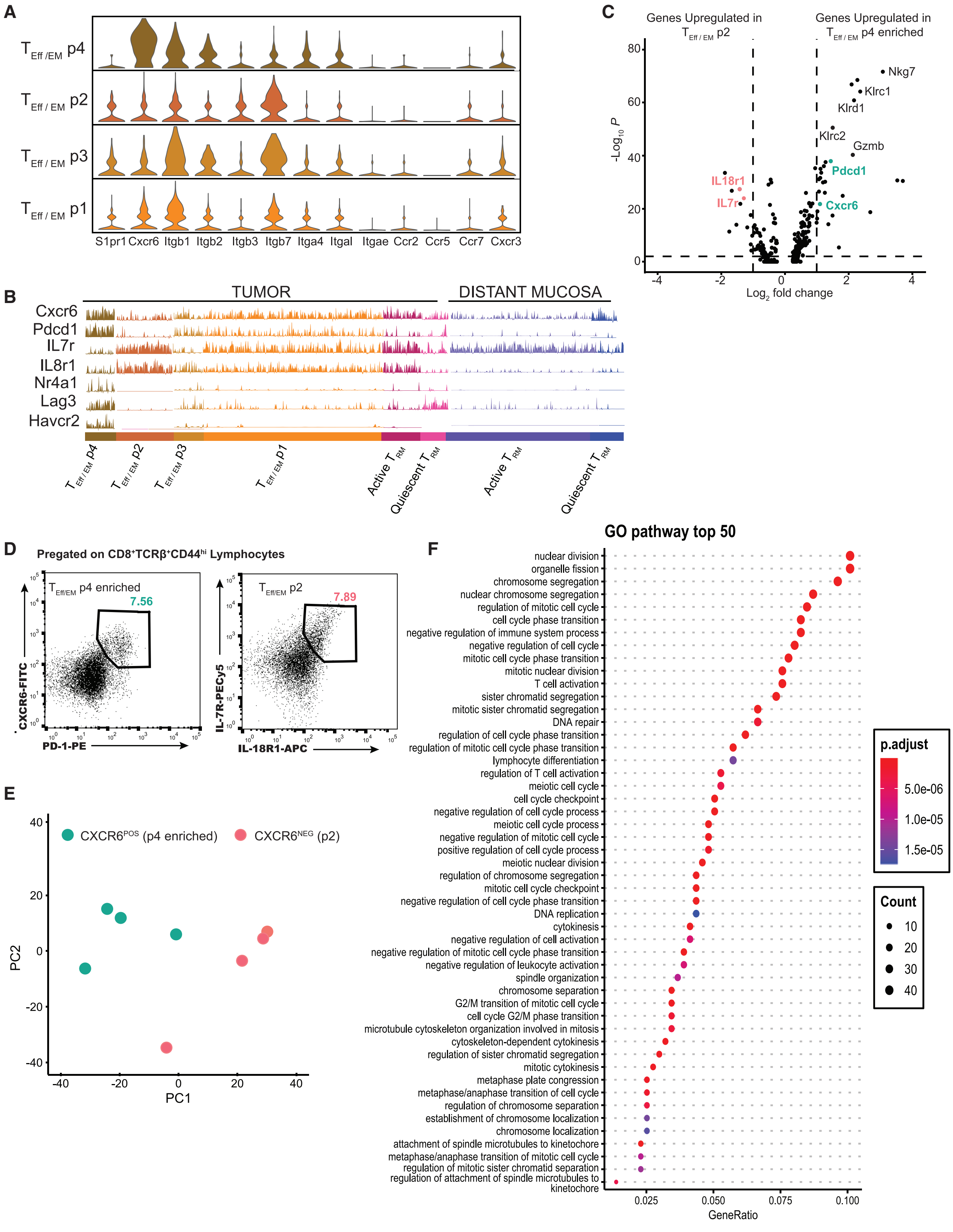

(legend on next page)

populations of cells and compared their gene expression at the transcriptomic level (Figure 5C). The differential expression of *Nkg7* and *Klrc/d* family members suggested that these are highly active effector T cells, as seen in the high expression of effector molecules such as *Gzmb*. However, for T cells in $T_{Eff/EM}$ p2, we found that the upregulation of *IL7r* was associated with *IL18r1* (Figure 5C). For tumor-infiltrating T cells isolated from non-small cell lung cancer samples, IL-18R marks a functional Tbet^{+}Eomes^{+} T_{EM} population (Timperi et al., 2017).

Taking the co-expression of CXCR6 and PD-1 or IL-7R and IL-18R1 as new stage-specific markers, using mice with established 4T1 tumors, we sorted CD44^{hi}CD103^{-}CXCR6^{+}PD-1^{+}$T_{Eff/EM}$ cells, which were highly enriched with the p4 subset (described above) and CD103^{-}IL7R^{+}IL18R1^{+}$T_{Eff/EM}$ cells, which made up the p1 and p2 subsets (Figure 5D) to validate our findings through RNA-seq. Bulk RNA-seq analysis validated that these two populations have distinct transcriptomic profiles (Figure 5E). We directly compared the levels of CXCR6^{+} on tumor T_{RM}s versus $T_{Eff/EM}$s and found that overall, more $T_{Eff/EM}$ cells expressed CXCR6^{+} (Figures S5A and S5B). Flow cytometry analysis validated that CXCR6-expressing $T_{Eff/EM}$ cells were enriched in p4. Within this subpopulation, at the individual cell level, the surface expression of CXCR6 was indistinguishable from that of T_{RM}s (Figures S5C and S5D) (Takamura et al., 2019; Wein et al., 2018). Gene Ontology pathway analysis overwhelmingly showed that the CXCR6^{+} subset of $T_{Eff/EM}$s was actively in the cell cycle, indicating that this population was proliferative (Figure 5F). This analysis illustrated that these CXCR6^{+} effector cells were quite unique: on the one hand, they could be labeled as terminally exhausted cells (Wherry et al., 2007) based on their elevated expression of *Pdcd1*, *Nr4a1* (Liu et al., 2019), *Lag3*, and *Havcr2* (Tim-3); on the other hand, the transcriptional program in these cells overrode all of these possible inhibitions and maintained these cells in the cell cycle with a robust cytolytic program.

CXCR6^{-} $T_{Eff/EM}$s are precursors for distant T_{RM} formation

Since CXCR6 and S1PR1 were reciprocally expressed in $T_{Eff/EM}$ cells, we performed TCR-β repertoire sequencing with the purified $T_{Eff/EM}$ subpopulations to trace the lineage relationship between distinct intratumor $T_{Eff/EM}$ populations and distant T_{RM}s. We sorted tumor CD44^{hi}CD103^{-}CXCR6^{+}PD-1^{+} $T_{Eff/EM}$ cells and CD103^{-}IL7R^{+}IL18R1^{+} $T_{Eff/EM}$ cells, as well as tumor and distant T_{RM}s (CD103^{+}CD69^{+}) for repertoire analysis and clonotype lineage tracing (Figure 6A; Table S3). We compared the repertoire overlap between the purified tumor T_{RM}s and distant T_{RM}s. As previously found in the bulk tissue repertoire analysis (Figure 1I), both high- and low-frequency T_{RM} clonotypes in these two compartments were shared (Figure 6B), supporting that tumor and distant T_{RM}s arose from a common precursor population. In addition, we found that both CXCR6^{-}IL7R^{+}IL18R1^{+} and CXCR6^{+}PD-1^{+} subsets shared TCR clonotypes with tumor T_{RM}s (Figures 6C and 6D). However, for those highly expanded $T_{Eff/EM}$ clones, only the CXCR6^{-} subset contributed to the formation of distant T_{RM}s, while CXCR6^{+} clonotypes barely overlapped with distant T_{RM}s. This suggests that CXCR6 may serve as a retention signal to keep T_{RM} precursors in the tumor.

To validate this tumor-retention mechanism, we sorted CXCR6^{+}PD-1^{+} and IL7R^{+}IL18R1^{+} precursor populations from the tumors of CD45.1^{+} congenically marked BALB/c mice and intratumorally transferred equal numbers (15,000–25,000) into a 4T1 tumor growing in the mammary tissue of Rag2KO BALB/c mice (Figure 6E). Two weeks after transfer, we recovered transferred $T_{Eff/EM}$ precursor cells from the tumor, distant mucosa, and non-draining inguinal lymph node for fluorescence-activated cell sorting (FACS) analysis. We calculated the ratio of cells recovered in the tumor versus the distant mucosa as the readout to minimize experimental variations. The recovered ratios of cells in the tumor versus non-draining lymph node were included as reference. We found that in comparison to CXCR6^{-} cells, the CXCR6^{+} $T_{Eff/EM}$ cells remained in the tumor, validating the preference of the $T_{Eff/EM}$ p4 population to stay in the tumor to become tumor T_{RM}s (Figure 6F).

Breaking CXCR6-mediated retention enhances protection against distant tumor metastasis

Since CXCR6 is the receptor that chemoattracts $T_{Eff/EM}$ cells in the tumor, we predicted that its sole ligand, CXCL16, would be expressed in the tumor tissue. We performed qPCR analysis for *Cxcl16* mRNA with tumor, tumor-adjacent mucosa, distant mucosa, and non-draining lymph node tissues isolated from 4T1 tumor-bearing mice, while the mammary gland mucosa of tumor-naive mice served as a control. Compared to that in mucosal tissues, *Cxcl16* expression in the tumor was significantly stronger (Figure 7A). Furthermore, the relative *Cxcl16* expression level between the distant mucosa from tumor-bearing mice was comparable to that in the tumor-naive mucosa (Figure 7B). Flow cytometry staining confirmed that 4T1 tumor cells can be a direct source of CXCL16 production (Figure 7C). This was further validated by confocal microscopy—within the 4T1 tumor tissues, CXCL16 was almost universally expressed on the surface of 4T1 tumor cells (Figure 7D, best focus view).

Figure 5. CXCR6 defines a unique subpopulation of $T_{Eff/EM}$s

(A) GeneTrac analysis shows expression levels of chemokine receptors and integrins among all tumor $T_{EFF\ /\ TEM}$ subsets. *Cxcr6* shows the highest expression among the $T_{Eff/EM}$ p4 population and correlates with the lowest *S1PR1* expression.

(B) Elevated levels of *Cxcr6* in $T_{Eff/EM}$ p4 are associated with enhanced *Pdcd1* expression, in opposition to *Il7r* and *Il18r1* expression, which are expressed in $T_{Eff/EM}$ p2. Classical exhaustion markers *Nr4a1*, *Lag3*, and *Havcr2* were upregulated in the tumor $T_{Eff/EM}$ p4 population.

(C) Volcano plot of gene expression between tumor $T_{Eff/EM}$ p2 (left) and $T_{Eff/EM}$ p4 (right) shows that T cell activation markers *Gzmb*, *Klrc1/2*, and *Klrd1* are upregulated in $T_{EFF/TEM}$ p4, indicating it is effector like.

(D) Flow cytometry staining on tumor T cells shows protein expression of the 2 $T_{Eff/EM}$ precursor populations, which were sorted for RNA-seq analysis.

(E) PCA analysis confirms transcriptional separation of these 2 populations.

(F) Gene Ontology analysis shows that the CXCR6^{+} population enriches many pathways involving the cell cycle, indicating that this pathway is phenotypically exhausted but highly active.

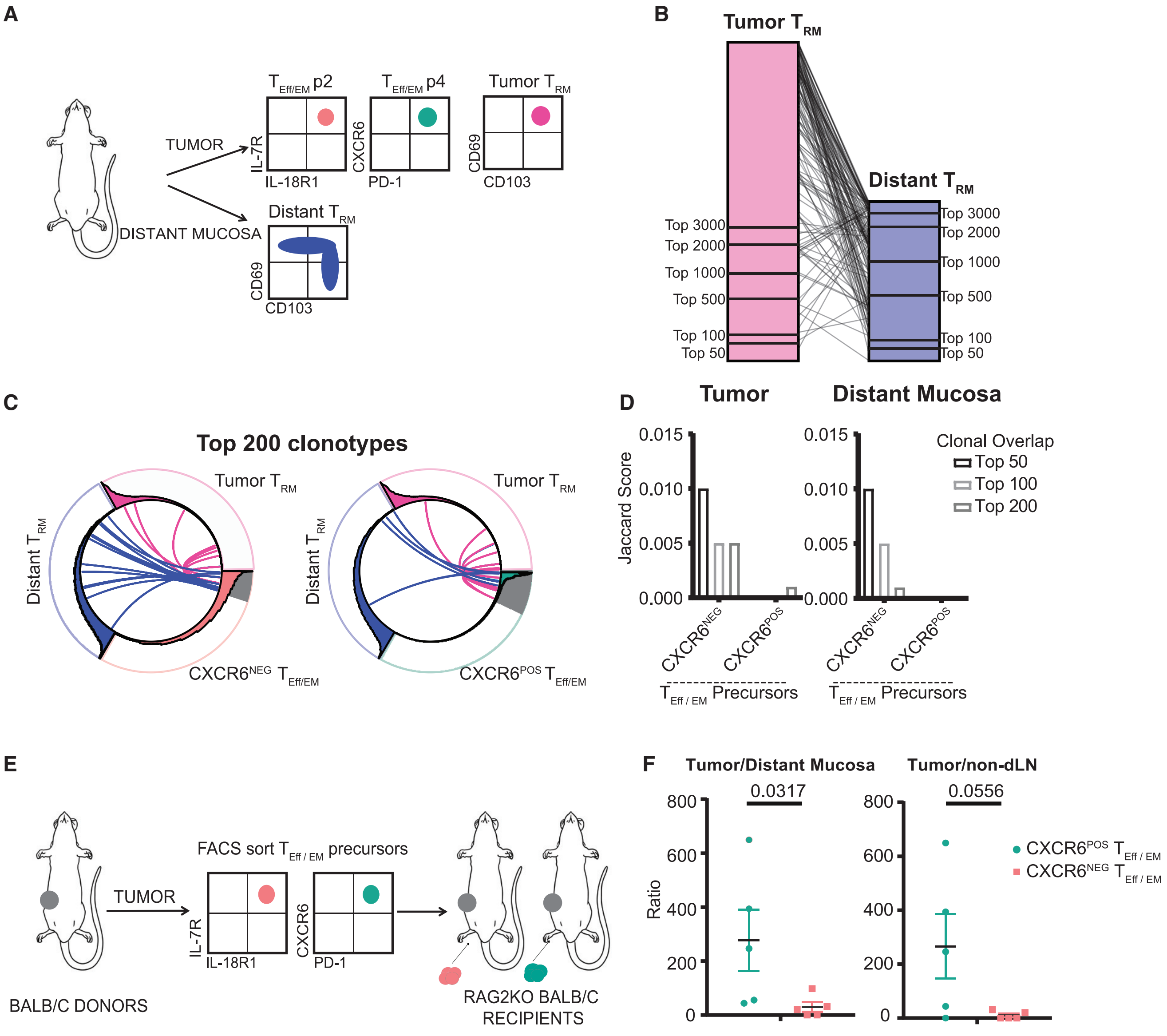

Figure 6. CXCR6$^-$ $T_{Eff/EM}$s are the precursor of distant T_{RM}s

(A) Experimental design of sample collection for TCR-β repertoire sequencing.

(B) Repertoire analysis shows substantial overlap between high and low frequency TCRs between tumor and distant T_{RM}s.

(C and D) Repertoire analysis shows that both CXCR6^{NEG} and CXCR6^{POS} subsets share TCRs with tumor T_{RM}s; only the highly expanded clones in the CXCR6^{NEG} population contributed to distant T_{RM} formation.

(E) Experimental design for $T_{Eff/EM}$ precursor transfer.

(F) Preference of CXCR6$^+$ $T_{Eff/EM}$ precursor cells to stay in the tumor while CXCR6$^-$ $T_{Eff/EM}$ precursors egress to the distant mucosa. Data are represented as a ratio of recovered cells in the tumor divided by the recovered cells in the distant mucosa or LN. Mann-Whitney test, $p < 0.05$. Bars represent means ± SEMs and symbols represent individual mice. Data are pooled over 3 independent experiments.

In addition, produced CXCL16 proteins were deposited on the surrounding extracellular matrix (Figure 7D, bottom plane view). These data indicated that the 4T1 tumor microenvironment can strongly attract CXCR6$^+$ T cells to stay.

We speculated that tumor-produced CXCL16 is a key retention molecule used to curb the residency of tumor-specific T cells in distant tissues. Considering the sentinel functions of T_{RM}s, this retention could be a mechanism exploited by tumors to dampen immunity in distant tissues and facilitate the engraftment of metastases. To test this, at days 7, 14, and 21 after primary tumor inoculation, we intratumorally injected a CXCL16 antibody to neutralize CXCR6 binding (Figure 7E). At day 25, we surgically removed the primary tumor and performed TCR-β repertoire sequencing to characterize the difference in T cell infiltration in the pre-metastatic lung tissue following CXCL16 antibody blocking. The clonotype sharing was increased across

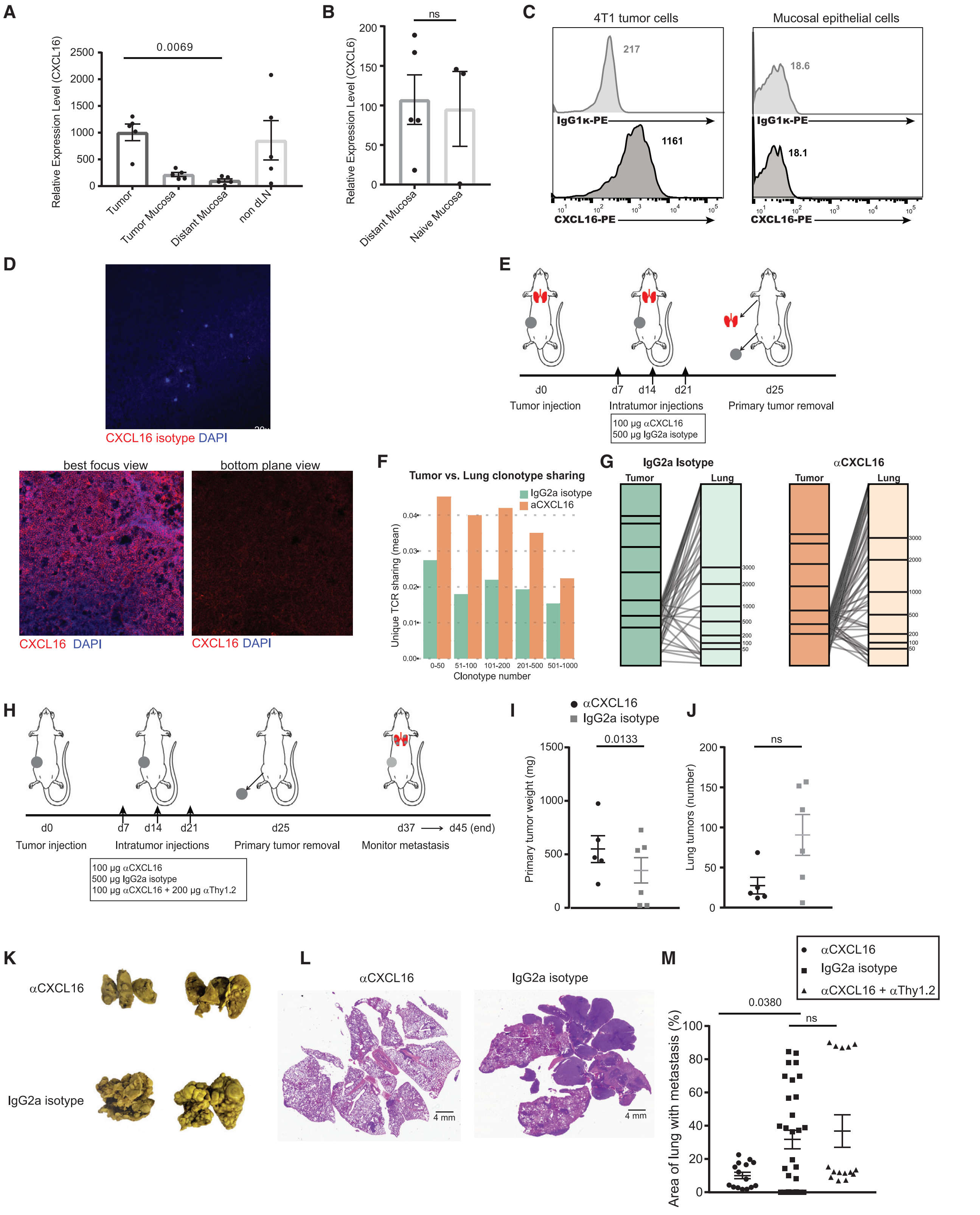

(legend on next page)

T cells in all frequency categories (Figure 7F), and, zooming in on the high-frequency clones, which were likely to be enriched by expanding tumor-specific $T_{Eff/EM}$ cells, the increase was also obvious (Figure 7G). This suggested that breaking CXCR6-mediated retention in the tumor resulted in more T cells egressing to the distant lung tissue.

We next evaluated whether promoting T cell infiltration to the lung could result in enhanced protection against tumor metastasis. Using the same experimental scheme detailed above, we monitored the animals for 2–3 weeks following the removal of the primary tumor (Figure 7H). Anti-CXCL16 treatment caused a moderate difference in the weight of the primary tumors (Figure 7I); at the humane endpoint of individual mice, we assessed the spontaneous 4T1 metastases in the lung. We counted the metastases on the surface of the lung and found that the number of tumor nodules was not statistically different between control IgG and anti-CXCL16 treated mice (Figure 7J). However, we observed striking differences in metastatic tumor burden (Figures 7K and 7L). To prove that this protective mechanism was T cell intrinsic, we depleted T cells with an anti-Thy1.2 antibody, while co-administering mice with anti-CXCL16 (Figure 7H). The anti-CXCL16- enhanced protection was lost when T cells were depleted (Figure 7M). This suggested that breaking CXCL16-CXCR6-mediated T cell retention in primary breast tumors fortifies antimetastatic immunity in the lung.

DISCUSSION

In an orthotopic breast cancer model, we found that T_{RM}s developed in remote mammary gland tissues at early stages of tumorigenesis. TCR repertoire sequencing data revealed that clonotypically, these T_{RM}s were generated in the pre-metastatic stage from the same precursor cells that developed T_{EM}s and T_{RM}s within the tumor (Figure 6B), as well as T_{RM}s in the tumor-adjacent mucosal tissue (Figure 1I). Accordingly, dominant TCR clones in the mammary mucosa were shared among individual tumor-bearing mice. These TCRs were also shared between the tumor and tumor-distant mucosa but were distinct from mammary gland T_{RM}s in tumor-naive mice. scRNA-seq showed that a few tumor T_{EM}s shared a similar transcriptome with active T_{RM}s, although they were sorted based on their classical surface markers. The transcriptomic similarity between tumor T_{EM}s and T_{RM}s echoed the epigenetic similarity recently identified between these two populations in an LCMV infection model, especially for genomic loci that have the most dynamic changes through naive to memory differentiation (Fonseca et al., 2020). This transcriptomic similarity was reinforced by our pseudotime analysis. During the synthetic developmental process, the transcriptomic transition that generated terminally differentiated T_{EM}s was highly similar to the one begetting active T_{RM}s. Pseudotime analysis further showed that although they were sorted from tumors, a few $CD44^{hi}CD62L^{-}CD103^{-}$ cells had already developed a transcriptome resembling that of $CD103^{+}$ T_{RM}s purified from the distant mucosa. These results together suggested that distant mucosa T_{RM}s were generated from $T_{Eff/EM}$ precursors that developed within the tumor. Before gaining their tumor resident credentials, these precursors were able to egress. After they circulated to and infiltrated the distant mammary gland, they found their necessary niche in the local tissue environment, which harbored and supported them to finish their final differentiation to become T_{RM}s in the remote tissue.

The generation of T_{RM}s in remote tissues is a protective mechanism against metastasis. Before metastatic tumor cell invasion, T_{RM}s can establish their defensive perimeters in distant tissues. Distant T_{RM}s function as perfect sentinels. On the one hand, they are derived from $T_{Eff/EM}$ precursors and have tumor antigen specificity, allowing them to detect tumor cells upon their arrival; on the other hand, as memory cells residing in the tissue, they can be reactivated quickly to expedite immune responses locally. These features are especially important for malignancies such as breast cancer, for which the 5-year survival rate in the US is 99% if only localized tumors are found; this rate drops to 27% if there are distant metastases (National Cancer Institute Surveillance, Epidemiology, and End Results [NCI SEER] program). While the importance of T_{RM}s in various types of epithelial-associated cancers has recently emerged (Amsen et al., 2018), their exact antitumor roles are not characterized. Our clinical study on early-diagnosed patients with local gastric cancer showed that their 4-year prognosis was associated with the clonal diversity of T_{RM}s in the tumor-adjacent mucosa rather than the diversity of T cells in the tumor or peripheral blood. The majority of the mortality in this cohort was the result of stomach cancer relapse

Figure 7. Breaking CXCL16/CXCR6 retention enhances protection against distant lung tumor metastasis
(A) qPCR for the CXCR6 ligand *Cxcl16* shows highest expression in the tumor.
(B) *Cxcl16* mRNA is similar in the distant mucosa from tumor-bearing mice compared to the mucosa from tumor-naive mice. Two-way ANOVA with Kruskal-Wallis test, $p < 0.005$. Bars represent means ± SEMs and symbols represent individual mice.
(C) Flow cytometry validates CXCL16 expression on the surface of 4T1 tumor cells but not mucosal epithelial cells.
(D) Confocal microscopy characterizes CXCL16 expression within the 4T1 primary tumor.
(E) Experimental design for sample collection in the presence or absence of anti-CXCL16 blocking.
(F and G) Global TCR-β repertoire sequencing analysis compares clonotype sharing between the tumor and the pre-metastatic lung in the presence or absence of anti-CXCL16 blocking. These data are represented in all frequency categories (F) and by zooming into the top 200 high frequency clones (G).
(H) Experimental procedure to monitor spontaneous lung metastasis after surgical removal of the primary tumor in the presence or absence of anti-CXCL16 treatment.
(I) Analysis of the primary tumors. Bars represent means ± SEMs and symbols represent individual mice. Paired t test, $p < 0.05$.
(J) Analysis of the number of metastatic tumor nodules.
(K) Representative images of metastatic tumor lungs.
(L) Representative H&E staining of anti-CXCL16 and isotype-treated lungs showing the area of the lung harboring tumor metastases (dark purple).
(M) Quantification of metastatic tumor occupancy in the lung, performed by a third party in a 1-sided blinded manner. Data were measured by diving the area of tumor metastases by total lung area and represented as a percentage. Paired t test, $p < 0.05$. Bars represent means ± SEMs and symbols represent individual lung tissue sections. Data represent 2 individual experiments combined.

or liver or peritoneum metastasis (Jia et al., 2015). Although the distant mucosal tissues of those patients could not be surveyed, based on the repertoire similarity between the tumor-adjacent and tumor-distant mucosa, we speculated that the correlation between prognosis and T_{RM} diversity may reflect the unique immunosurveillance function of extratumoral T_{RM}s. Their protective functions go beyond the primary tumor and are specialized against tumor recurrence.

To approach the retention mechanism separating T cells that remain in the tumor versus those that leave to travel to remote tissues, we identified CXCR6 as a key player. CXCR6 was proposed to be the chemokine receptor driving flu-specific T_{RM}s to reside in the airway epithelium, while CXCR6$^-$ T_{RM}s stayed in the interstitium (Wein et al., 2018). In the same influenza infection model, fully differentiated CXCR6$^+$ T_{RM}s in the interstitium replenished the airway compartment, not circulating T_{EM}s (Ely et al., 2006; Takamura et al., 2019; Zammit et al., 2006). Both studies clearly demonstrated that airway CXCL16 production and T_{RM} CXCR6 expression were necessary for airway T_{RM}s to form. The present study revealed a similar chemoattraction mechanism trapping effector T cells in the tumor. The heightened expression of CXCL16 by tumor cells is a common characteristic found in tissue from gastric (Xing et al., 2012) and colon carcinoma patients (Hojo et al., 2007), as well as in various mammary carcinoma cell lines (Meijer et al., 2011). This differential expression was also observed in the 4T1 tumor versus the distant mammary gland mucosa or the mucosa from tumor-naive mice. We showed that transferred CXCR6$^+$ tumor $T_{Eff/EM}$s were more likely to be trapped in the recipient tumor than their CXCR6$^-$ counterparts. Furthermore, in a snapshot of TCR repertoire analysis, more tumor CXCR6$^-$ $T_{Eff/EM}$ TCRs overlapped with T_{RM}s isolated from the distant mucosa than CXCR6$^+$ $T_{Eff/EM}$s in the tumor. We reasoned that in this 4T1 model, tumor cells served as the source of CXCL16 to retain tumor-infiltrating $T_{Eff/EM}$ cells. Our repertoire analysis also showed that TCRs from CXCR6$^-$ and CXCR6$^+$ subsets had significant overlap. Whether CXCR6 expression is stochastic or how it is regulated in tumor $T_{Eff/EM}$ cells is still not clear. However, our scRNA-seq and FACS data demonstrated a strong correlation between CXCR6 and PD-1 expression, which suggests that it could be related to tumor antigen stimulation.

Our study also showed that T_{RM}s, especially T_{RM}s in the distant mammary gland, divided into two major populations based on their ribosome-associated gene expression. The elevated expression of these genes was closely related to quiescent features of their transcriptome. This association has been revealed in CD8$^+$ memory T cell development during LCMV infection. In this model, both ribosomal protein mRNAs and overall translation activities are drastically decreased when T cells enter the phase of terminal effector differentiation. Ribosomal protein mRNA expression in T_{Eff}s was lower than that in memory precursor (T_{MP}) cells, and their expression in T_{MP}s was lower than in quiescent naive T cells (Araki et al., 2017). Furthermore, suppressed ribosomal protein mRNA expression depended on antigen stimulation and mammalian target of rapamycin complex 1 (mTORC1) activity. The mTORC1 inhibitor rapamycin was shown to promote long-lived T_{CM} formation (Araki et al., 2010). We speculated that this mechanism could be directly applied to T_{RM}s. Like LCMV clone 13-induced exhaustion, chronic tumor antigen stimulation may suppress ribosomal protein mRNA expression. Consequently, T_{RM}s found in the distant mucosa are isolated from tumor antigens and thus returned to a quiescent stage to cope with their longevity. However, this model cannot fully explain how T_{CM}s found in the tumor microenvironment maintain their robust ribosomal protein mRNA expression or the evolutionary factors that keep these mRNA levels abundant in quiescent cells, both of which are subjects for future study.

STAR★METHODS

Detailed methods are provided in the online version of this paper and include the following:

- KEY RESOURCES TABLE
- RESOURCE AVAILABILITY
  - Lead contact
  - Materials availability
  - Data and code availability
- EXPERIMENTAL MODEL AND SUBJECT DETAILS
  - Animals
  - Cell lines
- METHOD DETAILS
  - Tumor model and tissue isolation
  - Tumor T_{RM} characterization
  - TCRβ repertoire sequencing
  - TCRβ repertoire analysis
  - 10x Genomics library preparation and sequencing
  - Single-cell RNA-seq analysis
  - Bulk RNA-sequencing
  - Bulk RNA-sequencing analysis
  - Adoptive transfer of T_{EM} precursor cells
  - CXCL16 qPCR
  - CXCL16 flow cytometry
  - Confocal microscopy
  - Antibody blocking experiments, lung H&E analysis
- QUANTIFICATION AND STATISTICAL ANALYSIS

SUPPLEMENTAL INFORMATION

Supplemental information can be found online at https://doi.org/10.1016/j.celrep.2021.109118.

ACKNOWLEDGMENTS

We would like to acknowledge the Duke Molecular Physiology Institute Molecular Genomics core for assisting in the generation of data for the manuscript. We thank Drs. Diyuan Qin, Tao Yin, and Guoping Wang for their pathological assessment and quantification of lung metastases. This work was supported by the NIH grants R33CA225325 (to Q.-J.L.) and R01CA233205 and R01CA225622 (to X.-F.W. and Q.-J.L.).

AUTHOR CONTRIBUTIONS

L.S.C. and Q.-J.L. designed the experiments; L.S.C. performed the majority of the experiments; B.L. assisted in the experiments; D.D. and H.W. performed the TCR repertoire sequencing; L.W. performed all of the bioinformatics analysis and created the data visualization; L.S.C. and Q.-J.L. wrote the manuscript; Q.-J.L. and X.-F.W. formulated this study and oversaw the project.

DECLARATION OF INTERESTS

Q.J.L. is a scientific co-founder and shareholder of TCRCure Biopharma. Q.J.L. and X.F.W. are the scientific advisory board members of TCRCure Biopharma. Q.-J.L., X.-F.W., L.S.C., and L.W. are co-inventors of a provisional patent (#63/157,015) through Duke University.

Received: September 29, 2020
Revised: February 15, 2021
Accepted: April 21, 2021
Published: May 11, 2021

REFERENCES

Aibar, S., González-Blas, C.B., Moerman, T., Huynh-Thu, V.A., Imrichova, H., Hulselmans, G., Rambow, F., Marine, J.-C., Geurts, P., Aerts, J., et al. (2017). SCENIC: single-cell regulatory network inference and clustering. Nat. Methods *14*, 1083–1086.

Andrews, S. (2010). FastQC: a quality control tool for high throughput sequence data from. http://www.bioinformatics.babraham.ac.uk/projects/fastqc.

Amsen, D., van Gisbergen, K.P.J.M., Hombrink, P., and van Lier, R.A.W. (2018). Tissue-resident memory T cells at the center of immunity to solid tumors. Nat. Immunol. *19*, 538–546.

Araki, K., Youngblood, B., and Ahmed, R. (2010). The role of mTOR in memory CD8 T-cell differentiation. Immunol. Rev. *235*, 234–243.

Araki, K., Morita, M., Bederman, A.G., Konieczny, B.T., Kissick, H.T., Sonenberg, N., and Ahmed, R. (2017). Translation is actively regulated during the differentiation of $CD8^+$ effector T cells. Nat. Immunol. *18*, 1046–1057.

Ariotti, S., Hogenbirk, M.A., Dijkgraaf, F.E., Visser, L.L., Hoekstra, M.E., Song, J.Y., Jacobs, H., Haanen, J.B., and Schumacher, T.N. (2014). T cell memory. Skin-resident memory $CD8^+$ T cells trigger a state of tissue-wide pathogen alert. Science *346*, 101–105.

Best, J.A., Blair, D.A., Knell, J., Yang, E., Mayya, V., Doedens, A., Dustin, M.L., and Goldrath, A.W.; Immunological Genome Project Consortium (2013). Transcriptional insights into the CD8(+) T cell response to infection and memory T cell formation. Nat. Immunol. *14*, 404–412.

Bolotin, D.A., Poslavsky, S., Mitrophanov, I., Shugay, M., Mamedov, I.Z., Putintseva, E.V., and Chudakov, D.M. (2015). MiXCR: software for comprehensive adaptive immunity profiling. Nat. Methods *12*, 380–381.

Buenrostro, J.D., Corces, M.R., Lareau, C.A., Wu, B., Schep, A.N., Aryee, M.J., Majeti, R., Chang, H.Y., and Greenleaf, W.J. (2018). Integrated Single-Cell Analysis Maps the Continuous Regulatory Landscape of Human Hematopoietic Differentiation. Cell *173*, 1535–1548.e16.

Butler, A., Hoffman, P., Smibert, P., Papalexi, E., and Satija, R. (2018). Integrating single-cell transcriptomic data across different conditions, technologies, and species. Nat. Biotechnol. *36*, 411–420.

Cannarile, M.A., Lind, N.A., Rivera, R., Sheridan, A.D., Camfield, K.A., Wu, B.B., Cheung, K.P., Ding, Z., and Goldrath, A.W. (2006). Transcriptional regulator Id2 mediates CD8+ T cell immunity. Nat. Immunol. *7*, 1317–1325.

Cepek, K.L., Shaw, S.K., Parker, C.M., Russell, G.J., Morrow, J.S., Rimm, D.L., and Brenner, M.B. (1994). Adhesion between epithelial cells and T lymphocytes mediated by E-cadherin and the alpha E beta 7 integrin. Nature *372*, 190–193.

Chan, C.P., Kok, K.H., and Jin, D.Y. (2011). CREB3 subfamily transcription factors are not created equal: Recent insights from global analyses and animal models. Cell Biosci. *1*, 6.

Chang, S.H., and Dong, C. (2009). IL-17F: regulation, signaling and function in inflammation. Cytokine *46*, 7–11.

Chen, S., Zhou, Y., Chen, Y., and Gu, J. (2018). fastp: an ultra-fast all-in-one FASTQ preprocessor. Bioinformatics *34*, i884–i890.

Ciofani, M., Madar, A., Galan, C., Sellars, M., Mace, K., Pauli, F., Agarwal, A., Huang, W., Parkhurst, C.N., Muratet, M., et al. (2012). A validated regulatory network for Th17 cell specification. Cell *151*, 289–303.

Cruz-Guilloty, F., Pipkin, M.E., Djuretic, I.M., Levanon, D., Lotem, J., Lichtenheld, M.G., Groner, Y., and Rao, A. (2009). Runx3 and T-box proteins cooperate to establish the transcriptional program of effector CTLs. J. Exp. Med. *206*, 51–59.

Cyster, J.G., and Schwab, S.R. (2012). Sphingosine-1-phosphate and lymphocyte egress from lymphoid organs. Annu. Rev. Immunol. *30*, 69–94.

Djenidi, F., Adam, J., Goubar, A., Durgeau, A., Meurice, G., de Montpréville, V., Validire, P., Besse, B., and Mami-Chouaib, F. (2015). CD8+CD103+ tumor-infiltrating lymphocytes are tumor-specific tissue-resident memory T cells and a prognostic factor for survival in lung cancer patients. J. Immunol. *194*, 3475–3486.

Dobin, A., Davis, C.A., Schlesinger, F., Drenkow, J., Zaleski, C., Jha, S., Batut, P., Chaisson, M., and Gingeras, T.R. (2013). STAR: ultrafast universal RNA-seq aligner. Bioinformatics *29*, 15–21.

Ely, K.H., Cookenham, T., Roberts, A.D., and Woodland, D.L. (2006). Memory T cell populations in the lung airways are maintained by continual recruitment. J. Immunol. *176*, 537–543.

Enamorado, M., Iborra, S., Priego, E., Cueto, F.J., Quintana, J.A., Martínez-Cano, S., Mejías-Pérez, E., Esteban, M., Melero, I., Hidalgo, A., and Sancho, D. (2017). Enhanced anti-tumour immunity requires the interplay between resident and circulating memory $CD8^+$ T cells. Nat. Commun. *8*, 16073.

Feng, X., Ippolito, G.C., Tian, L., Wiehagen, K., Oh, S., Sambandam, A., Willen, J., Bunte, R.M., Maika, S.D., Harriss, J.V., et al. (2010). Foxp1 is an essential transcriptional regulator for the generation of quiescent naive T cells during thymocyte development. Blood *115*, 510–518.

Fonseca, R., Beura, L.K., Quarnstrom, C.F., Ghoneim, H.E., Fan, Y., Zebley, C.C., Scott, M.C., Fares-Frederickson, N.J., Wijeyesinghe, S., Thompson, E.A., et al. (2020). Developmental plasticity allows outside-in immune responses by resident memory T cells. Nat. Immunol. *21*, 412–421.

Fuhlbrigge, R.C., Kieffer, J.D., Armerding, D., and Kupper, T.S. (1997). Cutaneous lymphocyte antigen is a specialized form of PSGL-1 expressed on skin-homing T cells. Nature *389*, 978–981.

Gaide, O., Emerson, R.O., Jiang, X., Gulati, N., Nizza, S., Desmarais, C., Robins, H., Krueger, J.G., Clark, R.A., and Kupper, T.S. (2015). Common clonal origin of central and resident memory T cells following skin immunization. Nat. Med. *21*, 647–653.

Gerlach, C., Moseman, E.A., Loughhead, S.M., Alvarez, D., Zwijnenburg, A.J., Waanders, L., Garg, R., de la Torre, J.C., and von Andrian, U.H. (2016). The Chemokine Receptor CX3CR1 Defines Three Antigen-Experienced CD8 T Cell Subsets with Distinct Roles in Immune Surveillance and Homeostasis. Immunity *45*, 1270–1284.

Gobert, A.P., Al-Greene, N.T., Singh, K., Coburn, L.A., Sierra, J.C., Verriere, T.G., Luis, P.B., Schneider, C., Asim, M., Allaman, M.M., et al. (2018). Distinct Immunomodulatory Effects of Spermine Oxidase in Colitis Induced by Epithelial Injury or Infection. Front. Immunol. *9*, 1242.

Herndler-Brandstetter, D., Ishigame, H., Shinnakasu, R., Plajer, V., Stecher, C., Zhao, J., Lietzenmayer, M., Kroehling, L., Takumi, A., Kometani, K., et al. (2018). $KLRG1^+$ Effector $CD8^+$ T Cells Lose KLRG1, Differentiate into All Memory T Cell Lineages, and Convey Enhanced Protective Immunity. Immunity *48*, 716–729.e8.

Hojo, S., Koizumi, K., Tsuneyama, K., Arita, Y., Cui, Z., Shinohara, K., Minami, T., Hashimoto, I., Nakayama, T., Sakurai, H., et al. (2007). High-level expression of chemokine CXCL16 by tumor cells correlates with a good prognosis and increased tumor-infiltrating lymphocytes in colorectal cancer. Cancer Res. *67*, 4725–4731.

Hu, R., Huffaker, T.B., Kagele, D.A., Runtsch, M.C., Bake, E., Chaudhuri, A.A., Round, J.L., and O'Connell, R.M. (2013). MicroRNA-155 confers encephalogenic potential to Th17 cells by promoting effector gene expression. J. Ilmmunol. *190*, 5972–5980.

Iijima, N., and Iwasaki, A. (2015). Tissue instruction for migration and retention of TRM cells. Trends Immunol. *36*, 556–564.

Intlekofer, A.M., Takemoto, N., Wherry, E.J., Longworth, S.A., Northrup, J.T., Palanivel, V.R., Mullen, A.C., Gasink, C.R., Kaech, S.M., Miller, J.D., et al.

(2005). Effector and memory CD8+ T cell fate coupled by T-bet and eomesodermin. Nat. Immunol. *6*, 1236–1244.

Jia, Q., Zhou, J., Chen, G., Shi, Y., Yu, H., Guan, P., Lin, R., Jiang, N., Yu, P., Li, Q.J., and Wan, Y. (2015). Diversity index of mucosal resident T lymphocyte repertoire predicts clinical prognosis in gastric cancer. OncoImmunology *4*, e1001230.

Jiang, X., Clark, R.A., Liu, L., Wagers, A.J., Fuhlbrigge, R.C., and Kupper, T.S. (2012). Skin infection generates non-migratory memory CD8+ T(RM) cells providing global skin immunity. Nature *483*, 227–231.

Joo, H.-G., Goedegebuure, P.S., Sadanaga, N., Nagoshi, M., von Bernstorff, W., and Eberlein, T.J. (2001). Expression and function of galectin-3, a β-galactoside-binding protein in activated T lymphocytes. J. Leukoc. Biol. *69*, 555–564.

Kallies, A., Xin, A., Belz, G.T., and Nutt, S.L. (2009). Blimp-1 transcription factor is required for the differentiation of effector CD8(+) T cells and memory responses. Immunity *31*, 283–295.

Kashiwada, M., Cassel, S.L., Colgan, J.D., and Rothman, P.B. (2011). NFIL3/E4BP4 controls type 2 T helper cell cytokine expression. EMBO J. *30*, 2071–2082.

Kim, H.S., and Lee, G. (2015). The cysteinyl leukotriene receptor CysLTR1 mediates Th17 cell migration (CAM4P.156). J. Immunol. *194*, 185.114.

Kobak, D., and Berens, P. (2019). The art of using t-SNE for single-cell transcriptomics. Nat. Commun. *10*, 5416.

Kouo, T., Huang, L., Pucsek, A.B., Cao, M., Solt, S., Armstrong, T., and Jaffee, E. (2015). Galectin-3 Shapes Antitumor Immune Responses by Suppressing CD8+ T Cells via LAG-3 and Inhibiting Expansion of Plasmacytoid Dendritic Cells. Cancer Immunol. Res. *3*, 412–423.

Kurd, N.S., He, Z., Milner, J.J., Omilusik, K.D., Louis, T.L., Tsai, M.S., Widjaja, C.E., Kanbar, J.N., Olvera, J.G., Tysl, T., et al. (2020). Molecular determinants and heterogeneity of tissue-resident memory $CD8^+$ T lymphocytes revealed by single-cell RNA sequencing. bioRxiv, 2020.2003.2002.973578.

Li, B., Reynolds, J.M., Stout, R.D., Bernlohr, D.A., and Suttles, J. (2009). Regulation of Th17 differentiation by epidermal fatty acid-binding protein. J. Immunol. *182*, 7625–7633.

Liao, Y., Smyth, G.K., and Shi, W. (2014). featureCounts: an efficient general purpose program for assigning sequence reads to genomic features. Bioinformatics *30*, 923–930.

Liu, X., Wang, Y., Lu, H., Li, J., Yan, X., Xiao, M., Hao, J., Alekseev, A., Khong, H., Chen, T., et al. (2019). Genome-wide analysis identifies NR4A1 as a key mediator of T cell dysfunction. Nature *567*, 525–529.

Love, M.I., Huber, W., and Anders, S. (2014). Moderated estimation of fold change and dispersion for RNA-seq data with DESeq2. Genome Biol. *15*, 550.

Luo, C.T., Osmanbeyoglu, H.U., Do, M.H., Bivona, M.R., Toure, A., Kang, D., Xie, Y., Leslie, C.S., and Li, M.O. (2017). Ets transcription factor GABP controls T cell homeostasis and immunity. Nat. Commun. *8*, 1062.

Mackay, C.R., Marston, W.L., Dudler, L., Spertini, O., Tedder, T.F., and Hein, W.R. (1992). Tissue-specific migration pathways by phenotypically distinct subpopulations of memory T cells. Eur. J. Immunol. *22*, 887–895.

Malik, B.T., Byrne, K.T., Vella, J.L., Zhang, P., Shabaneh, T.B., Steinberg, S.M., Molodtsov, A.K., Bowers, J.S., Angeles, C.V., Paulos, C.M., et al. (2017). Resident memory T cells in the skin mediate durable immunity to melanoma. Sci. Immunol. *2*, eaam6346.

Masopust, D., Vezys, V., Marzo, A.L., and Lefrançois, L. (2001). Preferential localization of effector memory cells in nonlymphoid tissue. Science *291*, 2413–2417.

Meijer, J., Ogink, J., Kreike, B., Nuyten, D., de Visser, K.E., and Roos, E. (2011). Retraction. Cancer Res. *71*, 1196.

Milner, J.J., Toma, C., He, Z., Kurd, N.S., Nguyen, Q.P., McDonald, B., Quezada, L., Widjaja, C.E., Witherden, D.A., Crowl, J.T., et al. (2020). Heterogenous Populations of Tissue-Resident $CD8^+$ T Cells Are Generated in Response to Infection and Malignancy. Immunity *52*, 808–824.e7.

Miron, M., Kumar, B.V., Meng, W., Granot, T., Carpenter, D.J., Senda, T., Chen, D., Rosenfeld, A.M., Zhang, B., Lerner, H., et al. (2018). Human Lymph Nodes Maintain TCF-1^{hi} Memory T Cells with High Functional Potential and Clonal Diversity throughout Life. J. Immunol. *201*, 2132–2140.

Nazarov, V.I., Pogorelyy, M.V., Komech, E.A., Zvyagin, I.V., Bolotin, D.A., Shugay, M., Chudakov, D.M., Lebedev, Y.B., and Mamedov, I.Z. (2015). tcR: an R package for T cell receptor repertoire advanced data analysis. BMC Bioinformatics *16*, 175.

Park, S.L., Buzzai, A., Rautela, J., Hor, J.L., Hochheiser, K., Effern, M., McBain, N., Wagner, T., Edwards, J., McConville, R., et al. (2019). Tissue-resident memory CD8(+) T cells promote melanoma-immune equilibrium in skin. Nature *565*, 366–371.

Pearce, E.L., Mullen, A.C., Martins, G.A., Krawczyk, C.M., Hutchins, A.S., Zediak, V.P., Banica, M., DiCioccio, C.B., Gross, D.A., Mao, C.A., et al. (2003). Control of effector CD8+ T cell function by the transcription factor Eomesodermin. Science *302*, 1041–1043.

Qiu, X., Mao, Q., Tang, Y., Wang, L., Chawla, R., Pliner, H.A., and Trapnell, C. (2017). Reversed graph embedding resolves complex single-cell trajectories. Nat. Methods *14*, 979–982.

Sallusto, F., Lenig, D., Förster, R., Lipp, M., and Lanzavecchia, A. (1999). Two subsets of memory T lymphocytes with distinct homing potentials and effector functions. Nature *401*, 708–712.

Savas, P., Virassamy, B., Ye, C., Salim, A., Mintoff, C.P., Caramia, F., Salgado, R., Byrne, D.J., Teo, Z.L., Dushyanthen, S., et al.; Kathleen Cuningham Foundation Consortium for Research into Familial Breast Cancer (kConFab) (2018). Single-cell profiling of breast cancer T cells reveals a tissue-resident memory subset associated with improved prognosis. Nat. Med. *24*, 986–993.

Schenkel, J.M., and Masopust, D. (2014). Tissue-resident memory T cells. Immunity *41*, 886–897.

Schneider, C.A., Rasband, W.S., and Eliceiri, K.W. (2012). NIH Image to ImageJ: 25 years of image analysis. Nat. Methods *9*, 671–675.

Shin, H.M., Kapoor, V., Guan, T., Kaech, S.M., Welsh, R.M., and Berg, L.J. (2013). Epigenetic modifications induced by Blimp-1 Regulate $CD8^+$ T cell memory progression during acute virus infection. Immunity *39*, 661–675.

Sircar, P., Furr, K.L., Dorosh, L.A., and Letvin, N.L. (2010). Clonal repertoires of virus-specific CD8+ T lymphocytes are shared in mucosal and systemic compartments during chronic simian immunodeficiency virus infection in rhesus monkeys. J. Immunol. *185*, 2191–2199.

Skepner, J., Ramesh, R., Trocha, M., Schmidt, D., Baloglu, E., Lobera, M., Carlson, T., Hill, J., Orband-Miller, L.A., Barnes, A., et al. (2014). Pharmacologic inhibition of RORγt regulates Th17 signature gene expression and suppresses cutaneous inflammation in vivo. J. Immunol. *192*, 2564–2575.

Slütter, B., Van Braeckel-Budimir, N., Abboud, G., Varga, S.M., Salek-Ardakani, S., and Harty, J.T. (2017). Dynamics of influenza-induced lung-resident memory T cells underlie waning heterosubtypic immunity. Sci. Immunol. *2*, eaag2031.

Su, P., Chen, S., Zheng, Y.H., Zhou, H.Y., Yan, C.H., Yu, F., Zhang, Y.G., He, L., Zhang, Y., Wang, Y., et al. (2016). Novel Function of Extracellular Matrix Protein 1 in Suppressing Th17 Cell Development in Experimental Autoimmune Encephalomyelitis. J. Immunol. *197*, 1054–1064.

Suo, S., Zhu, Q., Saadatpour, A., Fei, L., Guo, G., and Yuan, G.C. (2018). Revealing the Critical Regulators of Cell Identity in the Mouse Cell Atlas. Cell Rep. *25*, 1436–1445.e3.

Takamura, S., Kato, S., Motozono, C., Shimaoka, T., Ueha, S., Matsuo, K., Miyauchi, K., Masumoto, T., Katsushima, A., Nakayama, T., et al. (2019). Interstitial-resident memory $CD8^+$ T cells sustain frontline epithelial memory in the lung. J. Exp. Med. *216*, 2736–2747.

Teijaro, J.R., Turner, D., Pham, Q., Wherry, E.J., Lefrançois, L., and Farber, D.L. (2011). Cutting edge: tissue-retentive lung memory CD4 T cells mediate optimal protection to respiratory virus infection. J. Immunol. *187*, 5510–5514.

Terauchi, M., Li, J.Y., Bedi, B., Baek, K.H., Tawfeek, H., Galley, S., Gilbert, L., Nanes, M.S., Zayzafoon, M., Guldberg, R., et al. (2009). T lymphocytes amplify

the anabolic activity of parathyroid hormone through Wnt10b signaling. Cell Metab. *10*, 229–240.

Timperi, E., Focaccetti, C., Gallerano, D., Panetta, M., Spada, S., Gallo, E., Visca, P., Venuta, F., Diso, D., Prelaj, A., et al. (2017). IL-18 receptor marks functional CD8^{+} T cells in non-small cell lung cancer. OncoImmunology *6*, e1328337.

Trapnell, C., Cacchiarelli, D., Grimsby, J., Pokharel, P., Li, S., Morse, M., Lennon, N.J., Livak, K.J., Mikkelsen, T.S., and Rinn, J.L. (2014). The dynamics and regulators of cell fate decisions are revealed by pseudotemporal ordering of single cells. Nat. Biotechnol. *32*, 381–386.

Tu, Z., Zhang, S., Zhou, G., Zhou, L., Xiang, Q., Chen, Q., Zhao, P., Zhan, H., Zhou, H., and Sun, L. (2018). LMO4 Is a Disease-Provocative Transcription Coregulator Activated by IL-23 in Psoriatic Keratinocytes. J. Invest. Dermatol. *138*, 1078–1087.

Unsoeld, H., Krautwald, S., Voehringer, D., Kunzendorf, U., and Pircher, H. (2002). Cutting edge: CCR7+ and CCR7- memory T cells do not differ in immediate effector cell function. J. Immunol. *169*, 638–641.

van Lier, R.A., Borst, J., Vroom, T.M., Klein, H., Van Mourik, P., Zeijlemaker, W.P., and Melief, C.J. (1987). Tissue distribution and biochemical and functional properties of Tp55 (CD27), a novel T cell differentiation antigen. J. Immunol. *139*, 1589–1596.

Venturi, V., Kedzierska, K., Tanaka, M.M., Turner, S.J., Doherty, P.C., and Davenport, M.P. (2008). Method for assessing the similarity between subsets of the T cell receptor repertoire. J. Immunol. Methods *329*, 67–80.

von Andrian, U.H., and Mackay, C.R. (2000). T-cell function and migration. Two sides of the same coin. N. Engl. J. Med. *343*, 1020–1034.

Wang, Z.-Q., Milne, K., Derocher, H., Webb, J.R., Nelson, B.H., and Watson, P.H. (2016). CD103 and Intratumoral Immune Response in Breast Cancer. Clin. Cancer Res. *22*, 6290–6297.

Webb, J.R., Milne, K., Watson, P., Deleeuw, R.J., and Nelson, B.H. (2014). Tumor-infiltrating lymphocytes expressing the tissue resident memory marker CD103 are associated with increased survival in high-grade serous ovarian cancer. Clin. Cancer Res. *20*, 434–444.

Wein, A.N., McMaster, S.R., Takamura, S., Dunbar, P.R., Cartwright, E.K., Hayward, S.L., McManus, D.T., Shimaoka, T., Ueha, S., Tsukui, T., et al. (2018). CXCR6 regulates localization of tissue-resident memory CD8 T cells to the airways. J. Exp. Med. *216*, 2748–2762.

Wherry, E.J., Ha, S.-J., Kaech, S.M., Haining, W.N., Sarkar, S., Kalia, V., Subramaniam, S., Blattman, J.N., Barber, D.L., and Ahmed, R. (2007). Molecular signature of CD8+ T cell exhaustion during chronic viral infection. Immunity *27*, 670–684.

Wickham, H. (2016). ggplot2: Elegant Graphics for Data Analysis (Springer-Verlag: New York).

Williams, M.A., and Bevan, M.J. (2007). Effector and memory CTL differentiation. Annu. Rev. Immunol. *25*, 171–192.

Wolf, F.A., Angerer, P., and Theis, F.J. (2018). SCANPY: large-scale single-cell gene expression data analysis. Genome Biol. *19*, 15.

Wu, T., Hu, Y., Lee, Y.T., Bouchard, K.R., Benechet, A., Khanna, K., and Cauley, L.S. (2014). Lung-resident memory CD8 T cells (TRM) are indispensable for optimal cross-protection against pulmonary virus infection. J. Leukoc. Biol. *95*, 215–224.

Xing, Y.N., Xu, X.Y., Nie, X.C., Yang, X., Yu, M., Xu, H.M., Liu, Y.P., Takano, Y., and Zheng, H.C. (2012). Role and clinicopathologic significance of CXC chemokine ligand 16 and chemokine (C-X-C motif) receptor 6 expression in gastric carcinomas. Hum. Pathol. *43*, 2299–2307.

Yang, C.Y., Best, J.A., Knell, J., Yang, E., Sheridan, A.D., Jesionek, A.K., Li, H.S., Rivera, R.R., Lind, K.C., D'Cruz, L.M., et al. (2011). The transcriptional regulators Id2 and Id3 control the formation of distinct memory CD8+ T cell subsets. Nat. Immunol. *12*, 1221–1229.

Yu, G., Wang, L.G., Han, Y., and He, Q.Y. (2012). clusterProfiler: an R package for comparing biological themes among gene clusters. OMICS *16*, 284–287.

Zammit, D.J., Turner, D.L., Klonowski, K.D., Lefrançois, L., and Cauley, L.S. (2006). Residual antigen presentation after influenza virus infection affects CD8 T cell activation and migration. Immunity *24*, 439–449.

Zhou, X., and Xue, H.-H. (2012). Cutting edge: generation of memory precursors and functional memory CD8+ T cells depends on T cell factor-1 and lymphoid enhancer-binding factor-1. J. Immunol. *189*, 2722–2726.

Zhou, X., Yu, S., Zhao, D.-M., Harty, J.T., Badovinac, V.P., and Xue, H.-H. (2010). Differentiation and persistence of memory CD8(+) T cells depend on T cell factor 1. Immunity *33*, 229–240.

Zhou, X., Nurmukhambetova, S., Kim, Y.-C., Adair, P., Wu, C., Scott, D.W., Kuchroo, V.K., and Lees, J.R. (2016). Th17 cell differentiation increases aquaporin-3 expression, which is further increased by treatment with NaCl. J. Immunol. *196*, 186.116.

STAR★METHODS

KEY RESOURCES TABLE

REAGENT or RESOURCE	SOURCE	IDENTIFIER
Antibodies		
Purified Mouse monoclonal anti-CD16/32	Bio X Cell	Cat# BE0307; RRID:AB_2736987
Mouse monoclonal anti-CD4	Biolegend	Cat# 100510; RRID:AB_312713
Mouse monoclonal anti-CD8α	Biolegend	Cat# 100712; RRID:AB_312751
Mouse monoclonal anti-TCRβ	Biolegend	Cat# 109228; RRID:AB_1575173
Mouse monoclonal anti-TCRβ	Biolegend	Cat# 109206; RRID:AB_313429
Mouse monoclonal anti-CD44	Biolegend	Cat# 103012; RRID:AB_312963
Mouse monoclonal anti-CD103	Biolegend	Cat# 121418; RRID:AB_2128619
Mouse monoclonal anti-CD62L	Biolegend	Cat# 104428; RRID:AB_830799
Mouse monoclonal anti-CD69	Biolegend	Cat# 104512; RRID:AB_493564
Mouse monoclonal anti-CXCR6	Biolegend	Cat# 151108; RRID:AB_2572145
Mouse monoclonal anti-CD279	Biolegend	Cat# 109104; RRID:AB_313421
Mouse monoclonal anti-IL18R1	Biolegend	Cat# 132903; RRID:AB_2123952
Mouse monoclonal anti-CD127	ThermoFisher	Cat# 15-1271-82; RRID:AB_468793
Mouse monoclonal anti-CD45.1	Biolegend	Cat# 110708, RRID:AB_313497
Mouse monoclonal anti-CD45.2	Biolegend	Cat# 109830, RRID:AB_1186098
Mouse monoclonal anti-CXCL16	BD Biosciences	Cat# 566740; RRID:AB_2869842
Rat monoclonal IgG1κ	Biolegend	Cat# 400408; RRID:AB_326514
Cy3 AffiniPure Goat Anti-Rat IgG	Jackson ImmunoResearch	Cat# 112-165-175; RRID:AB_2338252
Mouse monoclonal CXCL16	Leinco Technologies	Cat# C1430; RRID:AB_2828491
Rat IgG2a isotype control	Bio X Cell	Cat# BE0089
Chemicals, peptides, and recombinant proteins		
TRI Reagent	Sigma	Cat# 93289
Human TNFα (Mouse Monoclonal)	Peprotech	Cat# 500-M26
Human IFNγ (Mouse Monoclonal)	Peprotech	Cat# 500-M90
GI254023X	Sigma	Cat# SML0789
Bouin's solution	Sigma	Cat# HT10132
Critical commercial assays		
Direct-zol RNA kit	Zymo Research	Cat# R2070
qScript Flex cDNA kit	Quanta bio	Cat# 95049-100
QIAquick Gel Extraction kit	QIAGEN	Cat# 28706
Chromium Single Cell 3′ GEM, Library & Gel Bead Kit v3	Illumina	Cat# PN-1000092
LIVE/DEAD Fixable Aqua Dead Cell Stain Kit	ThermoFisher	Cat# L34957
RNAqueous Micro RNA Isolation Kit	ThermoFisher	Cat# AM1931
Deposited data		
TCR Repertoire Data	This paper	https://data.mendeley.com/datasets/3f4rsk96kf/3
Single-cell RNA-Sequencing Data	This paper	https://data.mendeley.com/datasets/3f4rsk96kf/3
Experimental models: Cell lines		
Mouse: 4T1 cells	ATCC	CRL-2539
Experimental models: Organisms/strains		
Mouse: BALB/cJ	The Jackson Laboratory	JAX: 000651

(*Continued on next page*)

REAGENT or RESOURCE	SOURCE	IDENTIFIER
Continued		
Mouse: CByJ.SJL(B6)-*Ptprc*^{a}/J0	The Jackson Laboratory	JAX: 006584
Mouse: C.129S6(B6)-*Rag2*^{tm1Fwa} N12	Taconic	Taconic: 601-F
Oligonucleotides		
Primer: TCRβ constant region: 5′-ATCTCTGCTTCTGATGGCTCA-3′	This paper	N/A
Primer: Cxcl16 Forward: 5′-TGTGGAACTGGTCATGGGAAG-3′	This paper	N/A
Primer: Cxcl16 Reverse: 5′-AGCTTTTCCTTGGCTGGAGAG-3′	This paper	N/A
Software and algorithms		
FlowJo	BD Life Sciences	https://www.flowjo.com/
GraphPad Prism v7.03	GraphPad	https://www.graphpad.com/scientific-software/prism/
Zen Imaging	Zeiss	https://www.zeiss.com/microscopy/us/products/microscope-software/zen.html
ImageJ	Schneider et al., 2012	https://imagej.nih.gov/ij/
MiXCR v3.06	Bolotin et al., 2015	https://mixcr.readthedocs.io/en/master/
tcR	Nazarov et al., 2015	https://github.com/imminfo/tcr
R ggpubr	Wickham, 2016	https://rpkgs.datanovia.com/ggpubr/
R Seurat v2.3.4	Butler et al., 2018	https://github.com/satijalab/seurat/releases
Cellranger v2.02	10x Genomics	https://support.10xgenomics.com/single-cell-gene-expression/software/pipelines/latest/installation
Scanpy v1.4.1	Wolf et al., 2018	https://scanpy.readthedocs.io/en/stable/
SCENIC	Aibar et al., 2017	https://github.com/aertslab/SCENIC
Monocle v2.5.4	Trapnell et al., 2014	http://cole-trapnell-lab.github.io/monocle-release/
fastqc	Andrews, 2010	https://www.bioinformatics.babraham.ac.uk/projects/fastqc/
fastp	Chen et al., 2018	https://github.com/OpenGene/fastp
STAR v2.75	Dobin et al., 2013	https://github.com/alexdobin/STAR
featurecounts	Liao et al., 2014	http://subread.sourceforge.net/
DESeq2	Love et al., 2014	https://bioconductor.org/packages/release/bioc/html/DESeq2.html
ClusterProfiler	Yu et al., 2012	https://bioconductor.org/packages/release/bioc/html/clusterProfiler.html

RESOURCE AVAILABILITY

Lead contact

Further information and requests for resources and reagents should be directed to and will be fulfilled by the Lead Contact, Qi-Jing Li (Qi-Jing.Li@Duke.edu).

Materials availability

This study did not generate any unique reagents.

Data and code availability

The TCR repertoire and single-cell RNA sequencing datasets generated during this study is available at: https://doi.org/10.17632/3f4rsk96kf.3, https://data.mendeley.com/datasets/3f4rsk96kf/3.

EXPERIMENTAL MODEL AND SUBJECT DETAILS

Animals

BALB/c mice (BALB/cJ) and congenically marked CD45.1$^+$ BALB/c mice (CByJ.SJL(B6)-*Ptprc*^{a}/J0) were purchased from The Jackson Laboratory. Rag2-KO BALB/c mice (C.129S6(B6)-*Rag2*^{tm1Fwa} N12) were purchased from Taconic. All mice were housed under pathogen-free conditions and only female mice were used between 6-10 weeks for experimental procedures. Littermates of the same sex were randomly assigned to experimental groups. All mice were used in accordance with Institutional Animal Care and Use Committee guidelines at Duke University.

Cell lines

The 4T1 mammary carcinoma cell line was a gift from Xiao-Fan Wang (Duke University); the cell line was authenticated prior to their use in experiments. 4T1 tumor cells were grown in coordination with ATCC guidelines: ATCC-formulated-RPMI-1640 Medium (ATCC 30-2001) was used and supplemented with 10% fetal bovine serum. Cells were grown at 37°C with 5% CO_2 in 100mm cell culture dishes (VWR). Cells were subcultured at 80% confluence at a ratio of 1:6.

METHOD DETAILS

Tumor model and tissue isolation

Only female mice were used in all experiments. The 4T1 mammary carcinoma cell line was a gift from Xiao-Fan Wang (Duke University). Tumor cells were harvested by trypsinization, and cell viability was evaluated by trypan blue exclusion. 100 4T1 cells in 10 μL of serum-free media were orthotopically injected directly into the mammary gland of anesthetized Female BALB/c mice using a microsyringe with a 26-gauge needle (Hamilton Company, Reno, NV). Tumor progression was monitored closely and tumor growth kinetics were measured. Mice were sacrificed at three weeks post tumor injection for all tumor-cell sorting experiments and their tumors, tumor mammary mucosa, and contralateral (distant) mammary mucosa tissue were harvested. The primary tumor was first removed, followed by dissection of the remaining mucosa tissue surrounding the tumor (tumor mucosa). Care was taken during the dissection process to ensure that the inguinal lymph nodes were removed prior to mammary mucosa tissue harvest. All tissues were mechanically homogenized and filtered over 70 μm nylon mesh filters (VWR) to obtain a single cell suspension for downstream assays. Enzymatic digestion was avoided as to eliminate the possibility that antibody binding sites could be degraded.

Tumor T_{RM} characterization

To characterize the developmental time-course of tumor T_{RM}s, mice were sacrificed either 2 weeks, 3 weeks or 4 weeks post 4T1 tumor injection. Tumors were harvested and homogenized into a single suspension. Isolated cells were blocked with anti-mouse CD16/CD32 Fc Block (2.4G2) for 10 minutes prior to antibody staining. Cells were stained with antibodies to CD4 (RM4-5), CD8α (53-6.7), TCRβ (H57-597), CD44 (IM7), CD103 (2E7), CD62L (MEL-14) and CD69 (H1.2F3). All data was acquired on a BD FacsCanto flow cytometer (BD Biosciences) and analyzed using FlowJo software (Treestar).

TCRβ repertoire sequencing

Tumor, tumor-adjacent mammary mucosa, contralateral mammary mucosa, draining lymph nodes and spleen were isolated from 4T1 tumor bearing mice at 3-weeks post tumor injection and lysed in TRIzol Reagent (Sigma Aldrich). RNA was extracted using the Direct-zol RNA kit (Zymo Research) according to the manufacturer's instructions. cDNA was synthesized using the qScript Flex cDNA synthesis kit (Quanta Biosciences) with a constant region specific primer (5′-ATCTCTGCTTCT- GATGGCTCA-3′). Multiplex PCR was performed to amplify the CDR3 region of rearranged *TCRB* loci and a set of primers, each specific to a specific TCR Vβ segments, and a reverse primer to the constant region of *TCRB* were used to generate a library of amplicons that cover the entire CDR3 region. PCR products were loaded on agarose gels and bands between 220-240 bp were extracted and purified using the QIAquick Gel Extraction kit (QIAGEN). These purified products were sequenced using the Illumina HiSeq X Ten machine.

TCRβ repertoire analysis

Sequence data were analyzed with MiXCR (v3.06) (Bolotin et al., 2015). This software first aligned sequence short reads to reference T cell receptors, then extracted CDR3 sequences and exported TCR clonotypes. The *tcR* package under the R computing environment was used for clonotype summary, repertoire diversity, and similarity analysis. In these analyses, out-of-frame TCR clonotypes were excluded (Nazarov et al., 2015). Figures were plotted using R *ggpubr*.

10x Genomics library preparation and sequencing

Libraries were prepared following the 10x Genomics Single Cell 3′ protocol. Single cells were dissociated, washed and resuspended in a 1x PBS/0.04% BSA solution at a concentration of 1000 cells/μl to remove dead cells and contaminants. A Cellometer (Nexcelom) was used to determine cell viability and cells were normalized to 1 × 10^6 cells/mL. Cells were then combined with a master mix including reverse transcription reagents. With this, gel beads carrying the Illumina TruSeq Read 1 sequencing primer, a 16bp 10x barcode, a 12bp unique molecular identifier (UMI) and a poly-dT primer were loaded onto the chip, together with oil for the emulsion

reaction. Reverse transcription occurs in nanoliter-scale gel beads in emulsion (GEMs) so that all cDNAs within a GEM share a common barcode. After this reverse transcription reaction, the GEMs were broken and full length cDNA purified with Silane Dynabeads and SPRI beads then assayed on an Agilent 4200 TapeStation High Sensitivity D5000 ScreenTape (Santa Clara, CA) for qualitative and quantitative analysis. Illumina P5 and P7 sequences (San Diego, CA), a sample index and TruSeq read 2 primer sequences were added via End Repair, A-tailing, Adaptor Ligation and PCR. Sequences were generated using paired end sequencing on an Illumina sequencing platform at a minimum of 50,000 reads/cell.

Single-cell RNA-seq analysis

Raw short reads were demultiplexed, filtering and mapped to mouse genome GRCm38/mm10 using cellranger v2.02. The gene count matrices from cellranger were subjected to quality control, pre-processing and clustering using the R Seurat 2.3.4 package (Butler et al., 2018). Low-quality cells that had less than 200 expressed genes and more than 5% mitochondrial genes were filtered out. Gene counts were scaled to total gene expression and percentage of mitochondrial genes with a scaling factor of 10,000, and then log-transformed. The high dimensional data for each sample were reduced by PCA and t-Distributed Stochastics Neighbor Embedding (*tSNE*). We used the FindCluster function to group clusters in each sample with a resolution of 0.6. Differential expressed genes (DEGs) were identified using the Wilcoxon rank-sum test. The python package *scanpy* v 1.4.1 (Wolf et al., 2018) was used to integrate analysis for all samples following the common procedure. The expression matrix was normalized and log transformed through scanply.pp.log1p function, and high variable genes were chosen by scanpy.pp.highly_variable_genes. SCENIC (Aibar et al., 2017) was used to perform the gene regulatory network (regulon) analysis. This algorithm contained three major steps: 1) find the gene co-expression modules between transcription factors and target genes using GENIE3 (R package); 2) identify co-expression modules between cis-regulatory motif and their target genes using RcisTarget; 3) score each regulon through AUCell to select the top regulons in each sample.

For our Genetrac analysis, which plots the gene expression values in each measured cell across pre-defined groups, we used the *scanpy* toolkit but did not use the option of hierarchical clustering, so the cells within each group were ordered randomly. To infer the lineage development of $T_{EFF\ /\ EM}$ to T_{CM}, we used Monocle package v2.5.4 (Qiu et al., 2017). The top 2000 significant DEGs were chosen to order genes for trajectory reconstruction using the DDRTree method followed by dimension reduction, cell trajectory inference and pseudo-time measurements, which were computed via reversed graph embedding.

Bulk RNA-sequencing

Tumors were harvested from 4T1 tumor bearing mice and homogenized into a single cell suspension. Isolated cells were stained with a LIVE/DEAD Fixable Aqua stain (Thermo Fisher) and blocked with anti-mouse CD16/CD32 Fc Block (2.4G2) for 10 minutes prior to antibody staining. Cells were stained with antibodies to CD8α (53-6.7), TCRβ (H57-597), CD44 (IM7), CD127 (A7R34), IL18R (BG/IL18RA), CD279 (RMPI-30), and CXCR6 (SA051D1) and sorted using the MoFlo Astrios cell sorter. Depending on the experimental yield for 4 replicates, $2.5x10^4 – 5x10^4$ of each T_{EM} precursor population were sorted and directly lysed in Lysis Buffer. RNA was extracted using the RNAqueous Micro RNA Isolation Kit (Thermo Fisher) according to the manufacturer's instructions. RNA samples were evaluated for concentration by Qubit (Thermo Fisher) and integrity using an Agilent 2100 Bioanalyzer. Clontech Ultra low libraries were prepared and sequenced using the Illumina HiSeq platform.

Bulk RNA-sequencing analysis

High-throughput short reads were trimmed, filtered, mapped, and counted. The sequence quality was assessed using *fastqc* software (Andrews, 2010) and filtered using *fastp* (PMID: 30423086 or Chen et al., 2018). Reads were mapped to mouse reference M23 (Genecode) using *STAR* software (PMID: 23104886 or Dobin et al., 2013). For RNA-seq, read counts per gene were generated using *featureCounts* (PMID: 24227677 or Liao et al., 2014). *DESeq2* was used to perform counts normalization and test for differential expressed genes (Love et al., 2014). Gene ontology (GO) enrichment analysis was computed using ClusterProfiler (Yu et al., 2012).

Adoptive transfer of T_{EM} precursor cells

Tumors were harvested from female CD45.1$^+$ BALB/c mice bearing 3 week established 4T1 tumors and homogenized into a single cell suspension. Isolated cells were stained with a LIVE/DEAD Fixable Aqua stain (Thermo Fisher) and blocked with anti-mouse CD16/CD32 Fc Block (2.4G2) for 10 minutes prior to antibody staining. Cells were stained with antibodies to CD8α (53-6.7), TCRβ (H57-597), CD44 (IM7), CD127 (A7R34), IL18R (BG/IL18RA), CD279 (RMPI-30), and CXCR6 (SA051D1) and sorted using the MoFlo Astrios cell sorter. $1.5x10^4 – 2x10^4$ cells of each T_{EM} precursor population were sorted depending on the yield of the individual experiment. These cells were directly transferred orthotopically into the mammary gland of 2-week tumor-bearing Rag2KO BALB/c mice. Two weeks later, animals were sacrificed and stained with antibodies to CD8α (53-6.7), TCRβ (H57-597), CD44 (IM7), CD103 (2E7), CD45.1 (A20) and CD45.2 (104) for flow cytometry analysis on BD FACSCanto flow cytometers (BD Biosciences) and analyzed with FlowJo software (Treestar).

CXCL16 qPCR

Tumor, tumor-adjacent mammary mucosa, contralateral mammary mucosa, draining lymph nodes and spleen were isolated from 4T1 tumor bearing mice and lysed in TRIzol Reagent (Sigma Aldrich). RNA was extracted using the Direct-zol RNA kit (Zymo

Research) according to the manufacturer's instructions. After annealing oligo-DT primers, cDNA was synthesized using the qScript Flex cDNA synthesis kit (Quanta Biosciences), according to the manufacturer's instructions. SYBR-Green based real-time PCR method was used to quantify the relative expression of *Cxcl16* mRNA and three housekeeping genes: *Hprt, Sdha, Ywhaz*.

Cxcl16-F 5′-TGTGGAACTGGTCATGGGAAG-3′
Cxcl16-R 5′-AGCTTTTCCTTGGCTGGAGAG-3′
Hprt-F 5′-AGTGTTGGATACAGGCCAGAC-3′
Hprt-R 5′-TGCGCTCATCTTAGGCTTTGT-3′
Sdha-F 5′-TTATTGCTACTGGGGGCTACGGG-3′
Sdha-R 5′-AGGCAGCCAGCACCGTATATACC-3′
Ywhaz-F 5′- ACGCTCCCTAACCTTGCTTC-3′
Ywhaz-R 5′-ACACACCGAACTGTTGTCGT-3′

CXCL16 flow cytometry

4T1 tumor cells were cultured for 24 hours in media containing 20 ng/mL TNFα and 20 ng/ml IFNγ cytokines (Peprotech) in the presence of 1 μM ADAM10 inhibitor to prevent membrane shedding (GI254023X, Sigma). Cells were dissociated with an enzyme-free dissociation buffer and homogenized to a single cell suspension. Mucosal epithelial cells were harvested directly from a tumor naive BALB/c mouse and were mechanically homogenized and filtered over 70 μm nylon mesh filters (VWR) to obtain a single cell suspension. Isolated cells were stained with a LIVE/DEAD Fixable Aqua stain (Thermo Fisher) and blocked with anti-mouse CD16/CD32 Fc Block (2.4G2) for 10 minutes prior to antibody staining. Cells were stained with either CXCL16 antibody (12-81, BD Biosciences) or isotype control (Rat IgG1κ, Biolegend). All data was acquired on a BD FacsCanto flow cytometer (BD Biosciences) and analyzed using FlowJo software (Treestar).

Confocal microscopy

Fresh tissue samples from 4T1 tumors were cryosectioned into 20 μm sections followed by immediate fixation in 4% PFA for 20 minutes. Tissue slides were immunostained with a primary antibody against CXCL16 (12-81, BD Biosciences) followed by a Cy 3 Goat Anti-Rat IgG secondary antibody (Jackson ImmunoResearch), along with control slides which did not receive secondary antibody stain. Microscopic images were acquired with a confocal microscope (Carl Zeiss 710 inverted) using a 20x objective. Images were construction with z stacks of images using Zen imaging software (Zeiss).

Antibody blocking experiments, lung H&E analysis

BALB/c mice bearing 4T1 tumors were intratumorally injected with either 100 μg anti-CXCL16 (142417, Leinco Technologies), with or without 200 μg anti-Thy1.2 (30H12, BioXCell) or 500 μg IgG2a isotype antibody (2A3, BioXCell) at days 7, 14 and 21 post tumor injection. Primary tumors were surgically removed at day 25 and mice were monitored for their humane endpoint. At this time, lungs were harvested and placed into Bouin's solution fixative for 48 hours for visualization and quantification of lung tumor nodules. A representative subset of lungs (n = 3) were placed in a 70% ethanol aqueous solution and routine hematoxylin and eosin (H&E) staining was performed. Each sample was sectioned into 5 slices and independently analyzed by three oncologists to evaluate the total area of the lung harboring tumor metastases. The metastatic tumor area and total lung area were first calculated separately, then the tumor area was divided by total lung area; resultant data were represented as a percentage of the area of the lung harboring metastases. All image processing analysis was performed in ImageJ software (NIH).

QUANTIFICATION AND STATISTICAL ANALYSIS

All statistical analysis was carried out using GraphPad v7.03 and R programming language. Specific statistical methods used for analysis are detailed in the text. A p value of less than 0.05 was set as statistically significant.

Cell Reports, Volume *35*

Supplemental information

Resident memory T cells in tumor-distant tissues fortify against metastasis formation

Laura S. Christian, Liuyang Wang, Bryan Lim, Dachuan Deng, Haiyang Wu, Xiao-Fan Wang, and Qi-Jing Li

Resident memory T cells in tumor-distant tissues fortify in metastasis formation

Laura S Christian[1†], Liuyang Wang[2†], Bryan Lim[1], Dachuan Deng[3], Haiyang Wu[3], Xiao-Fan Wang[4], Qi-Jing Li[1*]

Supplemental Information

Supplemtentary Figures 1-5
Supplementary Tables 1-3

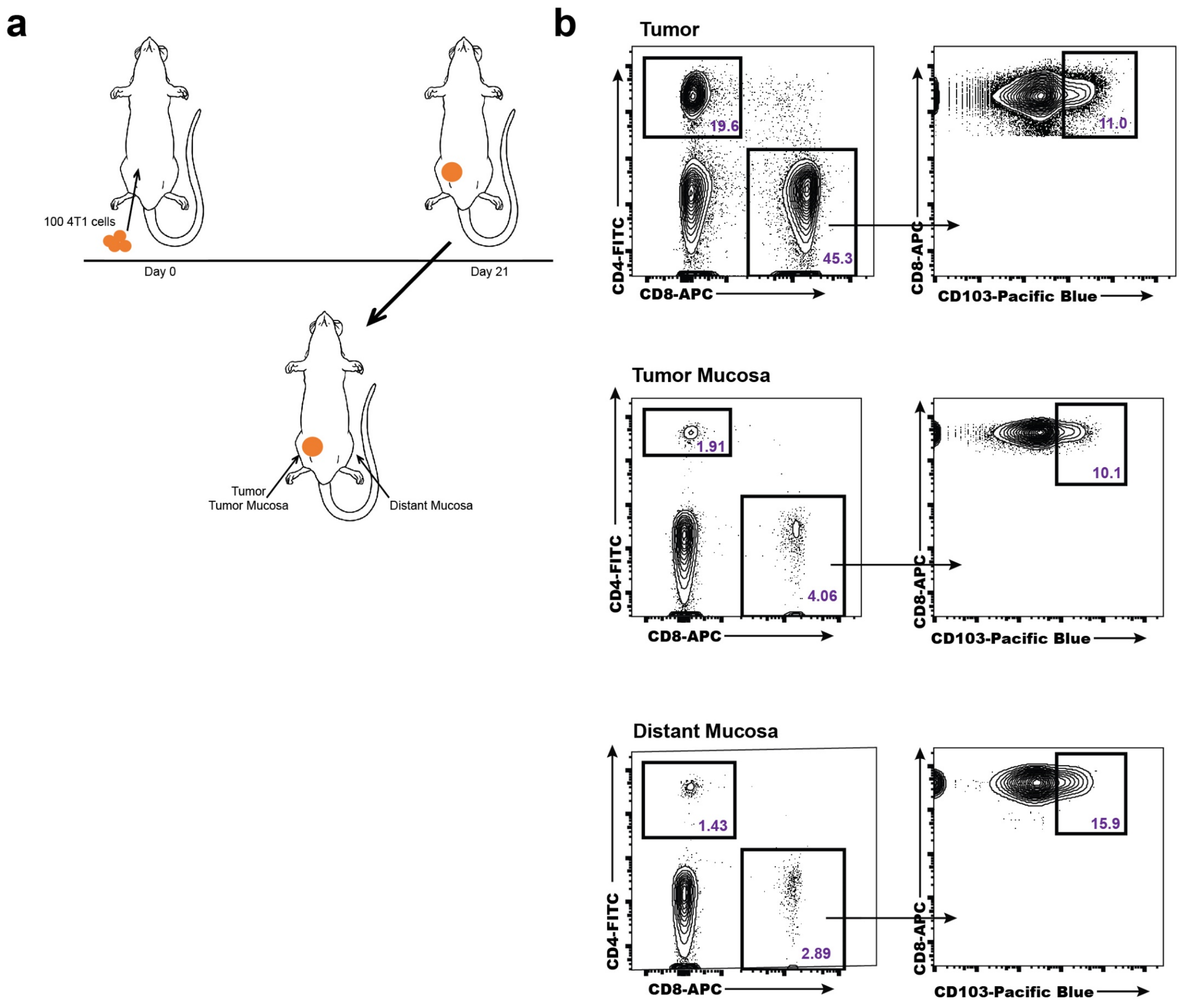

Figure S1. Related to Figure 1: Experimental design for tumor T_{RM} generation and sample collection. a, Experiments using titrating amounts of 4T1 tumor cells revealed that orthotopic injection of 100 4T1 cells into the mammary fat pad of 4T1 mice caused robust tumor formation at 21 days post injection. The diagram shows the location of the tumor, tumor mucosa and distant mucosa, the main sites of sample collection. **b,** Representative flow cytometry plots and gating of T_{RM}s in the tumor, tumor mucosa and distant mucosa at d21 post tumor cell injection.

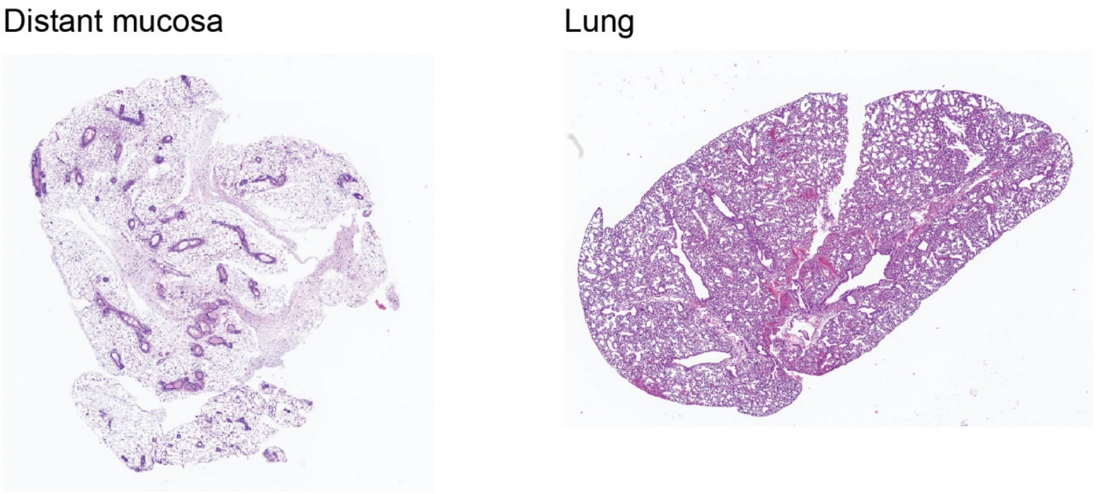

Figure S2. Related to Figure 1: Metastatic tumors are not found in the distant mucosa or lung 3 weeks post tumor implantation. Representative H&E images of distant mucosa or lung harvested from 4T1 tumor-bearing mice 3 weeks post tumor implantation reveals there are no tumor metastases present at this early time-point.

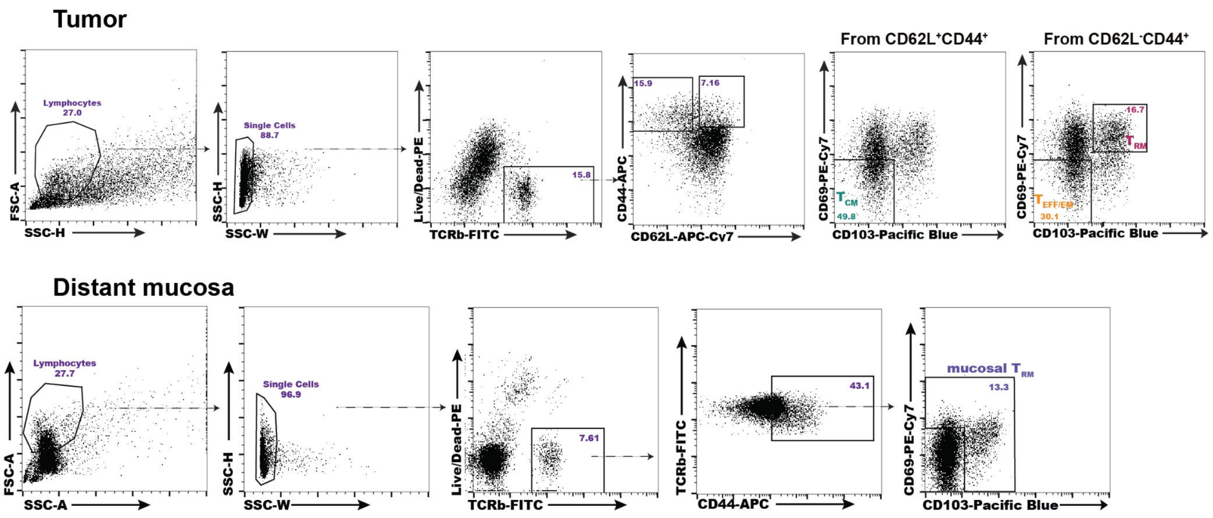

Figure S3. Related to Figure 2: Sorting strategy for single cell RNA-sequencing sample collection. Representative plots showing the flow cytometry sorting strategy for tumor T_{EM}s, T_{CM}s, T_{RM}s and distant mucosa T_{RM}s for single-cell RNA-sequencing sample collection. Tumors were harvested 21 days after tumor injection and cells were stained for various cell surface markers as detailed above.

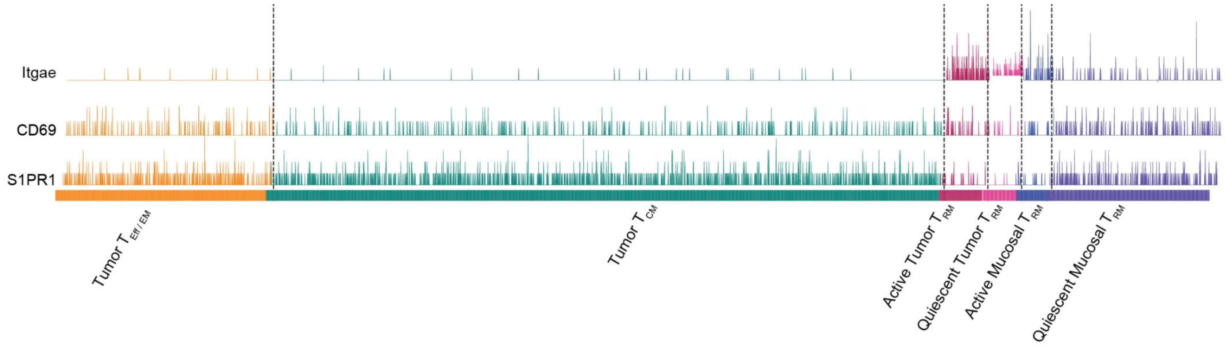

Figure S4. Related to Figure 3: Gene expression of T_{RM} markers confirms heterogeneity of tumor T_{RM}s. GeneTrac analysis of T_{RM} defining marker *Itgae* (CD103) is upregulated only in the T_{RM} populations, concomitant with reduced *S1pr1* expression.

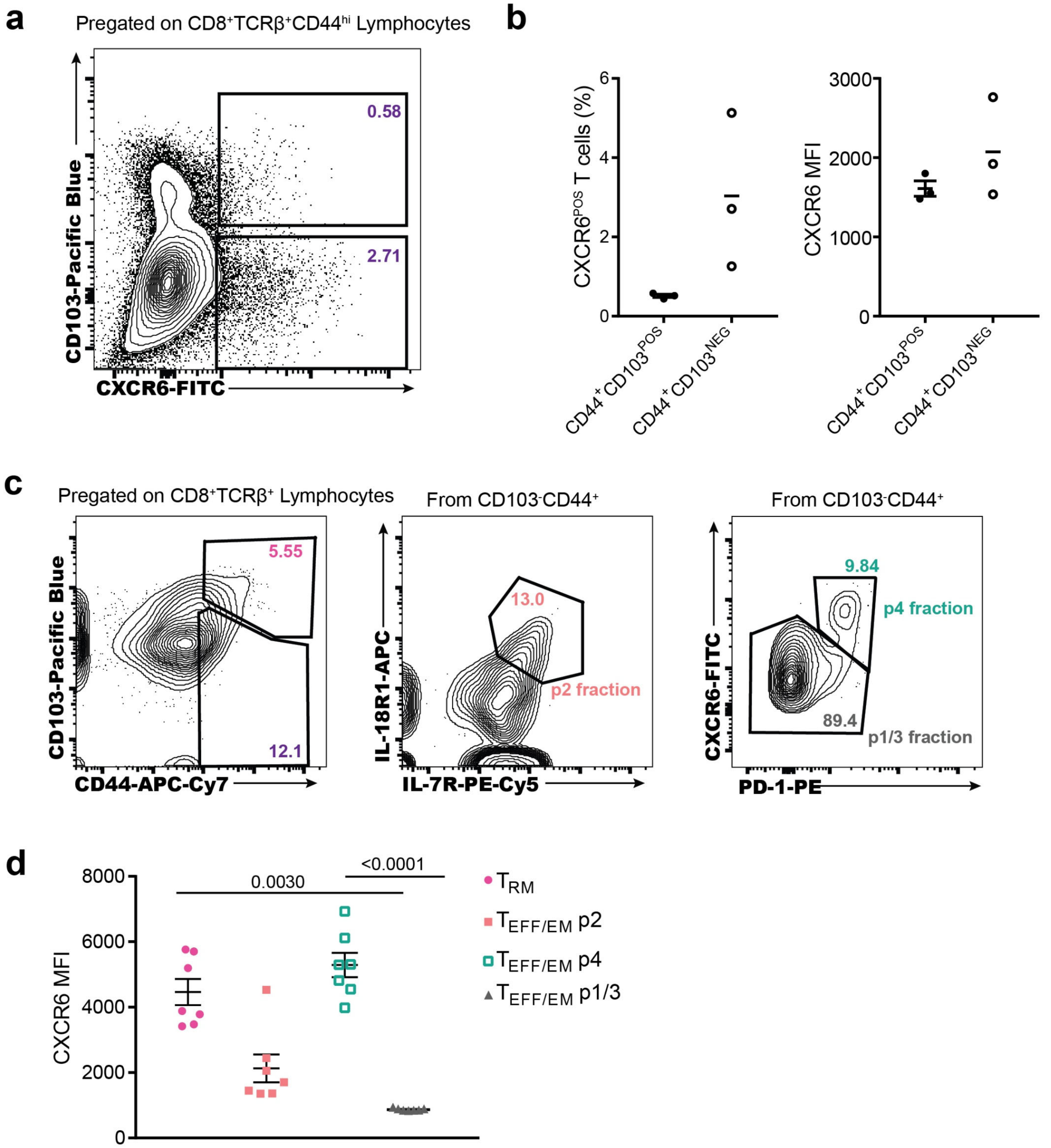

Figure S5. Related to Figure 5: Intra-tumoral $T_{Eff/EM}$ p4 cells preferentially express CXCR6. a, Representative flow plot on 4T1 tumor shows that intra-tumor $T_{Eff/EM}$s express more CXCR6 than T_{RM}s. These data are graphed by **b,** the percentage of $CD44^{+}CD103^{POS/NEG}$ cells expressing $CXCR6^{+}$ and by CXCR6 MFI. **c,** Representative flow plots of 4T1 tumor shows that the intra-tumor $T_{Eff/EM}$ p4 population expresses more CXCR6 than $T_{Eff/EM}$ p2. These data are graphed by **d,** CXCR6 MFI. Symbols represent individual mice and error bars represent mean ± s.e.m. $p<0.05$, Kruskal-Wallis test with multiple comparisons.

Supplementary Table 1: QC analysis of TCRβ repertoire sequencing samples related to Figure 1.

Sample ID	Total RNA	Total Mapped Reads	Unique TCR Clonotypes	Mean Read Per TCR
Tumor 1	500ng	84329	2626	32.11
Tumor 2	500ng	37368	2236	16.71
Tumor 3	500ng	82313	24636	3.34
Tumor Mucosa 1	500ng	16869	91	185.37
Tumor Mucosa 2	500ng	36775	1244	29.56
Tumor Mucosa 3	500ng	15249	70	217.84
Distant Mucosa 1	500ng	27106	88	308.02
Distant Mucosa 2	500ng	20882	135	154.68
Distant Mucosa 3	500ng	12359	191	64.71
WT Mucosa 1	300ng	138109	1299	106.32
WT Mucosa 2	300ng	121488	25145	4.83
WT Mucosa 3	300ng	4404	1392	3.16
dLN 1	500ng	74528	26892	2.77
dLN 2	500ng	62460	28414	2.2
dLN 3	500ng	39266	1781	22.05
Spleen 1	500ng	61992	27290	2.27
Spleen 2	500ng	64709	28596	2.26
Spleen 3	500ng	75714	28323	2.67

Supplementary Table 2: Sequencing information for samples submitted for scRNA-seq related to Figure 2.

Sample ID	No. Cells	Mean Reads/Cell	Median Genes/Cell	No. Reads	Valid Barcodes
Tumor T_{RM}	2273	473741	1228	1076814147	0.969
Tumor T_{Eff} / T_{EM}	6859	122127	1060	837674652	0.976
Tumor T_{CM}	9424	77281	1127	728297585	0.977
Distant Mucosa T_{RM}	3679	50324	1155	185144103	0.971

Sample ID	Sequencing Saturation	Q30 Bases in UMI	Reads Mapped to Genome	Total Genes Detected	Median UMI Counts/Cell
Tumor T_{RM}	0.971	0.959	0.864	14361	2915
Tumor T_{Eff} / T_{EM}	0.935	0.96	0.899	15152	2877
Tumor T_{CM}	0.883	0.96	0.9	15529	3659
Distant Mucosa T_{RM}	0.845	0.979	0.944	14616	3271

Supplementary Table 3: QC analysis of TCRβ repertoire sequencing samples related to Figure 6.

Sample ID	Cells Sorted	Total Mapped Reads	Unique TCR Clonotypes	Mean Read Per TCR
CXCR6+	8769	21410	6414	3.34
CXCR6-	9674	15129	5529	2.74
Tumor Trm	13000	113246	11730	9.65
Distant Trm	11600	15554	4074	3.82
